# PRRSV suppresses FTO-dependent *m6A* demethylation to reprogram STAT signaling and innate immunity

**DOI:** 10.1128/mbio.00843-26

**Published:** 2026-08-13

**Authors:** Yipeng Pang, Fructueux Modeste Amona, Yuan Liang, Xiaohan Chen, Ran Tao, Zilu Liu, Shiqing Yang, Yuenan Wang, Wenxin Chen, Xinchi Xia, Rong Zhou, Bin Li, Baochao Fan, Xingtang Fang, Xi Chen

**Affiliations:** 1 School of Life Science, Institute of Cellular and Molecular Biology, Jiangsu Normal University 12675, Xuzhou, Jiangsu, China; 2 Institute of Veterinary Medicine, Jiangsu Academy of Agricultural Sciences 668638, Nanjing, China; Boston University Chobanian & Avedisian School of Medicine, Boston, Massachusetts, USA

**Keywords:** PRRSV, *m6A *modification, FTO, innate immunity, interferon signaling, epitranscriptomics

## Abstract

RNA viruses have evolved diverse strategies to evade host interferon (IFN)-stimulated gene (ISG) defenses; however, how they exploit host epitranscriptomic regulation remains poorly understood. Here, we identify an immune-evasion mechanism in which porcine reproductive and respiratory syndrome virus (PRRSV) targets the *m6A* demethylase fat mass and obesity-associated protein (FTO) to suppress antiviral signaling. Mechanistically, the viral endoribonuclease nsp11 inhibits STAT5-dependent transcription through the key residues Q96 and S104, thereby reducing FTO expression. Loss of FTO increases *m6A* modification of STAT2 and STAT3 transcripts, impairing their translation and phosphorylation, thereby attenuating ISG responses. Reduced STAT3 activity further dampens STAT5 signaling, establishing a feed-forward circuit that amplifies suppression of antiviral immunity. Functionally, disruption of this regulatory region (Q96A and S104A) attenuates viral pathogenicity *in vivo* and restores ISG induction. These mutations also reduce infection-associated inflammatory responses and the accumulation of reactive oxygen species. Together, these findings define a nsp11-STAT5-FTO-STAT2/3 axis that enables PRRSV to reprogram host epitranscriptomic control of innate immunity. Our work reveals a mechanism of epitranscriptomic hijacking and identifies FTO as a key host factor exploited by RNA viruses, highlighting *m6A* regulation as a potential target for antiviral intervention.

**IMPORTANCE:**

Viruses must overcome host innate immune defenses to establish infection; however, the mechanisms by which they manipulate host RNA regulation remain incompletely understood. In this study, we show that porcine reproductive and respiratory syndrome virus (PRRSV) suppresses interferon responses by targeting the host *m6A* demethylase FTO through its endoribonuclease nsp11. This process involves the inhibition of STAT5 phosphorylation, which reduces FTO expression and increases *m6A* modification of key immune regulators, including STAT2 and STAT3, thereby impairing their activation. Disruption of this pathway attenuates viral pathogenicity *in vivo* and restores antiviral signaling. These results demonstrate that PRRSV can reprogram host epitranscriptomic regulation to modulate innate immunity and suggest that *m6A*-related pathways may be potential targets for antiviral intervention.

## INTRODUCTION

The ongoing evolutionary battle between RNA viruses and their hosts centers on the manipulation of the innate immune system (1, 2). Type I interferons (IFNs) represent the host’s frontline defense, inducing hundreds of interferon-stimulated genes (ISGs) that collectively establish an antiviral state (3, 4). To counteract this, effective RNA viruses, such as Flaviviruses (5), Coronaviruses (6), and Paramyxoviruses (7), have independently evolved sophisticated strategies to suppress the IFN-ISG pathway. This convergent targeting underscores the IFN-ISG pathway as a universal vulnerability. It suggests that successful immune evasion likely involves multilayer host-cell reprogramming that extends beyond transcriptional control to post-transcriptional and epitranscriptomic regulation.

Among post-transcriptional mechanisms, RNA modifications, particularly *m6A*, have emerged as a pivotal regulatory layer in viral-host interactions (8, 9). As the most prevalent internal mark in eukaryotic mRNA, *m6A* dynamically shapes RNA stability, translation, and immunogenicity through the coordinated actions of methyltransferase “writers,” binding protein “readers,” and demethylase “erasers” (10, 11). The critical nature of this epitranscriptomic machinery is evident across pathologies; in a non-viral context, CircHECW2 regulates astrocyte function by mediating *m6A* methylation of Gng4 mRNA (12). This modification forms a shared molecular language: the host uses *m6A* to tag viral RNAs for degradation, whereas many RNA viruses hijack the same machinery to stabilize their genomes and dampen antiviral signaling (13, 14). Thus, the balance within this “write-read-erase” cycle determines whether infection is cleared or sustained. However, while the functions of *m6A* writers and readers are increasingly well-characterized, the regulation and biological significance of demethylases (“erasers”) during viral infection remain poorly defined.

A further unresolved issue lies in the frequent global *m6A* hypermethylation observed across diverse viral infections (8, 13). Whether this phenomenon reflects a passive host stress response or an active viral strategy remains controversial. The pathological significance of such epitranscriptomic imbalance is further highlighted by findings that a genetic variant in the TAPBP gene enhances cervical cancer susceptibility by increasing *m6A* modification (15), underscoring the importance of precise *m6A* homeostasis for disease prevention. The fat mass and obesity-associated protein (FTO), a principal *m6A* demethylase involved in metabolism and stress responses (16, 17), is a compelling candidate target for viral manipulation. However, its role in antiviral immunity has been largely overlooked. Thus, addressing whether RNA viruses actively suppress FTO—and delineating the downstream consequences of such suppression—would substantially advance understanding of how viruses exploit the host epitranscriptome to escape immune restriction.

**Fig 1:**
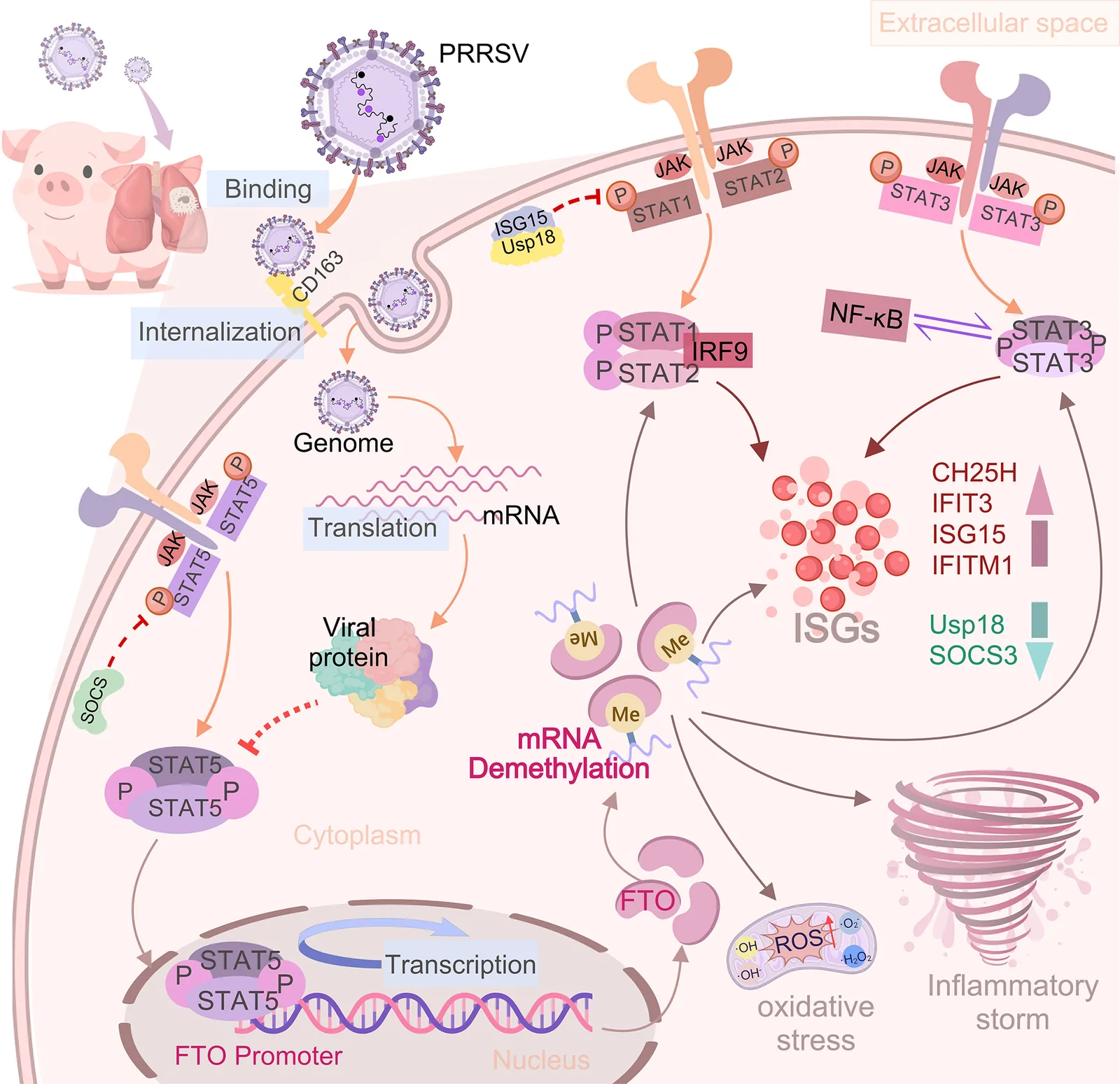
PRRSV suppresses FTO to reprogram *m6A*-dependent STAT signaling and innate immune responses. PRRSV nsp11 inhibits STAT5-driven FTO transcription, inducing *m6A* hypermethylation of STAT2/STAT3 mRNAs and preventing their phosphorylation, thereby impairing STAT2/3-mediated ISG transcription and interferon signaling.

To address how RNA viruses exploit *m6A* demethylase regulation to subvert host immunity, we employed porcine reproductive and respiratory syndrome virus (PRRSV) as a representative enveloped + ssRNA virus model that causes substantial economic losses in the swine industry. PRRSV exemplifies immune-evasion strategies shared by many RNA viruses, displaying a paradoxical phenotype characterized by excessive inflammation alongside suppression of the IFN-ISG response. Such dysregulated immune responses are frequently coupled with cellular stress states, as immune signaling pathways often exhibit coordinated regulation with inflammatory and redox-associated processes. For instance, suppression of JAK2/STAT3 signaling via reduced O-GlcNAcylation of STAT3 in intestinal epithelial cells highlights the intricate interplay between post-translational regulation and inflammatory signaling (18). PRRSV’s ability to infect both porcine and simian cells further provides a robust cross-species platform to dissect conserved virus-host interactions. Thus, we hypothesize that RNA viruses such as PRRSV evade host antiviral defenses by suppressing the *m6A* demethylase FTO, thereby disrupting *m6A* homeostasis and impairing activation of the IFN-ISG axis (Fig. 1). Given this hypothesis, this study integrates molecular, transcriptomic, and reverse-genetic approaches to (i) characterize PRRSV-induced changes in FTO expression and localization, (ii) identify the specific viral factor driving this modulation, and (iii) elucidate the downstream consequences on *m6A*-dependent immune signaling. Altogether, this study aims to establish a conceptual framework in which viral proteins induce host epitranscriptomic remodeling that not only suppresses antiviral immunity but also contributes to broader immune and stress-associated dysregulation. While focusing on PRRSV, the conserved nsp11 endoribonuclease suggests that similar epitranscriptomic hijacking strategies may be employed by other RNA viruses, providing a basis for future investigation.

## RESULTS

### PRRSV induces immune dysregulation and cellular stress with *m6A* remodeling

**Fig 2:**
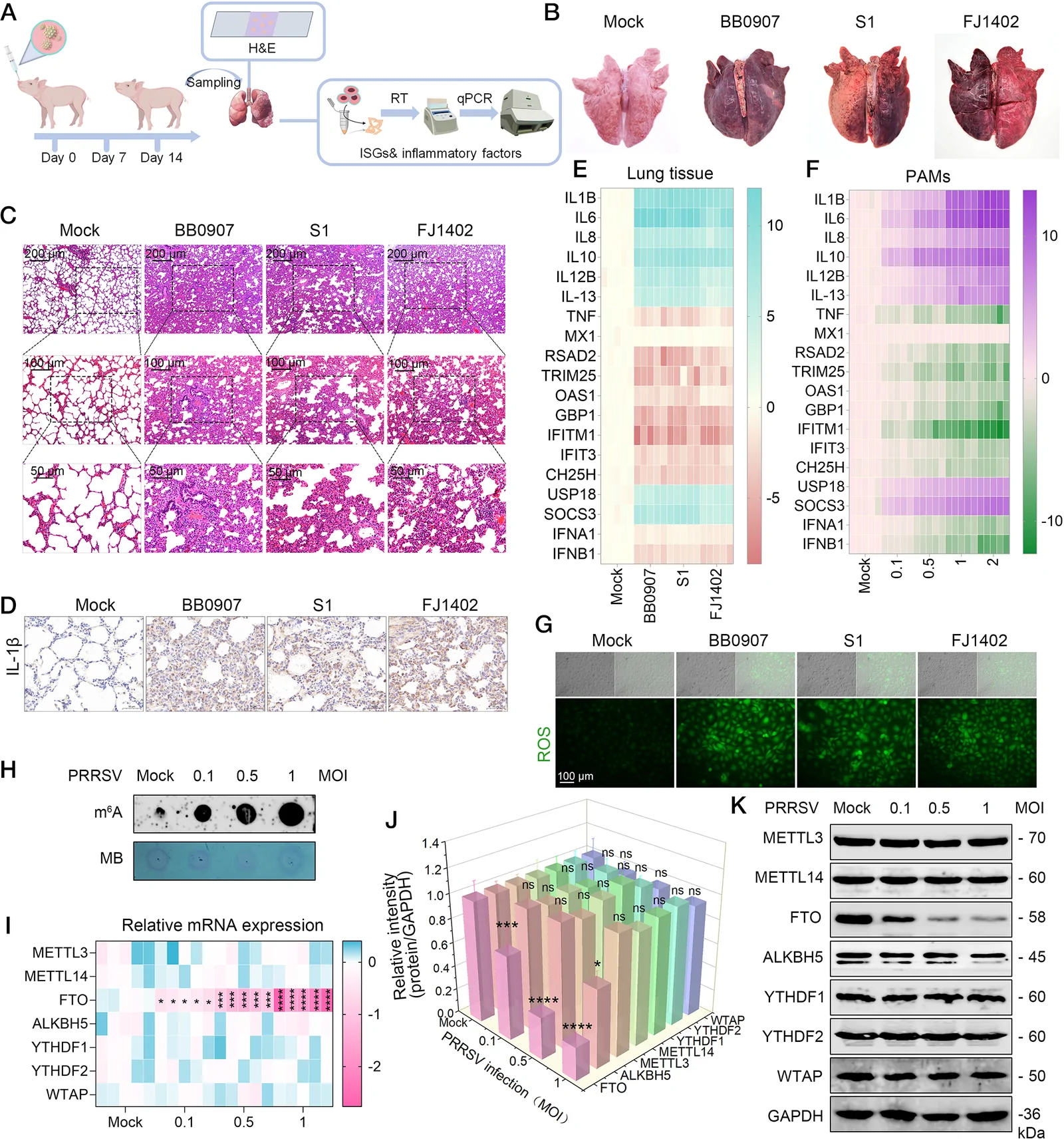
PRRSV triggers inflammatory and oxidative stress responses accompanied by *m6A* epitranscriptome remodeling. (**A**) Schematic diagram for assessing PRRSV strain-induced lung inflammation in 6-week-old specific-pathogen-free (SPF) piglets that received intranasal inoculation with PRRSV strains (BB0907, S1, and FJ1402) or medium (Mock), and lung tissues were harvested 14 days post-infection. (**B**) Gross pathological examination of lung tissue surface lesions. (**C**) Histopathological analyses of pig lungs. (**D**) Immunohistochemical staining of IL-1β in lung tissues from piglets infected with different PRRSV strains. Scale bar = 50 µm. (**E and F**) Heatmap of mRNA expression levels of inflammatory cytokines and ISGs in lung tissue (**E**) and in PAMs (**F**). (**G**) Intracellular ROS levels detected by fluorescence imaging in PAMs following PRRSV infection. (**H**) Dot blot analysis of *m6A* RNA modification levels in infected PAMs, with methylene blue (MB) staining as a loading control. (**I**) Heatmap depicting the relative mRNA expression of key *m6A* regulators, METTL3, METTL14, FTO, ALKBH5, YTHDF1, YTHDF2, and WTAP, in PRRSV-infected PAMs. (**J and K**) Western blot (**K**) and quantification analysis (**J**) of *m6A* regulator protein expression in PAMs infected with different MOIs. GAPDH served as a loading control. (**B–K**) *n* = 5 biologically independent samples.

PRRSV modulates immune defenses through multiple mechanisms, thereby triggering severe lung tissue damage; however, its potential to regulate the host epitranscriptome and stress responses remains unclear. To investigate this, we first established an intranasal porcine infection model using three PRRSV strains (S1, highly pathogenic BB0907, and FJ1402) and collected lung samples at 14 dpi (Fig. 2A). Intranasally infected piglets exhibited growth retardation, persistent fever, and higher clinical scores than controls (Fig. S1). These clinical observations, such as daily monitoring of body weight and temperature, were conducted systematically, and lung lesions were scored blindly (Fig. S1c). Gross examination revealed extensive pulmonary lesions with discoloration and consolidation (Fig. 2B). Histopathological analysis further confirmed severe interstitial pneumonia, including alveolar septal thickening, inflammatory cell infiltration, and exudation (Fig. 2C). Consistently, immunohistochemical staining showed markedly increased IL-1β expression in infected lung tissues (Fig. 2D), supporting robust inflammatory activation. To further characterize host immune responses, transcript profiling revealed strong induction of IL-1β, IL-6, and IL-8, whereas TNF-α was repressed. Negative regulators of interferon signaling (SOCS3 and USP18) were upregulated, while most ISGs (ISG15, Viperin, OAS, GBP1, and IFITM1) were suppressed, with Mx1 unchanged. Both IFN-α and IFN-β were reduced, indicating a broad suppression of type I interferon responses (Fig. 2E). Consistent patterns were observed in immortalized porcine alveolar macrophages (PAMs), where PRRSV triggered a dose-dependent cytokine surge, accompanied by concurrent repression of ISGs (Fig. 2F).

Given this paradoxical phenotype—hyperinflammation coupled with impaired antiviral defense—we next examined intracellular oxidative stress. Fluorescence imaging revealed a substantial accumulation of reactive oxygen species (ROS) in PAMs following PRRSV infection (Fig. 2G). These results suggest that PRRSV infection induces oxidative stress, which may further amplify inflammatory responses and contribute to cellular dysfunction. Based on the coexistence of inflammatory activation, ROS accumulation, and ISG suppression, we next investigated whether epitranscriptomic regulation is involved in this coordinated dysregulation. Dot blot analysis showed that PRRSV infection increased global *m6A* levels in PAMs and Marc-145 cells in a dose-dependent manner (Fig. 2H; Fig. S2a). The viral N transcript level increased progressively with each increase in MOI, confirming that the infection burden faithfully followed the intended MOI gradient (Fig. S1d), supporting a direct infection control for the *m⁶A* dot blot findings (Fig. 2H). Mechanistically, PRRSV downregulated the demethylase FTO at both mRNA and protein levels, while other *m6A* regulators were less affected (Fig. 1I through K; Fig. S2b through d). Collectively, these findings demonstrate that PRRSV infection induces a coordinated host response characterized by inflammatory activation, oxidative stress, and suppression of antiviral ISG signaling, accompanied by epitranscriptomic remodeling via FTO downregulation. This integrated dysregulation suggests a potential link between RNA modification, stress responses, and immune imbalance, prompting further investigation into the role of FTO-mediated *m6A* dynamics in regulating antiviral signaling and cellular outcomes.

### PRRSV modulates ISGs and IFN-negative regulator levels through FTO

**Fig 3:**
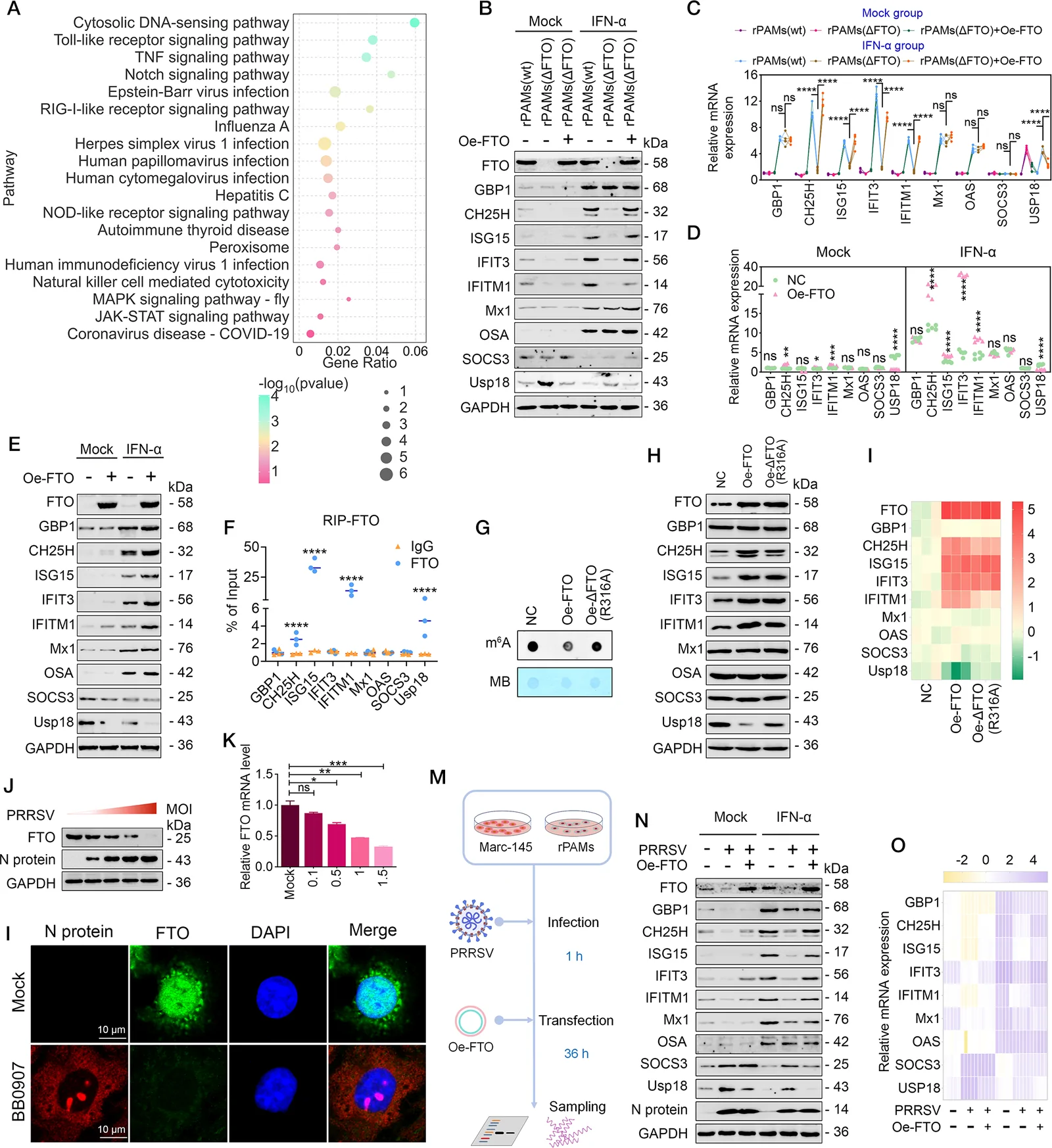
PRRSV modulates ISGs and IFN-negative regulator levels through FTO. (**A**) KEGG pathway enrichment of differentially expressed genes (DEGs) in rPAMs (control vs FTO overexpression). Dot size indicates DEG number, color denotes *P*-value, and the *x*-axis shows enrichment ratio. (**B and C**) rPAMs (wild-type or ΔFTO) transfected with Oe-FTO and treated with IFN-α at 24 h post-transfection. (**B**) Immunoblots of FTO, ISGs, and IFN-negative regulators (SOCS3 and USP18). GAPDH served as a loading control. (**C**) qRT-PCR of corresponding transcripts. (**D and E**) rPAMs were treated with or without IFN-α and transfected with Oe-NC and Oe-FTO. (**D**) ISG and negative regulator expression by qRT-PCR. (**E**) Immunoblots of FTO and target proteins. (**F**) RIP-qPCR showing FTO binding to the *m6A* regions of ISG transcripts. (**G**–**I**) rPAMs transfected with Oe-FTO or mutant Oe-ΔFTO (R316A). (**G**) Dot blot of global *m6A* levels (MB as control). (**H**) Immunoblots of FTO and ISGs/negative regulators. (**I**) Heatmap of FTO and ISG transcript levels. (**J and K**) rPAMs infected with PRRSV at MOIs 0.1–1.5 for 36 h. (**J**) Immunoblots of PRRSV-N and FTO. (**K**) qRT-PCR of FTO mRNA. (**L**) Confocal microscopy of FTO expression in Marc-145 cells ± PRRSV (BB0907) infection (scale bar = 10 µm). (**M**–**O**) rPAMs and Marc-145 cells transfected with Oe-FTO post-PRRSV infection. (**M**) Treatment scheme. (**N**) Immunoblots and (**O**) qRT-PCR of ISGs/negative regulators. (**C and D**) *n* = 5 biologically independent samples; others, *n* = 3. *P ≤ 0.05, **P ≤ 0.01, ***P ≤ 0.001, ****P ≤ 0.0001. ns = not significant.

Building on the observation shown in Fig. 2 that PRRSV downregulates FTO and increases cellular *m6A*, we performed RNA-seq following FTO overexpression. Differential analyses identified 77 genes with altered expression (Fig. S3). Kyoto Encyclopedia of Genes and Genomes (KEGG) enrichment revealed significant clustering in innate immune and interferon-related pathways, including cytosolic DNA-sensing, Toll-like receptor, RIG-I-like receptor, JAK-STAT, and TNF signaling, as well as multiple viral-infection pathways (Fig. 3A). These transcriptomic signatures suggested a functional connection between FTO and the IFN-ISG axis, prompting targeted validation.

In the FTO-knockout (ΔFTO) immortalized porcine alveolar macrophage cell line (rPAMs), expression of ISGs, such as CH25H, ISG15, IFIT3, and IFITM1, was markedly reduced, whereas the IFN inhibitory factor SOCS3 was strongly upregulated. Reintroduction of FTO into ΔFTO cells restored ISG expression and reduced SOCS3 levels (Fig. 3B and C; Fig. S4), which consistently aligned with results observed in Marc-145 cells (Fig. S4 and S5). Conversely, FTO overexpression (Oe-FTO) in wild-type cells enhanced ISG expression and decreased SOCS3 levels, opposite to the phenotype of FTO-deficient cells (Fig. 3D and E; Fig. S7 and S9). RIP-qPCR demonstrated that FTO directly bound to ISG transcripts, including CH25H, ISG15, USP18, and IFITM1 (Fig. 3F; Fig. S10a). To explore whether this regulation required catalytic activity, we utilized a well-characterized demethylase-deficient FTO mutant (R316A), a residue previously established as essential for FTO’s enzymatic activity (19). A dot blot revealed that wild-type FTO reduced global *m6A* levels, whereas the R316A mutant did not (Fig. 3G; Fig. S10b). Accordingly, in normal wild-type cells, FTO increased ISG expression and induced SOCS3, whereas the R316A mutant failed to produce these effects (Fig. 3H and I; Fig. S11 and S13). This suggests that FTO’s catalytic activity is required for its immune-promoting function.

To further elucidate the role of FTO in PRRSV-mediated immune regulation, we examined whether the virus modulates ISG expression through FTO and whether FTO expression and localization change in a dose-dependent manner upon infection. FTO protein and mRNA levels were reduced in a dose-dependent manner (Fig. 3J and K; Fig. S14), and confocal microscopy confirmed decreased nuclear localization upon infection (Fig. 3L). Rescue experiments showed that reintroducing FTO into PRRSV-infected cells restored ISG expression (CH25H, ISG15, IFIT3, and IFITM1) and suppressed SOCS3 induction (Fig. 2M through O; Fig. S15 to S17). Together, these findings establish that FTO is a crucial regulator of both ISG and IFN-negative gene expression, a role that requires its *m6A* demethylase function. PRRSV further suppresses FTO expression and nuclear localization, thereby dampening ISG responses, an effect that is reversible upon FTO restoration. Thus, PRRSV-mediated downregulation and nuclear exclusion of FTO are key mechanisms by which the virus dampens host ISG responses and facilitates immune evasion.

### PRRSV-induced *m6A* methylation changes and FTO-mediated regulation

**Fig 4:**
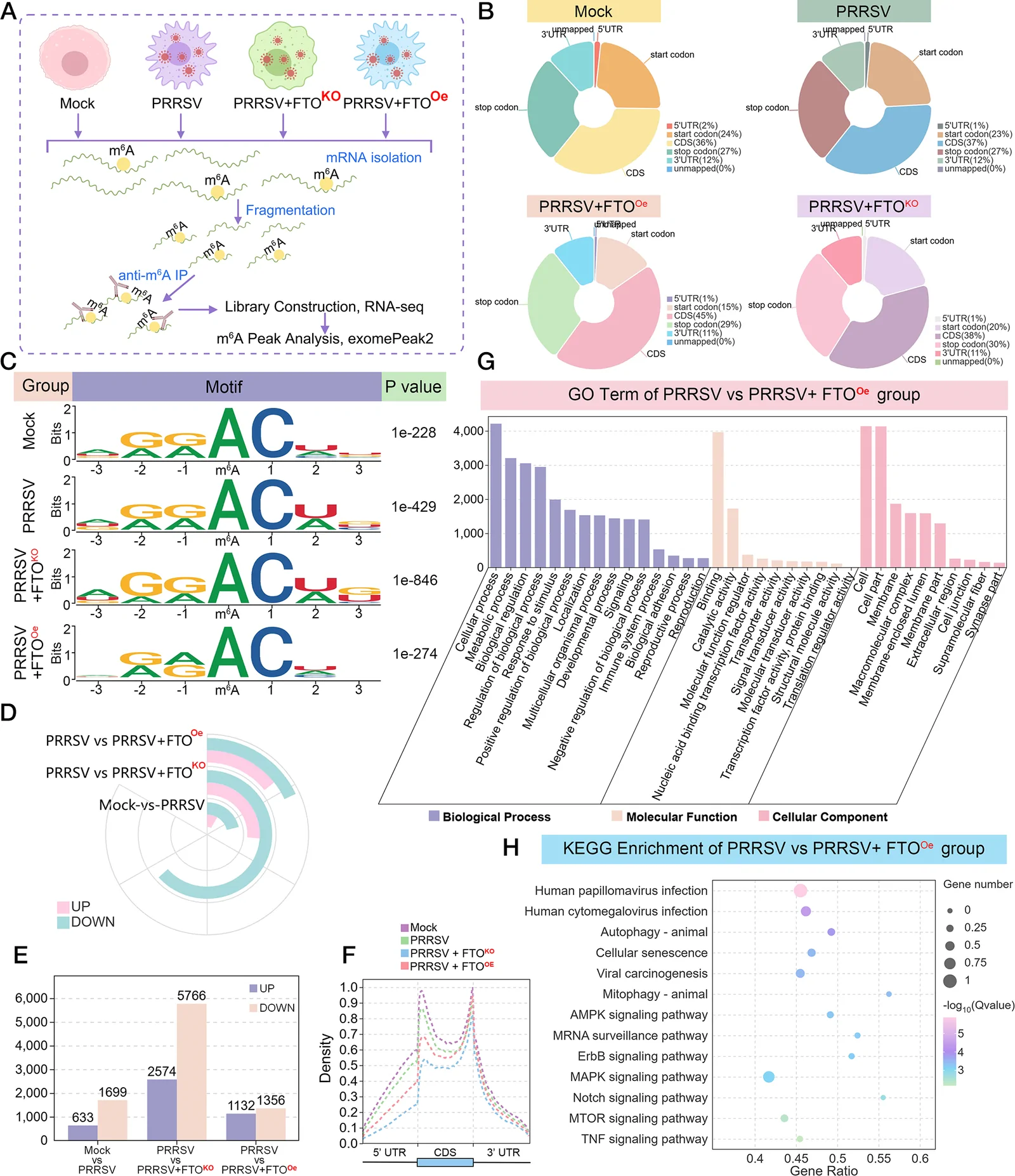
PRRSV-induced *m6A* methylation changes and FTO-mediated regulation. (**A**) Workflow of *m6A* RNA immunoprecipitation sequencing (MeRIP-seq). (**B**) Genomic distribution of m6A peaks across 5′UTRs, CDS, stop codons, and 3′UTRs. (**C**) Consensus motif analysis showing enrichment of the “RRACH” motif (R = G/A; H = A/C/U) in all groups, with *P*-values indicating significance. (**D**) Circular bar plots of up- and downregulated *m6A* peaks. (**E**) Quantification of hyper- and hypomethylated peaks. (**F**) Density distribution of *m6A* peaks along transcripts (5′UTR, CDS, and 3′UTR). (**G**) GO enrichment of differentially methylated genes between PRRSV and PRRSV + FTO^Oe^ groups, categorized into biological process (blue), molecular function (purple), and cellular component (pink). (**H**) KEGG pathway enrichment of differentially methylated transcripts between PRRSV and PRRSV + FTO^Oe^ groups. Dot size represents gene number; color indicates adjusted *P*-value.

To explore how PRRSV reshapes the host epitranscriptome through FTO, we performed transcriptome-wide *m6A-*seq in four groups: Mock, PRRSV, PRRSV with FTO knockout (PRRSV + FTO^KO^), and PRRSV with FTO overexpression (PRRSV + FTO^Oe^) (Fig. 4A). *m6A* peaks showed canonical enrichment in coding regions and near stop codons across all groups (Fig. 4B), and motif analysis confirmed the conserved “RRACH” motif (Fig. 4C), validating the data set’s quality. Global comparisons revealed that PRRSV infection markedly increased the number of *m6A* peaks (Fig. 4D and E). FTO knockout further enhanced hypermethylation, whereas FTO overexpression led to a net reduction, supporting FTO as the principal enzymatic regulator of PRRSV-induced *m6A* dynamics. Metagene analysis showed that PRRSV infection increased *m6A* density around the CDS and 3′UTR, with opposing effects on FTO overexpression and loss (Fig. 4F). Functional enrichment analysis demonstrated that these *m6A* changes were non-random and were closely linked to host immunity. GO analysis revealed strong associations with interferon responses, immune regulation, and inflammatory signaling (Fig. 4G; Fig. S18a). KEGG analysis further highlighted infection- and immunity-related pathways, including RIG-I-like receptor signaling, cytosolic DNA sensing, and NOD-like receptor signaling, as well as cytokine networks such as JAK-STAT, TNF, and MAPK cascades (Fig. 4H; Fig. S18b).

Furthermore, reads mapped to the viral genome were extracted and analyzed for *m6A* peak distribution and differential methylation. The results showed that no reproducible, statistically significant *m6A* peaks were detected on PRRSV genomic or subgenomic RNAs across the four experimental groups (Mock, PRRSV, PRRSV + FTO^KO^, and PRRSV + FTO^Oe^). Viral RNA levels (measured by RNA-seq) increased as expected after infection, but the lack of detectable *m6A* peaks indicates that under certain conditions, PRRSV RNA is either not significantly methylated or the methylation level is below the detection limit for *m6A*-seq. These findings demonstrate that altered methylation of viral RNA is unlikely to be a major contributor to the observed changes in PRRSV replication in our system. Instead, our findings support a model in which PRRSV primarily manipulates host *m6A* machinery (via FTO suppression) to reprogram the expression of host immune transcripts, particularly those involved in JAK-STAT signaling. Together, these findings demonstrate that PRRSV suppresses FTO to reprogram *m6A* methylation on immune- and interferon-related transcripts, thereby linking epitranscriptomic remodeling to viral immune evasion.

### FTO regulates ISG expression through the phosphorylation of STAT2 and STAT3

**Fig 5:**
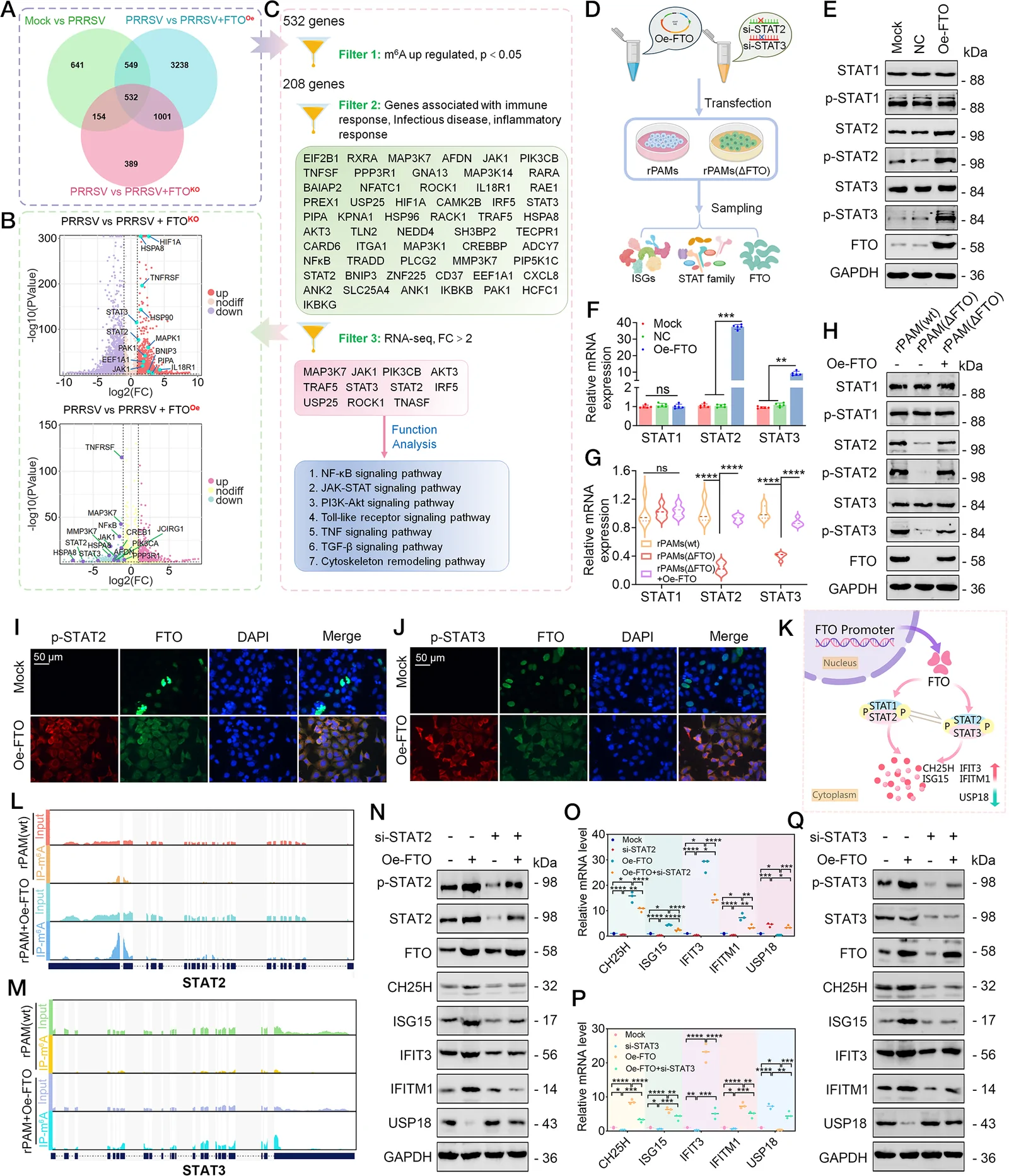
FTO regulates ISG expression through the phosphorylation of STAT2 and STAT3**.** (**A**) Venn diagram showing overlap of differentially methylated *m6A* peaks among comparisons (Mock vs PRRSV, PRRSV vs PRRSV + FTO^Oe^, and PRRSV vs PRRSV + FTO^KO^). (**B**) Volcano plots of DEGs between PRRSV vs PRRSV + FTO^KO^ (top) and PRRSV vs PRRSV + FTO^Oe^ (bottom). (**C**) Pipeline for selecting core genes based on integrated *m6A* and transcriptome data. (**D**) Experimental design: rPAMs (WT or ΔFTO) transfected with Oe-FTO, si-STAT2, or si-STAT3, followed by qRT-PCR and immunoblotting at 36 h. (**E and F**) rPAMs transfected with Oe-FTO or Oe-NC: (**E**) immunoblots of STATs and p-STATs; (**F**) qRT-PCR of STAT mRNAs. (**G and H**) WT and ΔFTO rPAMs ±Oe-FTO: (**G**) qRT-PCR of STATs; (**H**) immunoblots of STATs and p-STATs. (**I and J**) IFA of p-STAT2 (**I**) and p-STAT3 (**J**) in Marc-145 cells ± Oe-FTO or vector control (Mock) (scale bar, 50 μm). (**K**) Proposed model: FTO regulates ISG expression via STAT2/3 signaling. (**L and M**) IGV tracks showing *m6A* peak distribution on STAT2 (**L**) and STAT3 (**M**) transcripts in Oe-FTO vs control rPAMs. (N–Q) rPAMs transfected with Oe-FTO ± si-STAT2 (**N and O**) or si-STAT3 (**P and Q**): (**N and Q**) immunoblots of ISGs and IFN negative regulators (SOCS3 and USP18); (**O and P**) qRT-PCR of corresponding transcripts. Data represent three independent experiments; panels **F** and **G**, *n* = 5 biologically independent samples. *P ≤ 0.05, **P ≤ 0.01, ***P ≤ 0.001, ****P ≤ 0.0001; ns, not significant.

STAT transcription factors are critical for antiviral IFN signaling (20, 21). FTO has previously been linked to STAT3 activation, while its role in immune regulation remains underexplored (22). To identify downstream effectors of FTO-mediated immune regulation, we integrated *m6A-*seq and RNA-seq data sets. Across the four treatment groups, 532 genes exhibited significant *m6A* alterations (Fig. 5A). Stepwise filtering for immune- and inflammation-related pathways yielded 208 candidates, which were further narrowed to 11 genes by transcriptomic integration (Fig. 5B and C). These were enriched in antiviral and inflammatory cascades, prominently within the JAK-STAT pathway, with STAT-family members and MAP3K7 emerging as central nodes.

We next tested whether FTO modulates STAT signaling (Fig. 5D). Overexpression of FTO selectively inhibited STAT2 and STAT3 phosphorylation without affecting STAT1, whereas FTO knockout enhanced STAT2/3 phosphorylation, which was reversed by FTO reintroduction (Fig. 4E through H; Fig. S19 and S20a through g). Consistent results were obtained in Marc-145 cells (Fig. S1). Immunofluorescence and quantification of mean fluorescence intensity (MFI) further confirmed reduced levels of p-STAT2 and p-STAT3 in FTO-overexpressing cells (Fig. 5J; Fig. S20h through k). Integrative Genomics Viewer (IGV) tracks revealed distinct *m6A* peaks on STAT2 and STAT3 transcripts that were markedly reduced following FTO overexpression (Fig. 5L and M; Fig. S24), indicating direct targets of FTO-mediated demethylation. Functionally, knockdown of STAT2 or STAT3 abrogated the ability of FTO to repress ISGs (CH25H, ISG15, IFIT3, and IFITM1) and to induce the IFN-inhibitory factor USP18 (Fig. 4N through Q; Fig. S25 and S26), suggesting that the effects of FTO on ISG expression and IFN-negative regulators (USP18) are strictly dependent on STAT2 and STAT3. Similar effects were observed in Marc-145 cells (Fig. S27 to S29). While siRNA-mediated knockdown was used here to validate STAT2 and STAT3, future studies employing genetic knockout models could provide further confirmation of these interactions. Together, these results demonstrate that FTO directly demethylates STAT2 and STAT3 transcripts, thereby inhibiting their phosphorylation and the downstream induction of ISGs. This positions the JAK-STAT axis as a key pathway that FTO exploits to mediate PRRSV immune evasion (Fig. 5K). Together, these data establish a causal chain in which FTO directly demethylates STAT2 and STAT3 mRNAs, thereby promoting their translation and/or stability, enhancing STAT2/3 phosphorylation and downstream ISG induction. PRRSV suppresses FTO, leading to hypermethylation of these transcripts, reduced STAT2/3 activation, and impaired antiviral immunity.

### PRRSV nsp11 and its critical domain inhibit FTO expression

**Fig 6:**
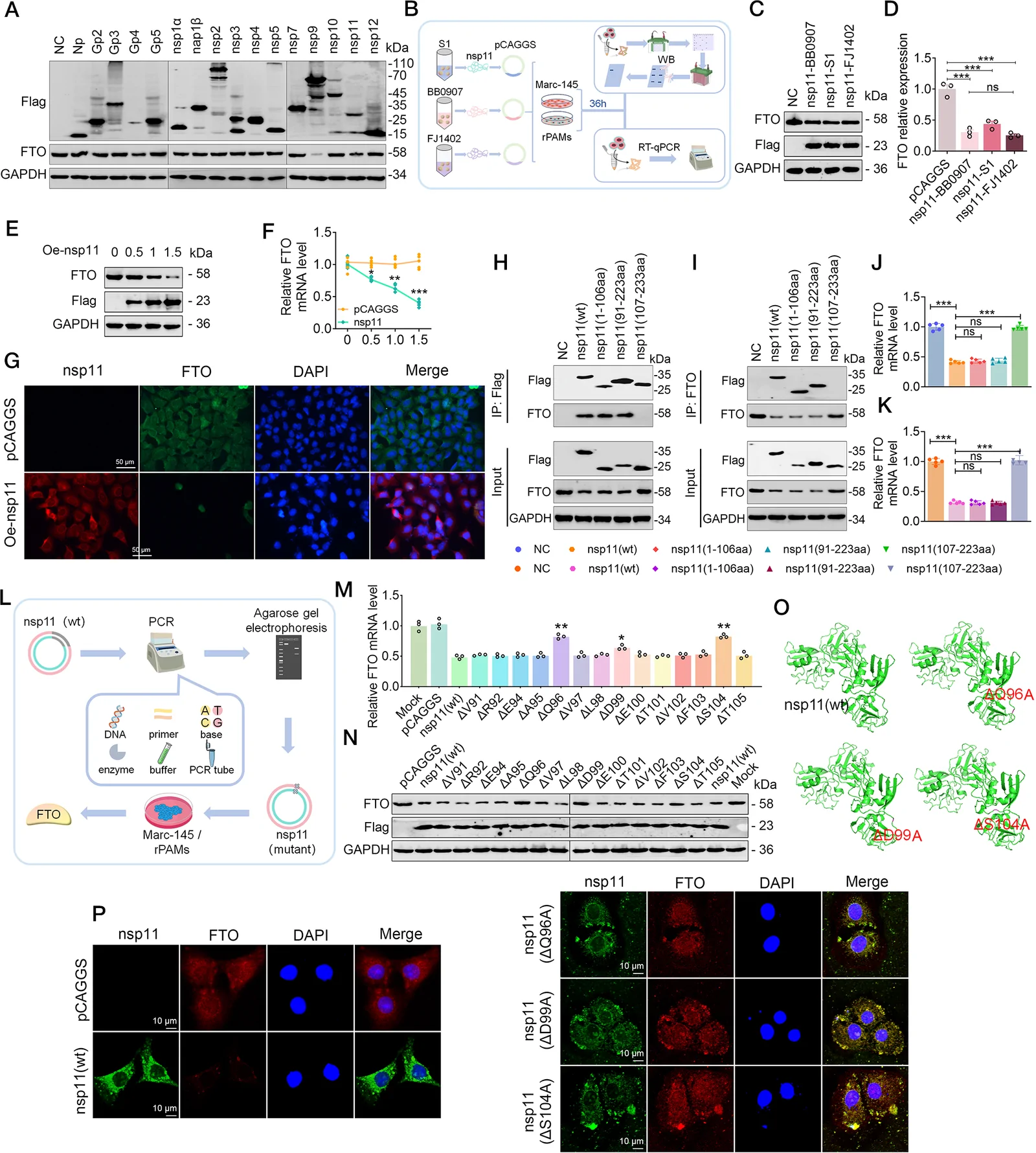
PRRSV nsp11 and its critical domain inhibit FTO expression**.** (**A**) Immunoblots of FTO in rPAMs transfected with plasmids encoding individual PRRSV proteins (36 h). (**B**) Experimental design for Marc-145 and rPAMs transfected with nsp11 plasmids from strains BB0907, S1, or FJ1402. (**C and D**) FTO expression in rPAMs assessed by (**C**) immunoblots and (**D**) qRT-PCR. (**E and F**) Dose-dependent effects of nsp11 plasmid (0.5, 1.0, and 1.5 μg) on FTO levels in rPAMs, measured by (**E**) immunoblots and (**F**) qRT-PCR. (**G**) IFA of FTO expression in rPAMs ± nsp11 overexpression (scale bar, 50 μm). (H–K) rPAMs transfected with nsp11 (wt) or truncated mutants. (**H and I**) Immunoprecipitation with Flag (**H**) or FTO (**I**) antibodies followed by immunoblotting. (**J and K**) qRT-PCR of FTO mRNA from the same experiments. (**L**) Workflow for generating glycine-to-alanine nsp11 mutant plasmids. (**M and N**) rPAMs transfected with nsp11 (wt) or site-directed mutants: (**M**) qRT-PCR of FTO mRNA; (**N**) immunoblots of FTO and nsp11. (**O**) Predicted 3D structures of nsp11 (wt) and mutants (Q96A, D99A, and S104A). (**P**) rPAMs transfected with nsp11 (wt) or site-directed mutants (Q96A, D99A, and S104A). Confocal fluorescent image of nsp11 and FTO (scale bar, 10 μm). Data in F, J, and K represent *n* = 5 biologically independent samples; the other experiments were repeated three times. *P ≤ 0.05, **P ≤ 0.01, ***P ≤ 0.001, ****P ≤ 0.0001. ns = not significant.

Having established that FTO suppresses interferon signaling via the JAK-STAT axis (Fig. 5), we next investigated how PRRSV nsp11 modulates FTO. Since viral nsps are critical for PRRSV infection (23), we screened viral proteins and identified several candidates, including GP4, nsp9, and nsp11, that reduced FTO expression (Fig. 6A; Fig. S30a). Crucially, we previously demonstrated that PRRSV nsp9 can decrease FTO binding, thereby increasing *m6A*-modified motifs in IL-13 secretion (24), but nsp11 was prioritized for its role in the specific transcriptional mechanism. Unlike nsp9, nsp11 contains a NendoU endoribonuclease domain conserved among arteriviruses and homologous to nsp15 in coronaviruses (including SARS-CoV and MERS-CoV) (25), suggesting a potentially conserved immune-evasion mechanism worth exploring. Therefore, we focused on its contribution to host epitranscriptomic regulation. Overexpression of nsp11 from representative PRRSV strains (BB0907, S1, and FJ1402) led to a dose-dependent reduction of FTO protein and mRNA levels in Marc-145 cells and rPAMs (Fig. 5B through F; Fig. S30b through e and S31a through h). Immunofluorescence and quantification of mean fluorescence intensity (MFI) confirmed a decrease in FTO abundance and impaired nuclear accumulation (Fig. 6G; Fig. S31i and S31j), indicating that nsp11 affects both expression and intracellular distribution of FTO. To define the structural basis of this interaction, truncated nsp11 variants (Fig. S32a) were tested by Co-IP, which mapped the FTO-binding region to residues 91–106 (Fig. 5H through K; Fig. S32b through e). Within this region, site-directed mutagenesis identified Q96, D99, and S104 as critical residues required for FTO suppression (Fig. 6L). Structural modeling confirmed that these substitutions did not perturb the global nsp11 fold (Fig. 6M and N; Fig. S33 and S34), supporting their role as functional rather than structural determinants (Fig. 6O; Fig. S35).

To further investigate whether nsp11 residues affect FTO’s subcellular distribution, we utilized confocal microscopy. Overexpressing wild-type nsp11 substantially decreased FTO levels and nuclear localization. Conversely, mutants Q96A, D99A, and S104A were less effective (Fig. 6P), suggesting that these residues are essential for nsp11’s complete suppression of FTO expression and localization. Together, these results demonstrate that PRRSV nsp11 directly targets FTO through a defined 91–106 aa motif, reducing its expression and nuclear localization. Taken together with Fig. 5, this establishes a mechanistic axis in which nsp11 attenuates FTO, thereby weakening JAK-STAT-mediated interferon signaling, identifying nsp11 as a central viral effector that rewires host epitranscriptomic regulation for immune evasion.

### PRRSV nsp11 regulates FTO expression through STAT5

**Fig 7:**
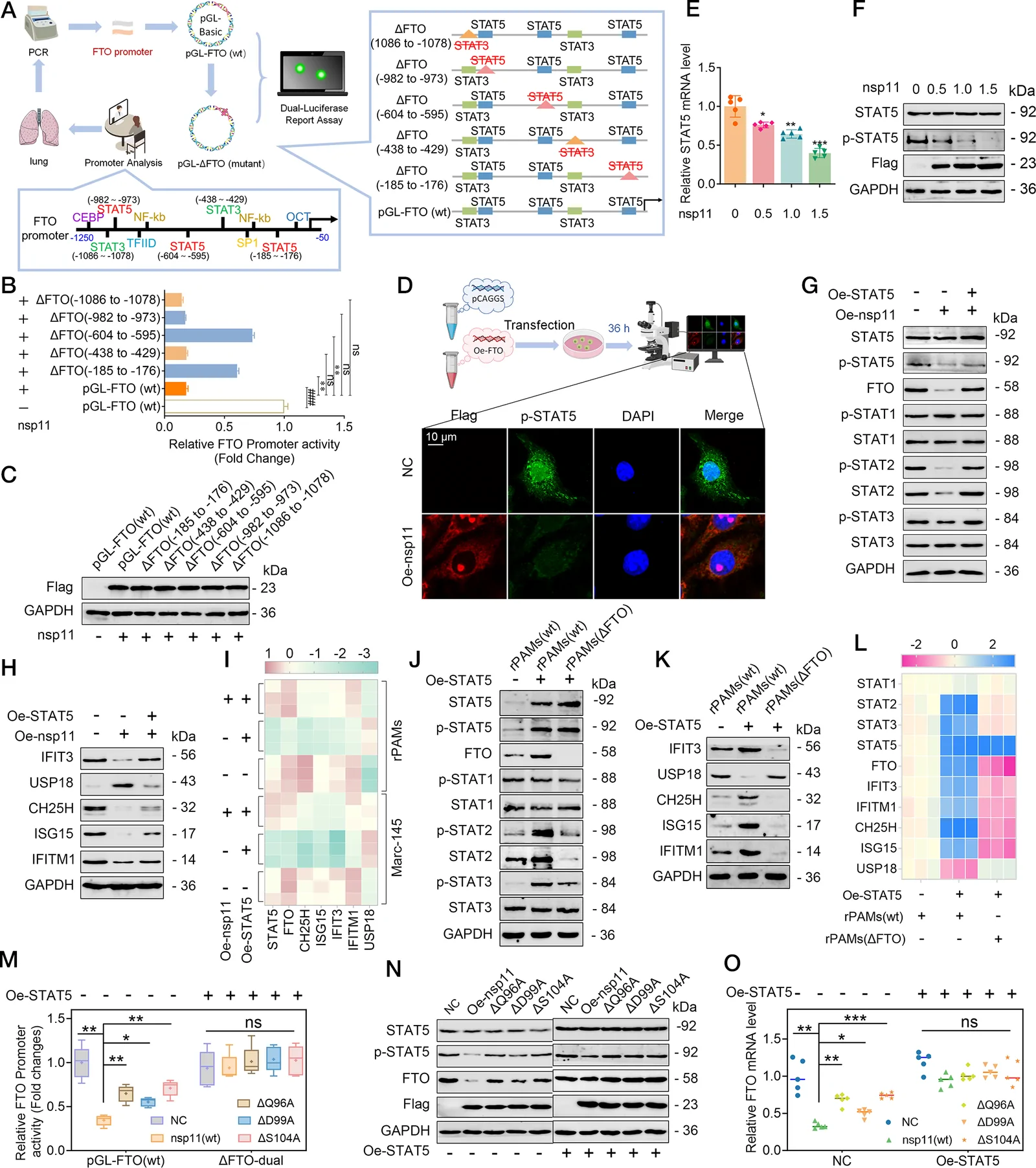
PRRSV nsp11 regulates FTO through STAT5. (**A**) Bioinformatic analysis of the FTO promoter identified multiple STAT3/STAT5-binding sites, which were mutated to generate FTO promoter mutants. (**B and C**) rPAMs transfected with mutant FTO promoter or pGL3 control and Oe-nsp11: (**B**) dual-luciferase reporter assay of promoter activity; (**C**) immunoblot of nsp11. (**D**) Confocal microscopy of p-STAT5 in Marc-145 cells ± Oe-nsp11 (scale bar, 10 μm). (**E and F**) rPAMs transfected with increasing doses of nsp11 plasmid (0.5–1.5 μg, 36 h): (**E**) qRT-PCR of STAT5 mRNA; (**F**) immunoblot of STAT5/p-STAT5. (**G**–**I**) rPAMs transfected with Oe-nsp11 ± Oe-STAT5: (**G**) immunoblots of FTO, STATs/p-STATs; (**H**) ISG and IFN negative regulator proteins; (**I**) qRT-PCR of transcripts in Marc-145 and rPAMs. (**J**–**L**) WT and ΔFTO rPAMs ±Oe-STAT5: (**J**) immunoblots of FTO, STATs/p-STATs; (**K**) ISGs and IFN negative regulators; (**L**) qRT-PCR heatmap of transcripts. (**M**–**O**) rPAMs transfected with WT or mutant nsp11 (Q96A, D99A, and S104A) and Oe-STAT5 plus luciferase reporter: (**M**) dual-luciferase assay of FTO promoter; (**N**) immunoblots of FTO, STAT5, p-STAT5, and nsp11. (**O**) qRT-PCR of mRNA expression. Data are from five (**E and O**) or three (others) independent experiments. *P ≤ 0.05, **P ≤ 0.01, ***P ≤ 0.001, ****P ≤ 0.0001. ns = not significant.

Having established that nsp11 targets FTO through specific residues (Fig. 6), we next investigated whether this regulation occurred at the transcriptional level. Promoter analysis revealed multiple transcription factor motifs within the FTO promoter, with STAT3/5 sites being most frequent (Fig. 7A; Fig. S36). Dual-luciferase assays using site-specific mutants demonstrated that disruption of two STAT5-binding sites (–604 to –595 and –185 to –176) abolished nsp11-mediated repression of FTO promoter activity (Fig. 7B and C; Fig. S37), implicating STAT5 as a key mediator. Consistently, nsp11 inhibited STAT5 phosphorylation in a dose-dependent manner and prevented its nuclear localization (Fig. 6D through F; Fig. S38 and S39). Co-expression assays showed that nsp11 suppressed p-STAT5, FTO, and downstream STAT2/3 activation, accompanied by reduced ISG expression (ISG15, CH25H, IFIT3, and IFITM1) and elevated USP18. Importantly, STAT5 overexpression largely restored these effects at both the protein and transcript levels (Fig. 6G through I; Fig. S40), a result validated in Marc-145 cells (Fig. S41 and S42). To determine the dependence on FTO, we examined FTO-deficient rPAMs. In these cells, STAT5 overexpression failed to fully rescue ISG expression and STAT activation, indicating that STAT5-mediated antiviral signaling is partly dependent on FTO (Fig. 6J through L; Fig. S43). Similar results were obtained in Marc-145 cells with FTO knockdown (Fig. S44 and S45).

Finally, promoter activity assays with nsp11 mutants (Q96A, D99A, and S104A) demonstrated that disruption of these residues attenuated nsp11-mediated repression of FTO promoter activity. Co-expression with STAT5 confirmed that these residues are essential for nsp11’s ability to suppress STAT5 phosphorylation and FTO transcription (Fig. 6M through O; Fig. S46 and S47), which was also validated in Marc-145 cells (Fig. S48 and S49). Together, these findings establish that PRRSV nsp11 regulates FTO expression through a STAT5-dependent pathway, with Q96, D99, and S104 serving as critical determinants of this host-virus regulatory axis.

To further explore whether nsp11 is associated with STAT5, co-immunoprecipitation assays were performed. HA-STAT5 was co-expressed with Flag-tagged nsp11, Flag-nsp11(Q96A), or Flag-nsp11(S104A). Immunoprecipitation with anti-Flag antibody revealed that HA-STAT5 was present in Flag-nsp11 immunoprecipitates, indicating that nsp11 and STAT5 are present in the same immunoprecipitated complex. Notably, both nsp11 mutants retained the ability to co-immunoprecipitate with STAT5, suggesting that the Q96A and S104A mutations do not markedly disrupt the formation of the nsp11-STAT5-containing complex. However, these mutants exhibited altered capacity to suppress STAT5 phosphorylation compared with wild-type nsp11, indicating that these residues are critical for functional regulation rather than the observed association with STAT5 (Fig. S50). We next assessed JAK1/2 expression and phosphorylation to rule out the possibility that nsp11 acts through upstream JAK kinases. Overexpression of nsp11 (wild-type or mutants) did not alter total or phosphorylated JAK1/2 levels (Fig. S51). Importantly, the endoribonuclease-defective mutants nsp11(Q96A) and nsp11(S104A), which we have shown disrupt FTO suppression, also did not cause any notable decrease in JAK1/2 transcript levels. Taken together, these data indicate that nsp11 suppresses STAT5 phosphorylation through a JAK1/2-independent mechanism, possibly involving an nsp11-STAT5-containing complex rather than altered upstream JAK1/2 signaling.

### PRRSV infectious clone suppresses FTO to coordinate ISG inhibition and oxidative stress responses

**Fig 8:**
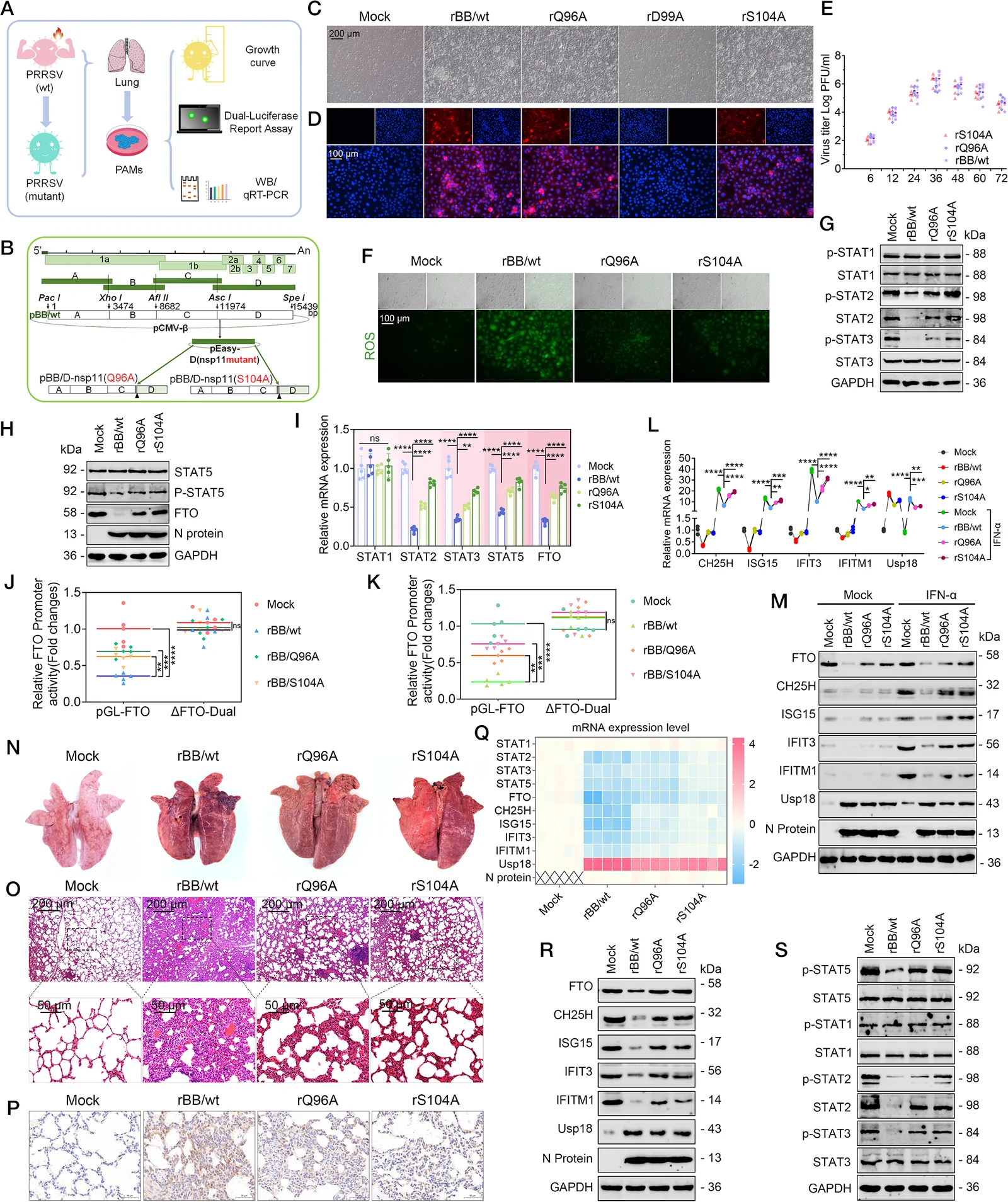
PRRSV infectious clone inhibits FTO expression and regulates ISGs**.** (**A**) Schematic showing construction and successful rescue of recombinant PRRSV mutants (rQ96A, rD99A, and rS104A), followed by the validation of viral growth kinetics prior to mechanistic analyses. (**B**) Generation of full-length nsp11-mutant infectious cDNA clones. (**C and D**) Marc-145 cells transfected with mutant clones (pBB/Q96A, pBB/D99A, and pBB/S104A) showing (**C**) cytopathic effects and (**D**) N protein expression by IFA (Scale bar, 100 µm). (**E**) Multistep growth kinetics of PRRSV mutants (MOI = 0.1) in Marc-145 cells, expressed as TCID_50_. (**F**) Intracellular reactive oxygen species (ROS) levels in rPAMs infected with rBB/wt, rQ96A, or rS104A (36 h), detected by fluorescence imaging (Scale bar, 100 µm). (**G**–**I**) rPAMs infected with rBB/wt, rQ96A, or rS104A (36 h): (**G and H**) immunoblots of FTO, STATs/p-STATs, and N protein; (**I**) qRT-PCR of FTO and STATs mRNAs. (**J and K**) FTO promoter activity in (**H**) Marc-145 cells and (**I**) rPAMs infected with recombinant viruses. (**L and M**) rPAMs infected with recombinant viruses ± IFN-α (36 h): (**L**) qRT-PCR and (**M**) immunoblots of FTO and ISGs. (**N**) Gross lung pathology in infected pigs. (**O**) H&E staining of lung sections (scale bar, 50 µm). (**P**) IHC staining of IL-1β in pig lung tissues (Scale bar, 50 µm). (**Q**) qRT-PCR of FTO, ISGs, and STATs in pig lungs. (**R and S**) Immunoblots of FTO, ISGs, STATs/p-STATs, and N protein in pig lungs. Data are mean ± SD; (**E**, **I**–**K**, and **Q**) *n* = 5; others *n* = 3. *P ≤ 0.05, **P ≤ 0.01, ***P ≤ 0.001, ****P ≤ 0.0001; ns, not significant.

To further validate the role of FTO-STAT signaling during viral infection, we generated recombinant PRRSV carrying point mutations in the glycine-rich domain of nsp11 (rQ96A, rD99A, and rS104A) using reverse genetics (Fig. 8A and B). rQ96A and rS104A were successfully rescued and displayed replication kinetics comparable to rBB/wt, whereas rD99A could not be recovered, indicating that this residue is essential for viral replication (Fig. 7C through E). Notably, intracellular reactive oxygen species (ROS) and quantification mean fluorescence intensity (MFI) levels were markedly elevated in rBB/wt-infected cells, whereas rQ96A and rS104A infections significantly attenuated ROS accumulation (Fig. 8F; Fig. S52k), suggesting that disruption of the nsp11-FTO axis alleviates PRRSV-induced oxidative stress. Consistent with this, compared with rBB/wt, infections with rQ96A and rS104A partially restored FTO expression and phosphorylation of STAT2, STAT3, and STAT5 (Fig. 7G through I; Fig. S52 to S54). Luciferase assays further confirmed that these mutants attenuated viral repression of FTO promoter activity, and this effect was abolished upon deletion of STAT5-binding motifs within the promoter (Fig. 8J and K). In PAMs, rQ96A and rS104A infection enhanced ISG expression (CH25H, ISG15, IFIT3, and IFITM1) while reducing USP18, consistent with reactivation of interferon signaling (Fig. 8L and M; Fig. S55). These results indicate that disruption of nsp11-mediated FTO suppression restores antiviral signaling while alleviating stress-associated responses. *In vivo* piglet experiments further supported these findings. rBB/wt infection caused severe pulmonary lesions with extensive inflammatory infiltration, accompanied by significant body weight loss, persistent fever, and higher pathological scores. In contrast, rQ96A and rS104A induced only mild interstitial inflammation with relatively stable body weight, normal temperature, and lower pathological scores (Fig. 8N and O; Fig. S56). IHC analysis revealed markedly higher IL-1β expression in rBB/wt-infected lungs compared with mutant groups (Fig. 8P). At both transcript and protein levels, rQ96A and rS104A restored FTO and ISG expression and increased STAT phosphorylation relative to rBB/wt (Fig. 7Q through S; Fig. S57). Collectively, these results demonstrate that PRRSV nsp11 suppresses FTO to coordinate inhibition of interferon signaling and induction of oxidative stress, thereby promoting immune-stress dysregulation. Mutations at Q96 and S104 disrupt this regulatory axis, linking specific viral residues to host epitranscriptomic remodeling and pathological outcomes.

## DISCUSSION

The interplay between RNA viruses and the host epitranscriptome represents a burgeoning frontier in the study of host-pathogen interactions. While *m6A* plays a critical role in modulating a cell’s antiviral defenses, RNA viruses have evolved sophisticated mechanisms to subvert host gene expression and cellular functions, thereby enhancing their replication (8, 26). Recently, it has been demonstrated that *m6A* modification enables viral RNA to evade recognition by the RNA sensor RIG-I (26), underscoring its crucial role in immune defense. Although the roles of *m6A* writers and readers in antiviral defense are increasingly recognized (8, 27, 28), how RNA viruses manipulate *m6A* erasers to their advantage remains poorly understood. Our study provides a novel insight into an immune evasion strategy employed by PRRSV, revealing a complete signaling axis from a specific viral protein to the targeted suppression of a host demethylase, thereby silencing antiviral immunity at the epitranscriptomic level. Notably, PRRSV nsp11 downregulates FTO expression and its nuclear localization, causing global *m⁶A* hypermethylation. PRRSV infects both PAMs and MARC-145 cells, establishing an evolutionarily relevant model for investigating RNA virus-host interactions across species. The consistent results in both systems underscore a conserved, RNA virus-specific strategy that transcends species barriers. This design not only rules out cell line-specific artifacts but also provides insight into how RNA viruses adapt their epitranscriptomic regulation across diverse mammalian hosts. Although siRNA approaches were complemented by CRISPR/Cas9 knockouts of key targets, such as FTO, using additional knockout cell lines for downstream effectors, such as STAT2/3, may further strengthen the proposed axis. Additionally, we report that PRRSV infection employs a dual-pathogenicity strategy that triggers a damaging cytokine storm and broadly induces the host *m6A* epitranscriptome. This coordinated silencing of erasers disables antiviral RNA regulation, revealing the *m6A* pathway as a critical battlefield and a promising therapeutic target.

Recent research has shown that *m6A* methylation on viral RNA can play either a proviral or an antiviral role, with effects that are specific to both the viral species and the infected cell type (13, 14). The central finding of this work is the identification of *m6A* erasers (FTO) as a pivotal proviral factor co-opted by an RNA virus. In contrast to the conventional view that *m6A* erasers are negative regulators of viral replication (29), we demonstrate that PRRSV actively suppresses FTO to establish a cellular environment conducive to its persistence. This FTO downregulation is not a passive bystander effect but is directly orchestrated by the viral nsp11 protein. The viral nsp11 protein, which contains a NendoU endoribonuclease domain conserved among arteriviruses (30) and is homologous to the Nsp15 protein in coronaviruses (including SARS-CoV and MERS-CoV) (25), functions as a multifunctional hub linking viral replication to host RNA modification reprogramming. Notably, we identify a glycine-rich domain (aa 91–106) in nsp11, with residues Q96 and S104 critical for FTO suppression. The fact that recombinant viruses with mutations at these sites (rQ96A and rS104A) are attenuated *in vivo* highlights the biological importance of this interaction and further confirms nsp11’s FTO-suppressive role as an effective virulence factor. This represents a novel, non-canonical function for nsp11, expanding its role beyond viral RNA processing to include direct reprogramming of the host epitranscriptome. Furthermore, our confocal analysis showed that nsp11 suppresses and excludes FTO from the nucleus, a requirement that needs an intact glycine-rich domain. Mutations at Q96, D99, and S104 partially restored FTO’s nuclear localization. We further note that reintroducing FTO or expressing nsp11 mutants (Q96A and S104A) restores STAT2/3 phosphorylation and ISG expression. This spatially sequesters FTO, enhancing silencing of its targets in interferon signaling. It is critical to note that mechanistic studies of FTO suppression have mainly focused on PRRSV, with the nsp11 NendoU domain conserved among arteriviruses and nidoviruses. Further research is warranted to extend the findings to other RNA viruses, especially those with homologous endoribonucleases, which also target FTO or other *m6A* erasers for immune evasion. Although *m6A* modification of viral RNA has been reported to regulate RNA virus replication (26, 31), we did not find reproducible *m6A* peaks on PRRSV RNA in our *m6A*-seq data. This indicates that under certain conditions, direct methylation of PRRSV is not a major mechanism. Instead, PRRSV mainly uses host *m6A* regulation, by suppressing FTO, to alter the host epitranscriptome and reduce innate immunity. However, low-level or transient viral RNA methylation below our detection limit, or different viral strains or infection times, might yield different results.

Mechanistically, we outline how FTO suppression culminates in impaired interferon signaling. Our integrated *m6A*-seq and RNA-seq analysis revealed that the JAK-STAT pathway is a primary target. Specifically, among the hypermethylated transcripts, STAT2 and STAT3 are functionally validated targets. Increased m⁶A on these mRNAs correlates with decreased translation efficiency and reduced protein phosphorylation. FTO, through its demethylase activity, directly targets and positively regulates the phosphorylation of STAT2 and STAT3, but not STAT1. This selective regulation provides a mechanistic explanation for the “differential silencing” strategy observed during RNA virus infection, in which specific branches of the interferon response are selectively muted to balance immune suppression with cellular viability. Importantly, decreased p-STAT2/3 directly affects the transcription of a specific subset of ISGs, while STAT1-dependent pathways remain partially intact, a “differential silencing” strategy. FTO’s regulation of STAT3 signaling is cell type-dependent (32, 33); specifically, in adipocytes, FTO promotes STAT3 activation by stabilizing JAK2 mRNA, thereby enhancing STAT3 phosphorylation at tyrosine 705 (34). However, our findings of the STAT isoform-specific regulation reveal a previously unknown layer of control within the JAK-STAT cascade. By erasing *m6A* marks on STAT2 and STAT3 mRNAs, FTO facilitates efficient translation and/or protein stability, ensuring robust signaling flux for ISG induction. Thus, the nsp11-STAT5-FTO axis operates through a direct epitranscriptomic mechanism by decreasing FTO, which increases *m⁶A* on STAT2/3 mRNAs, suppressing their translation and phosphorylation, which ultimately cripples the IFN-ISG response. PRRSV, by suppressing FTO, cripples this specific arm of the interferon response, effectively muting the expression of a crucial arsenal of antiviral effectors while leaving other STAT1-dependent signals partially intact. This “differential silencing” enables the virus to suppress immunity without causing widespread, potentially harmful, cellular shutdown. Finally, it is critical to note that reintroducing FTO or expressing nsp11 mutants (Q96A and S104A) restores STAT2/3 phosphorylation and ISG expression, confirming the causal relationship.

Our findings on the nsp11-STAT5-FTO-STAT axis reveal a unique immune evasion mechanism within the *m6A* research. While earlier studies focused on viruses exploiting *m6A* writers (METTL3/METTL14) and readers (YTHDF) to enhance replication or suppress immune responses (8, 13, 26), our study aligns with a few studies demonstrating that a virus can evade immunity by targeting an *m6A* eraser (FTO) (35). PRRSV nsp11 downregulates FTO expression and its nuclear localization, leading to global *m⁶A* hypermethylation. This approach broadly reprograms the host epitranscriptome, leading to a hypermethylation state that interferes with JAK-STAT signaling. It shows that viral regulation of the epitranscriptome involves not only adding or reading *m6A* marks but also actively removing components that reverse them. The nsp11-STAT5-FTO axis illustrates a novel viral mechanism strategy.

Furthermore, we unravel the upstream event linking the viral trigger to FTO suppression. We identified STAT5 as the host transcription factor that nsp11 manipulates to repress FTO transcription. Notably, nsp11 did not affect upstream JAK1/2 kinases but directly interacted with STAT5, suggesting a non-canonical, JAK-independent mode of STAT5 inhibition that may involve blocking STAT5 nuclear translocation or DNA binding. This mechanism adds to the growing appreciation of viral proteins directly targeting STAT transcription factors to evade innate immunity. The demonstration that nsp11 inhibits STAT5 phosphorylation, preventing its nuclear translocation and binding to the FTO promoter, completes the mechanistic cycle, showing that PRRSV nsp11 inactivates STAT5, leading to FTO downregulation and STAT2/3 hypophosphorylation, and thereby suppressing ISGs. We uncover a highly conserved RNA virus-STAT5-FTO-STAT2/3-ISG multi-tiered inhibitory signaling axis that mediates a hierarchical, amplifiable immune-silencing network, illustrating a universal mechanism by which RNA viruses precisely exploit the host epitranscriptome to suppress antiviral signaling. This nsp11-STAT5-FTO-STAT2/3 axis represents a model of remarkable efficiency, in which a single viral protein orchestrates a multi-step hijacking of host transcriptional and post-transcriptional regulatory networks. Although our data indicate that nsp11 does not transcriptionally upregulate known STAT5-targeting phosphatases or inhibit JAK1/2 activity, we cannot rule out the involvement of phosphatases regulated post-translationally. For instance, nsp11 might recruit phosphatases to STAT5 via its glycine-rich domain, with residues Q96 and S104 playing critical roles in this recruitment. Alternatively, nsp11 could induce a conformational change in STAT5 that renders it a better substrate for constitutively active phosphatases. Additionally, it is critical to note that the effect of nsp11 on the STAT5-FTO axis is unlikely to be mediated by JAK1/2 mRNA destabilization. Future studies using phosphatase inhibitors to detect nsp11-phosphatase-STAT5 ternary complexes, and *in vitro* dephosphorylation assays with recombinant proteins, will be required to directly test these possibilities.

The potential implications of this work are 3-fold. First, it advances our understanding of RNA virus pathogenesis by providing a molecular explanation for the clinical paradox of hyperinflammation concurrent with impaired ISG induction, a state often associated with dysregulated immune and stress responses. Second, it establishes a new paradigm in virology, showing that an RNA virus can target an *m6A* eraser as a core immune-evasion strategy and highlighting the context-dependent roles of epitranscriptomic regulators in coordinating immune signaling and cellular stress adaptation. Finally, it unveils novel therapeutic opportunities. The essential nsp11 domain and the FTO-STAT2/3 interface represent promising nodes for antiviral intervention. Small molecules or peptides that disrupt the nsp11-STAT5 interaction or restore FTO activity could potentially rebalance antiviral immunity and mitigate stress-associated pathological responses. Together, these findings redefine PRRSV not merely as a veterinary pathogen but as a cross-species paradigm for RNA virus-mediated host reprogramming, demonstrating how a single viral protein can precisely reshape transcriptional and epitranscriptomic layers of host defense. In addition to suppressing interferon signaling, this regulatory axis is linked to inflammatory activation and oxidative stress, suggesting a broader role in coordinating immune-stress dynamics during infection. By mapping how a specific viral residue drives widespread changes in the host transcriptome, we provide mechanistic insight into how RNA viruses couple epitranscriptomic remodeling with immune and stress dysregulation. Therefore, this work establishes a conceptual framework in which manipulation of the FTO*-m6A* axis represents a conserved strategy by which RNA viruses rewire host responses to promote infection and persistence.

### Conclusion

This study reveals a mechanism by which an RNA virus hijacks host epitranscriptomic machinery to coordinate immune dysregulation and cellular stress responses during infection. The virus’s nsp11 protein actively suppresses the *m6A* demethylase FTO via a glycine-rich domain, with residues Q96 and S104 essential for virulence. FTO downregulation hypermethylates STAT2 and STAT3 mRNAs, reducing their translation and phosphorylation, thereby dampening a JAK-STAT-driven ISG program while essentially preserving STAT1 responses. This differential regulation reconciles the coexistence of impaired antiviral defense and excessive inflammatory responses, a state also associated with elevated oxidative stress during PRRSV infection. Together, these findings define the nsp11-STAT5-FTO-STAT2/3 axis as a mechanism linking RNA modification to coordinated immune and stress dysregulation. Given the conservation of the nsp11 NendoU domain, this pathway may represent a conserved strategy of host reprogramming among RNA viruses. Targeting *m6A* regulatory nodes such as FTO may therefore provide a means to restore immune balance and mitigate stress-associated pathology.

## MATERIALS AND METHODS

### Cells and viruses

Marc-145 (African green monkey kidney epithelial cell line) (36) and rPAMs (24, 37) were maintained at 37°C and 5% CO_2_ in Dulbecco modified Eagle medium (DMEM) and RPMI 1640 supplemented with 10% fetal bovine serum (FBS; GIBCO) containing 100 U/mL penicillin and 100 µg/mL streptomycin. rPAMs are an immortalized porcine alveolar macrophage cell line from primary PAMs, capable of stable passaging and supporting PRRSV infection. Widely used as an *in vitro* model for PRRSV replication and host response (24, 37). Primary pulmonary alveolar macrophage (PAM) cells were prepared and cultured in RPMI 1640 medium. The cryopreserved PAM cells were revived and precultured for 24 h before being used for virus inoculation, as previously described (38, 39).

The highly pathogenic (HP) PRRSV strain BB0907 (GenBank accession no. HQ315835.1) (40), the classical PRRSV S1 strain (C-PRRSV) (GenBank accession no. AF090173) (41, 42), and PRRSV FJ1402 (GenBank accession no. KX169191.1); all the strains were independently infected into Marc-145 cells and rPAMs. The inoculation was done at an MOI of 1 or using the amounts indicated in the figure legends or results. Recombinant viruses rBB/wt, rBB/Q96A, and rBB/S104A were derived from the infectious construct pCMV-BB0907 and propagated in rPAMs and Marc-145 cells. The median tissue culture infectious dose (TCID_50_) of PRRSV was determined in Marc-145 cells (43).

### Generation of FTO-knockout rPAM cells

FTO-knockout rPAM cells were generated using a CRISPR/Cas9-mediated genome editing approach. Two sgRNAs targeting conserved coding exons of the Sus scrofa FTO gene were designed using the Benchling online platform. Each sgRNA sequence was cloned into the lentiCRISPR v2 vector (Addgene #52961), which co-expresses SpCas9 and a puromycin resistance gene. All recombinant plasmids were verified by Sanger sequencing before use. rPAMs cells, which are capable of stable passaging, were seeded at 50%–60% confluence and transfected with the sgRNA-Cas9 plasmids using Lipofectamine 3000 (Invitrogen) according to the manufacturer’s instructions. At 48 h post-transfection, the cells were subjected to puromycin selection (2 μg/mL for 5–7 days) to enrich for successfully edited populations. Puromycin-resistant cells were subsequently subjected to single-cell cloning by limiting dilution in 96-well plates. Genomic DNA from individual clones was extracted using the QuickExtract DNA Extraction Solution (Lucigen), and the targeted FTO locus was PCR-amplified and Sanger-sequenced to confirm the presence of CRISPR-induced indels that lead to frameshift mutations. To validate the knockout efficiency, FTO mRNA and protein levels were assessed by qRT-PCR and western blotting, respectively, using an anti-FTO antibody (Abcam, ab92821). Clones exhibiting a complete loss of FTO expression were expanded and designated as rPAMs(ΔFTO) for all subsequent experiments.

### Animal experiments

All experiments were performed in accordance with relevant guidelines and regulations. Piglets were confirmed seronegative for PRRSV antibodies and PRRSV-free in blood; 6-week-old piglets were evenly divided into four groups (*n* = 5 per group). In the control group, piglets were given an intranasal injection of 2 mL of RPMI 1640 medium per piglet, while the groups inoculated with PRRSV strains (BB0907, S1, and FJ1402) received 2 mL of 1 × 10^5^ TCID_50_/mL PRRSV strains at the same time, respectively. The body weights and temperatures of the pigs were monitored weekly and daily. On day 28, post-infection, the pigs were euthanized, and all lung lobes were excised and collected.

### Hematoxylin and eosin (H&E) staining

Lung tissue sections were fixed in 4% paraformaldehyde. After fixation, the tissues were embedded in paraffin, sectioned, and stained with H&E for pathological evaluation of PRRSV invasion in lung tissue.

### Plasmid construction and transfection

The nsp11 of the PRRSV BB0907 strain was cloned into a pCAGGS-nsp11. The truncated fragments of nsp11 were subcloned from pCAGGS-nsp11 (including the N-terminal Flag tag) and respectively designated as pCAGGS-nsp11 (1–106), pCAGGS-nsp11 (91–223), and pCAGGS-nsp11 (107–223). The functional fragments were screened for alanine-substitution mutations in the nsp11 gene by PCR.

Porcine FTO, STAT1, STAT2, STAT3, and STAT5 were cloned into pCAGGS-Flag with an N-terminal Flag tag as previously described (21; 44). The whole PCR fragment was ligated into the pCAGGS vector (Invitrogen) to generate an N-terminal Flag tag fusion plasmid. Western blot was used to assess protein expression of each constructed plasmid, and the blots were analyzed with an anti-Flag antibody (Flag). All constructed plasmids were confirmed by DNA sequencing, and the primers are listed in Table S1. These plasmids were transiently transfected into cells using Lipofectamine 8000 (Beyotime, C0533).

The porcine FTO promoter sequence (GenBank accession number KM232950.1), spanning coordinates 1250–50 relative to the translation initiation site, was amplified from porcine MoDC genomic DNA, and the PCR product was verified by sequencing. The luciferase reporter plasmids pFTO-Luc and pΔFTO-Dual were constructed by cloning the FTO promoter and FTO mutant promoter fragment into the pGL3-Basic vector (Promega), which contains a modified firefly luciferase gene. The pRL-TK plasmid (Promega), containing the *Renilla* luciferase reporter, served as an internal control. All primers are shown in Table S2.

### siRNA transfection

Consistent with previously described (22), siRNAs specific for silencing FTO (si-FTO) and non-targeting siRNA control (si-NC) were transfected into cells using BioSune transfection reagent (Shanghai), according to the manufacturer’s instructions.

### Western blot analysis

Protein samples were separated by 10% sodium dodecyl sulfate-polyacrylamide gel electrophoresis (SDS-PAGE) and analyzed by western blotting as described previously to detect the protein levels and phosphorylated forms of each of the respective endogenous proteins (20, 21). The primary antibodies used included GBP1, CH25H, IFIT3, ISG15, IFITM1, Mx1, OSA, SOCS3, Usp18, STAT1, p-STAT1, STAT2, p-STAT2, STAT3, p-STAT3, STAT5, and p-STAT5 (1:1,000; Proteintech), N protein (1:1,000; GeneTex), nsp11, Flag (1:2,000; Abmart), GAPDH (1:10,000; Abcam), and FTO (1:1,000; Proteintech). Secondary antibodies were goat anti-rabbit or goat anti-mouse IgG-conjugated to horseradish peroxidase (1:1,000; Abcam, MA, USA). An enhanced chemiluminescence (ECL) system (Beyotime, P0018M) was used to visualize immunoreactive proteins. All experiments were repeated at least three times to ensure the reliability of the results.

### Real-time quantitative PCR (RT-qPCR)

Total RNA was extracted from rPAMs and Marc-145 cells using the Qiagen RNeasy kit according to the manufacturer’s instructions. Subsequently, RNA was reverse-transcribed using the cDNA synthesis kit from TaKaRa as per the manufacturer’s instructions. RT-qPCR analyses were conducted in triplicate using the Power SYBR Green PCR master mix from Invitrogen, as previously described (40; 45), to assess the gene expression of FTO, N protein, nsp11, STAT1, STAT2, STAT3, STAT5, ISGs, and GAPDH. The primer sequences for RT-qPCR are listed in Table S3.

### Co-immunoprecipitation assay

Immunoprecipitation was performed using an immunoprecipitation kit (Beyotime, P2197). The procedure involved mixing 3 μg of the appropriate Flag and FTO antibodies with 20 μL of magnetic beads and incubating for 1 h. After draining the supernatant, cell lysates containing the antigen were incubated with the bead-antibody complex overnight at 4°C (no nuclease was added to the lysis buffer during the co-immunoprecipitation assay). The bead-antibody-antigen complexes were washed three times with lysis buffer, and the immunoprecipitated samples were suspended in SDS loading buffer. Western blot analysis was performed on the collected samples.

### RNA immunoprecipitation assay

The enrichment of FTO in the ISGs *m6A* region was measured using a Pure Binding RNA immunoprecipitation kit (Geneseed) according to the manufacturer’s instructions. The cells were lysed with RIP lysis buffer at 4°C for 10 min, and the sample was split into three 100-μL aliquots. Two parts were submitted to RIP using protein A/G Sepharose beads coupled with an anti-FTO antibody (5 μg, Proteintech) and normal rabbit IgG (Abcam) at 4°C for 6 h. The RNA, combined with the antibodies, was purified and dissolved in RNase-free water. RT-qPCR was performed to quantify ISG transcripts in immunoprecipitated RNA and total RNA from whole-cell lysates.

### The *m6A* dot blot assay

Total RNA from cells was extracted using TRIzol reagent (Tiangen, China), and RNA concentration was measured using a Nanodrop 2000 (Thermo Fisher Scientific, Waltham, MA, USA). The dot blot assay was performed according to the Bio-Protocol Database (46). Specifically, RNA samples were heat-denatured at 65°C for 10 min in a thermal cycler and then quickly cooled on ice. A total of 1 μg RNA was loaded on nylon membranes. Subsequently, the membrane was cross-linked with UV, blocked with 5% non-fat dry milk in 10 mL TBS for 1 h, and incubated with a specific anti-*m6A* antibody (1:1,000, Proteintech) at 4°C overnight with gentle shaking. Next, the membranes were incubated with HRP-conjugated goat anti-mouse IgG (1:2,000, HuaAnbio) and ECL Western Blotting Substrate (Bio-Rad) for 5 min in the dark at room temperature. The membranes were visualized using Immobilon Western Chemilum HRP Substrate (Merck Millipore), washed once with TBST buffer for 5 min, and stained with MB for 30 min. Relative *m6A* levels were determined by densitometry quantification and normalized to MB staining.

### *m6A*-seq analysis

*m6A*-seq was conducted in rPAMs assigned to four groups (NC, PRRSV, PRRSV + FTO OE, and PRRSV + FTO KO). Total RNA was extracted 24 h post-infection/transfection using TRIzol and assessed for quality. Poly(A)+ RNA purified from 50 to 100 μg total RNA was fragmented to 100–200 nt, and 300–500 ng fragmented mRNA was incubated with anti-*m6A* antibody (Abcam, ab151230) and Dynabeads protein A in IP buffer. After sequential low-, medium-, and high-salt washes, m⁶A-enriched RNA was eluted with proteinase K, purified, and processed in parallel with input RNA for library construction using the NEBNext Ultra RNA Library Prep Kit.

Libraries were sequenced on an Illumina HiSeq platform (PE150). After quality control and adaptor trimming, high-quality reads were aligned to the *Sus scrofa* genome (Sscrofa11.1) using HISAT2. m⁶A peak calling and differential methylation analyses were performed using exomePeak2 and MeTDiff with default settings.

### RNA-seq analysis

RNA-seq was performed in rPAMs assigned to four groups: negative control, PRRSV infection, PRRSV + FTO overexpression, and PRRSV + FTO knockout. Total RNA was extracted at 24 h post-infection/transfection using TRIzol, and RNA integrity was confirmed using a NanoDrop and an Agilent Bioanalyzer (RIN ≥ 8). High-quality RNA was used for library preparation with the NEBNext Ultra RNA Library Prep Kit, followed by Illumina NovaSeq 6000 sequencing (paired-end 150 bp).

Sequencing reads were trimmed with Cutadapt, assessed using FastQC, and aligned to the *Sus scrofa* (Sscrofa11.1) genome using STAR. Gene-level read counts were obtained with featureCounts, and genes with low abundance were removed prior to differential expression analysis using DESeq2 (|fold change| ≥ 1.3, FDR ≤ 0.05). TPM-normalized expression matrices were used for PCA and clustering, and GO/KEGG enrichment analyses were performed with clusterProfiler.

### Function enrichment assay

Kyoto Encyclopedia of Genes and Genomes (KEGG) (47) and Gene Ontology (GO) (48) enrichment analyses were performed using the Database for Annotation, Visualization, and Integrated Discovery (DAVID) online analysis site (https://www.omicsmart.com) to determine which signaling pathways the intersecting differentially expressed genes (DEGs) were enriched in. First, the DEG list was uploaded to the “Upload” module, and Homo sapiens was selected as the analysis species. The website filters out unrecognized genes, selects items for analysis, and then performs enrichment testing against KEGG pathways, GO biological process, molecular function (MF), and cellular component terms. Adjusted *P*-values <0.05 were considered statistically significant. The results were visualized using the “ggplot2” package in R.

### Immunofluorescence and confocal laser assay

The cells were transfected with the vector pCAGGS or pCAGGS nsp11 plasmids. At 24 h post-transfection, the pretreated cells were fixed in precooled methanol at 4°C for 10 min and then permeabilized with 0.5% Triton X-100 for 10 min. After three washes with PBS, the cells were incubated with 10% bovine serum albumin (BSA) at 4°C, then with the primary antibody for 12 h. Subsequently, the cells were incubated with the secondary antibody at 37°C for 2 h. The cells’ nuclei were stained with 4′,6′-diamidino-2-phenylindole (DAPI, Invitrogen, China) for 5 min and washed with PBS. Fluorescent images were acquired using a fluorescence microscope (Olympus, Japan) and a confocal laser-scanning microscope (SP8, Leica, Germany).

### Dual-luciferase reporter assay

The FTO promoter prediction analysis identified key transcriptional sites for STAT3 and STAT5, which were knocked down (Fig. 6A). Promoter mutants of STAT3 and STAT5 were then generated to confirm their interaction with nsp11. Cells were transfected with or without nsp11, FTO promoter, or mutant FTO promoter, and pRL-TK (49). After 48 h, the biofluorescence emitted during luciferin oxidation was measured using a dual-luciferase reporter assay system (Promega) according to the manufacturer’s protocol.

### Construction of infectious cDNA clones of PRRSV

The full-length PRRSV genome was amplified using the primers listed in Table S4. Mutant nsp11, pEasy-D, was constructed in the PRRSV BB0907 background as previously described (24, 40, 20). Briefly, the D fragment of a full-length cDNA clone (pCMV-BB) underwent nsp11 mutagenesis. Three plasmids, pBB/D-nsp11 (Q96A), pBB/D-nsp11 (D99A), and pBB/D-nsp11 (S104A), containing the D fragment, were designated and used as templates for site-directed mutagenesis. Notably, the pEasy-D plasmid was generated through splice overlap extension PCR using pBB/D-nsp11 (Q96A), pBB/D-nsp11 (D99A), and pBB/D-nsp11 (S104A) (Fig. 8B).

To rescue chimeric viruses, Marc-145 cells were transfected with the pBB/D-nsp11 plasmid using Lipofectamine 8000 reagent according to the manufacturer’s protocol. The cell culture supernatants collected at 96 h post-transfection were then passaged four times consecutively in fresh Marc-145 cells until approximately 80% of the cells displayed cytopathic effects (CPEs). RNA was extracted from the recovered mutant viruses, designated rBB/wt, rQ96A, rD99A, and rS104A, for cDNA synthesis and DNA sequencing to confirm the presence of the mutant nucleotides.

### One-step viral growth curves

Subconfluent Marc-145 cells in 24-well plates were inoculated with 10^6^ TCID_50_ of PRRSV mutants; 100 μL of infected-cell supernatant was collected from each culture at 6, 12, 24, 36, 48, and 72 h post-infection (hpi), and the same volume of fresh medium was added. The virus titers at each time point were determined by the TCID_50_ assay as follows: the growth curve was plotted with time on the horizontal axis and viral titer on the vertical axis.

### Statistical analysis

The quantitative analysis was based on data from at least three independent experimental trials. Subsequently, the data were presented as the mean ± standard deviation (SD) to ensure accuracy and reproducibility. Statistical analysis was performed using GraphPad software version 10.0, and differences between the two groups were assessed using a Student’s *t*-test. One-way ANOVA was used to compare differences among multiple groups, followed by Tukey’s multiple comparisons test. The significance level is indicated in the figures as *, *P* < 0.05; **, *P* < 0.01; ***, *P <* 0.001; and ns, not significant.

## Data Availability

The m⁶A-seq data generated in this study have been deposited in the NCBI Sequence Read Archive under BioProject accession number PRJNA1499961. All data needed to evaluate the conclusions in the paper are present in the paper and/or the supplemental material.

## SUPPLEMENTAL MATERIAL

Supplemental figures part 1 mbio.00843-26-s0001.pdf Fig. S1 to S31.

Supplemental figures part 2 mbio.00843-26-s0002.pdf Fig. S32 to S57.

Supplemental tables mbio.00843-26-s0003.pdf Tables S1 to S4.

ASM does not own the copyrights to Supplemental Material that may be linked to, or accessed through, an article. The authors have granted ASM a non-exclusive, world-wide license to publish the Supplemental Material files. Please contact the corresponding author directly for reuse.
