# Supplementary material for: PRRSV suppresses FTO-dependent m6A demethylation to reprogram STAT signaling and innate immunity
Source: mBio. 2026 Aug 13;17(9):e00843-26. doi: 10.1128/mbio.00843-26 (PMC13556206; doi:10.1128/mbio.00843-26)
Supplement: Supplementary file 1 [file mbio.00843-26-s0001.pdf]

Supporting Information

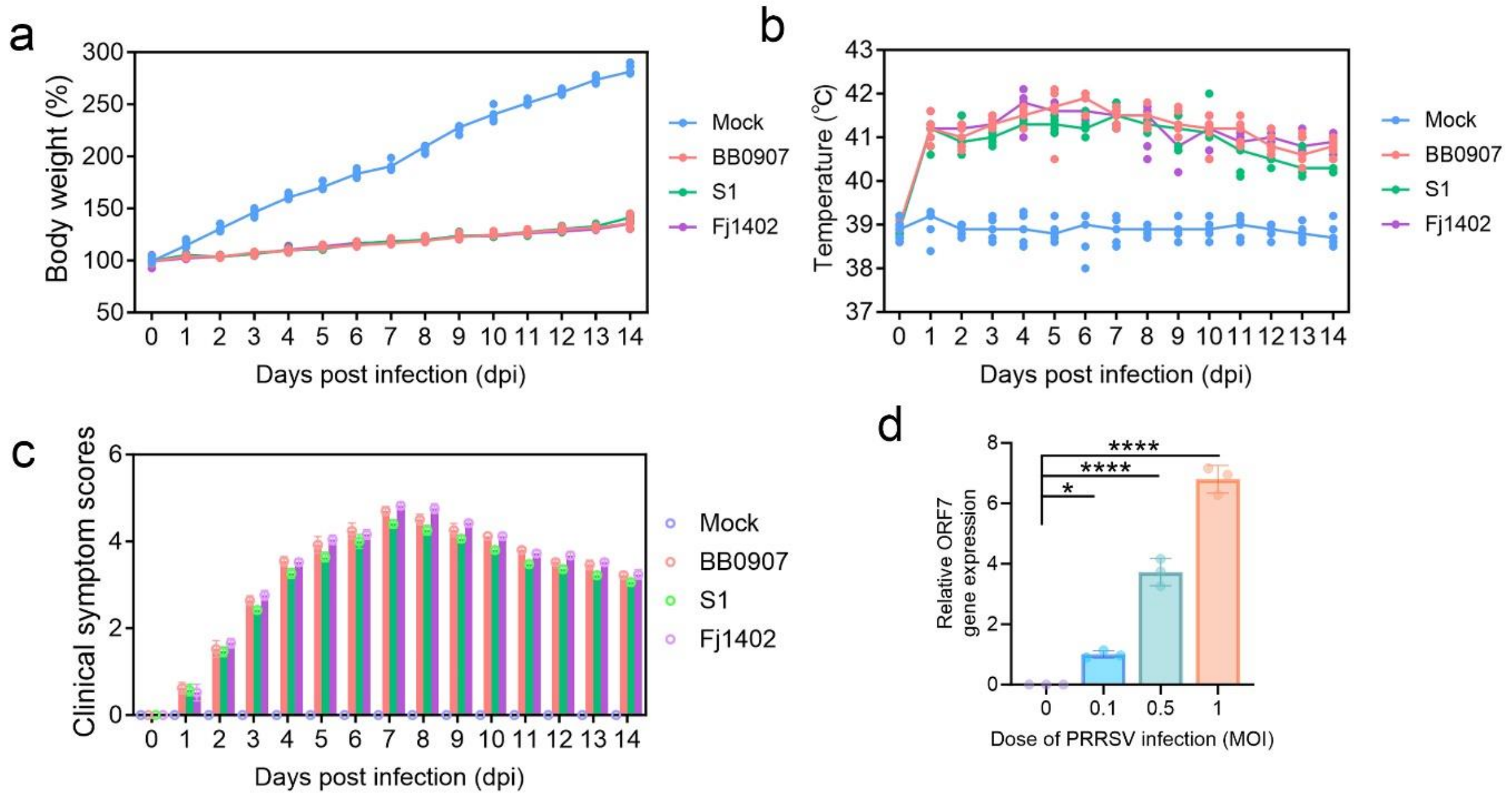

Fig. S1. (a) Body weight changes of piglets infected with PRRSV strains BB0907, S1, or FJ1402 compared to Mock control over 14 days post-infection (dpi). (b) Rectal temperature of piglets monitored daily during the infection period. (c) Clinical symptom scores recorded daily post-infection for each group (n = 5 per group). (d) PAMs were infected with PRRSV at the indicated MOIs, and the viral ORF7/N transcript level was measured by RT-qPCR. GAPDH was used as the internal control (n = 3 per group).

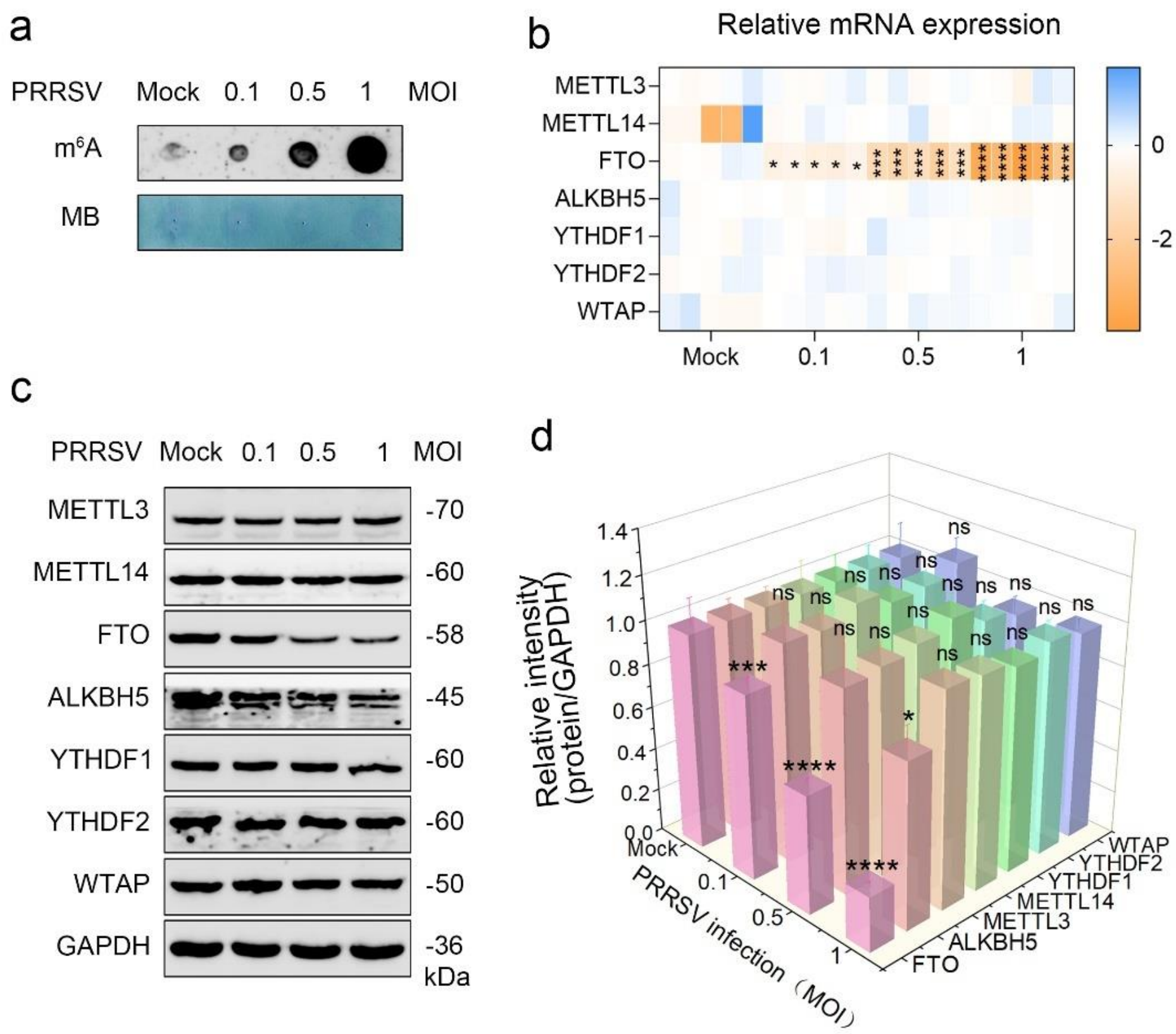

Fig. S2. (a) Dot blot analysis of *m6A* RNA modification levels in Marc-145 cells following PRRSV infection with different MOIs (0, 0.1, 0.5, 1). Methylene blue (MB) served as the loading control. (b) Heatmap of mRNA expression of *m6A* regulators (METTL3, METTL14, FTO, ALKBH5, YTHDF1, YTHDF2, WTAP) in Marc-145 cells after PRRSV infection. (c, d) Western blot (c) and quantification analysis (d) of relative protein expression levels of *m6A* regulators normalized to GAPDH in Marc-145 cells infected with PRRSV at different MOIs. GAPDH served as a loading control.

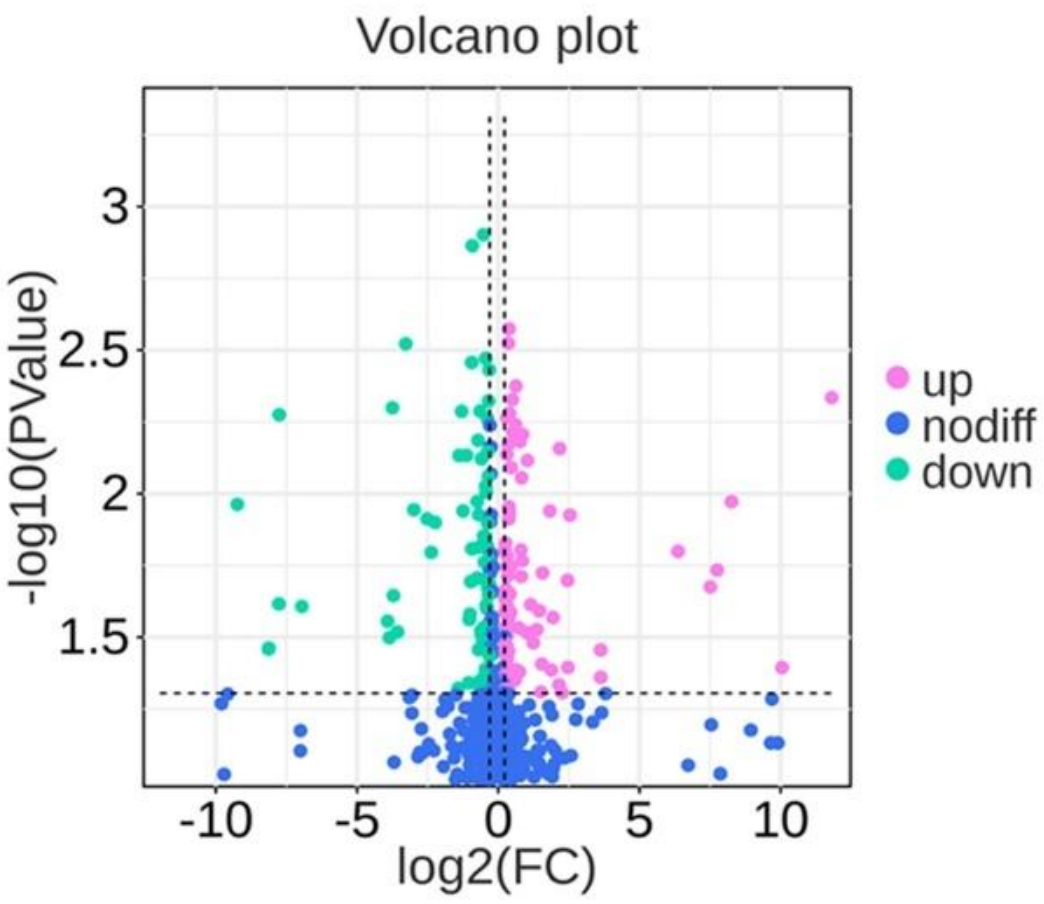

Fig. S3. Volcano plot of differentially expressed genes (DEGs) between control and FTO-overexpressing (Oe-FTO) rPAMs from RNA-seq data. Genes with $|\log_2(\text{fold change, FC})| \geq 1.3$,

a false discovery rate (FDR) ≤ 0.05 are shown in pink (upregulated) and green (downregulated). Non-significant genes are shown in blue. RNA-seq was performed with five biologically independent replicate**s** per condition, harvested at 36 h post-transfection. Sequencing was carried out by BGI Genomics on the Illumina NovaSeq 6000 platform.

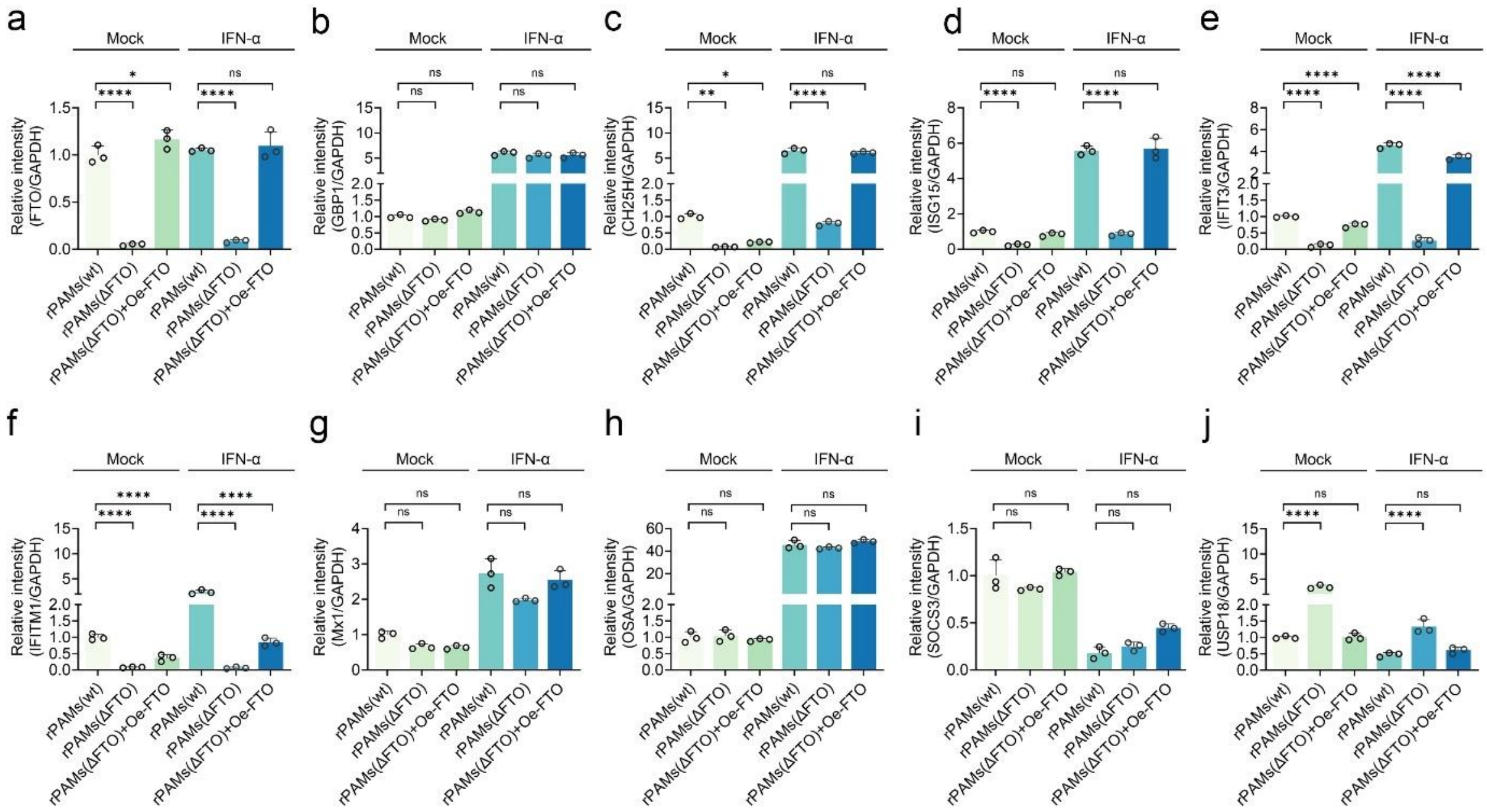

Fig. S4. rPAMs (wild-type and FTO knockout, ΔFTO) were treated with or without IFN-α, and transfected with or without FTO overexpression plasmid (Oe-FTO). The quantification analysis of FTO and ISG proteins, including GBP1, CH25H, ISG15, IFIT3, IFITM1, Mx1, OSA, SOCS3, and USP18.

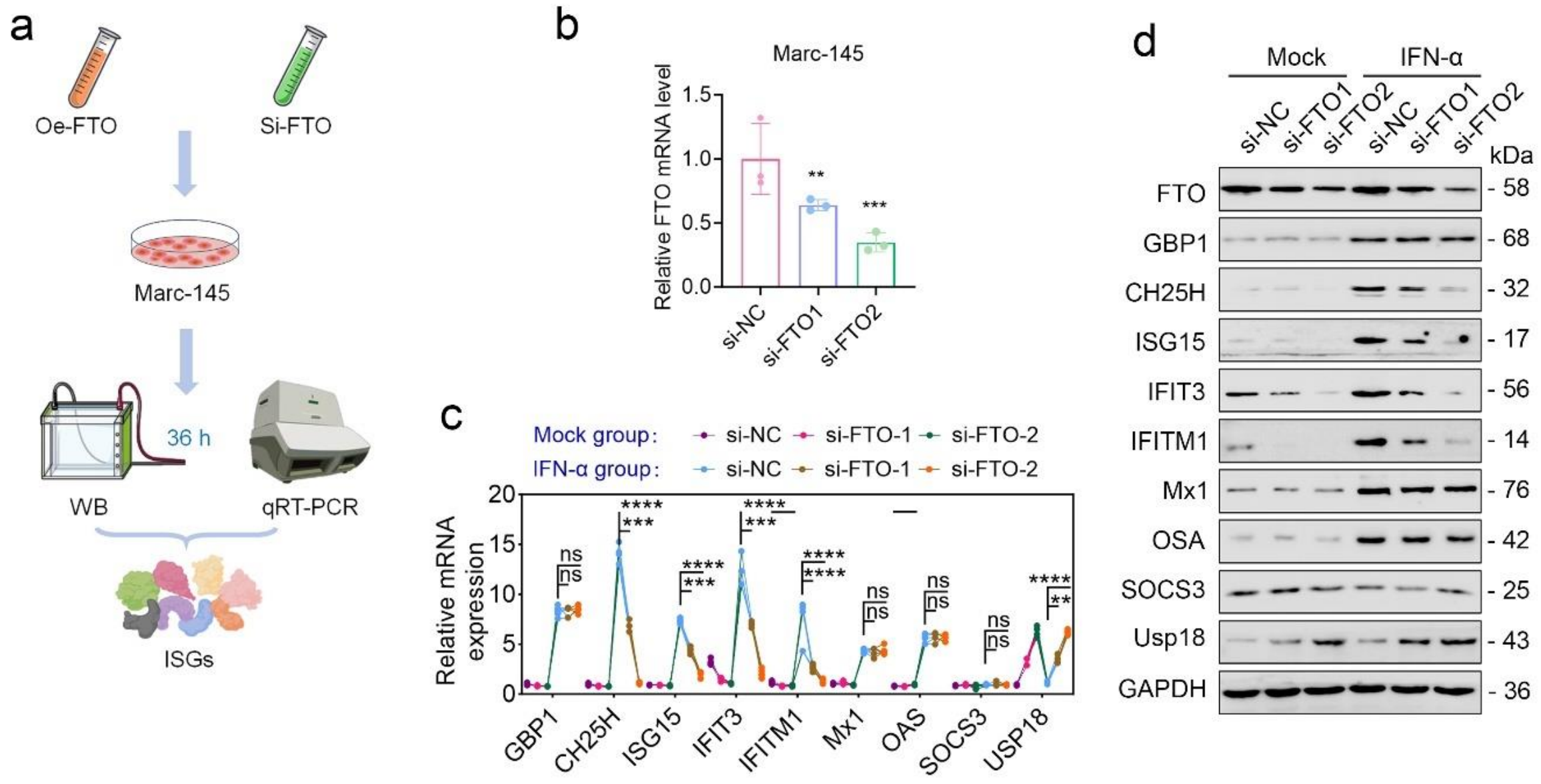

Fig. S5. (a) Schematic illustration of Marc-145 cells transfected with FTO plasmid overexpression (Oe-FTO) and silenced FTO plasmid (si-FTO). (b) Marc-145 cells transfected

with si-NC, si-FTO1 (-652), and si-FTO2 (-947). qRT-PCR analysis showing relative FTO mRNA levels in Marc-145 cells transfected with si-NC, si-FTO1, or si-FTO2. (c-d) Marc-145 cells transfected with si-NC, si-FTO1 (-652), and si-FTO2 (-947), with IFN-α as a positive control. (c) qRT-PCR analysis of ISGs mRNA level. (d) Western blot analysis of FTO and ISGs (FTO, GBP1, CH25H, ISG15, IFIT3, IFITM1, Mx1, OSA, SOCS3, and Usp18) protein levels.

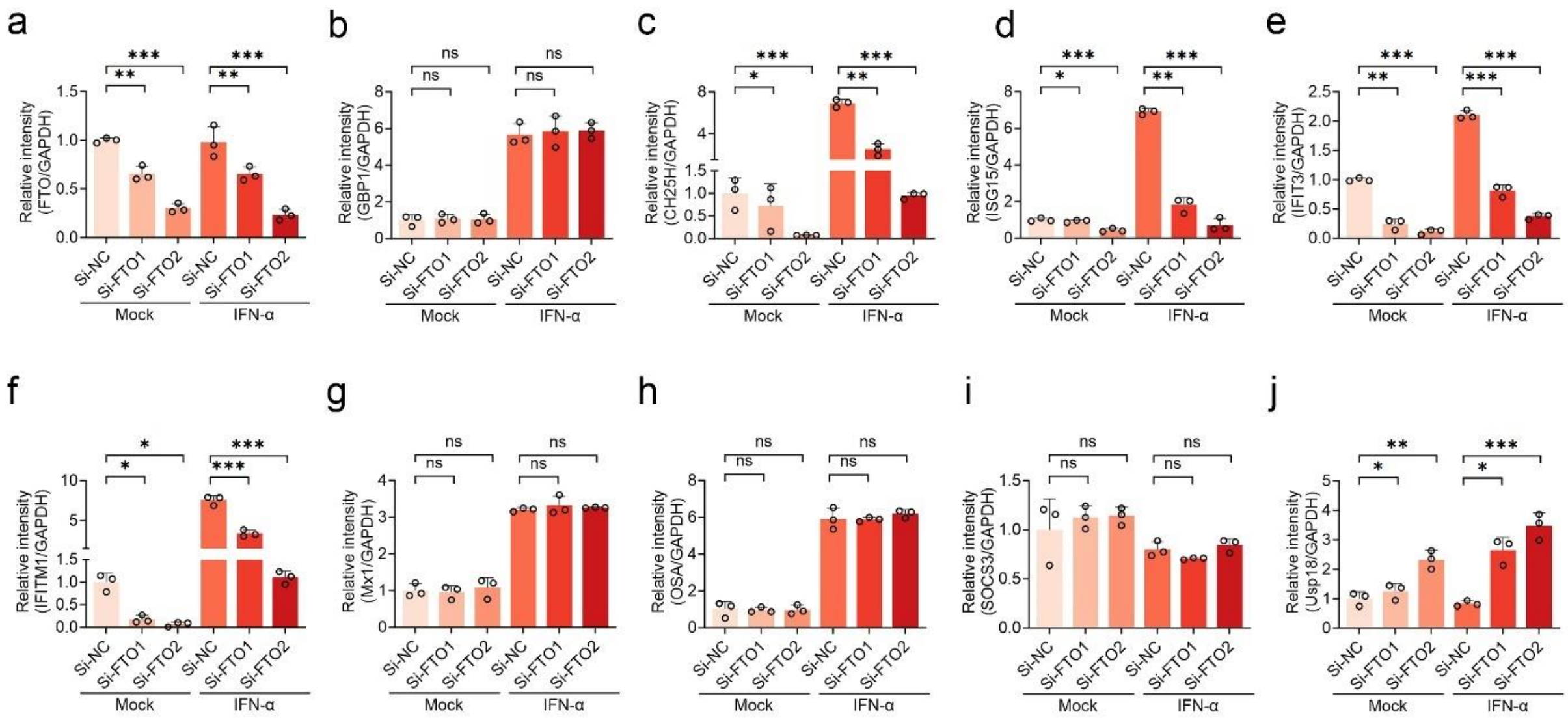

Fig. S6. Marc-145 cells transfected with si-NC, si-FTO1 (-652), and si-FTO2 (-947), with IFN-α as a positive control. The quantification analysis of FTO and ISG proteins, including GBP1, CH25H, ISG15, IFIT3, IFITM1, Mx1, OSA, SOCS3, and USP18.

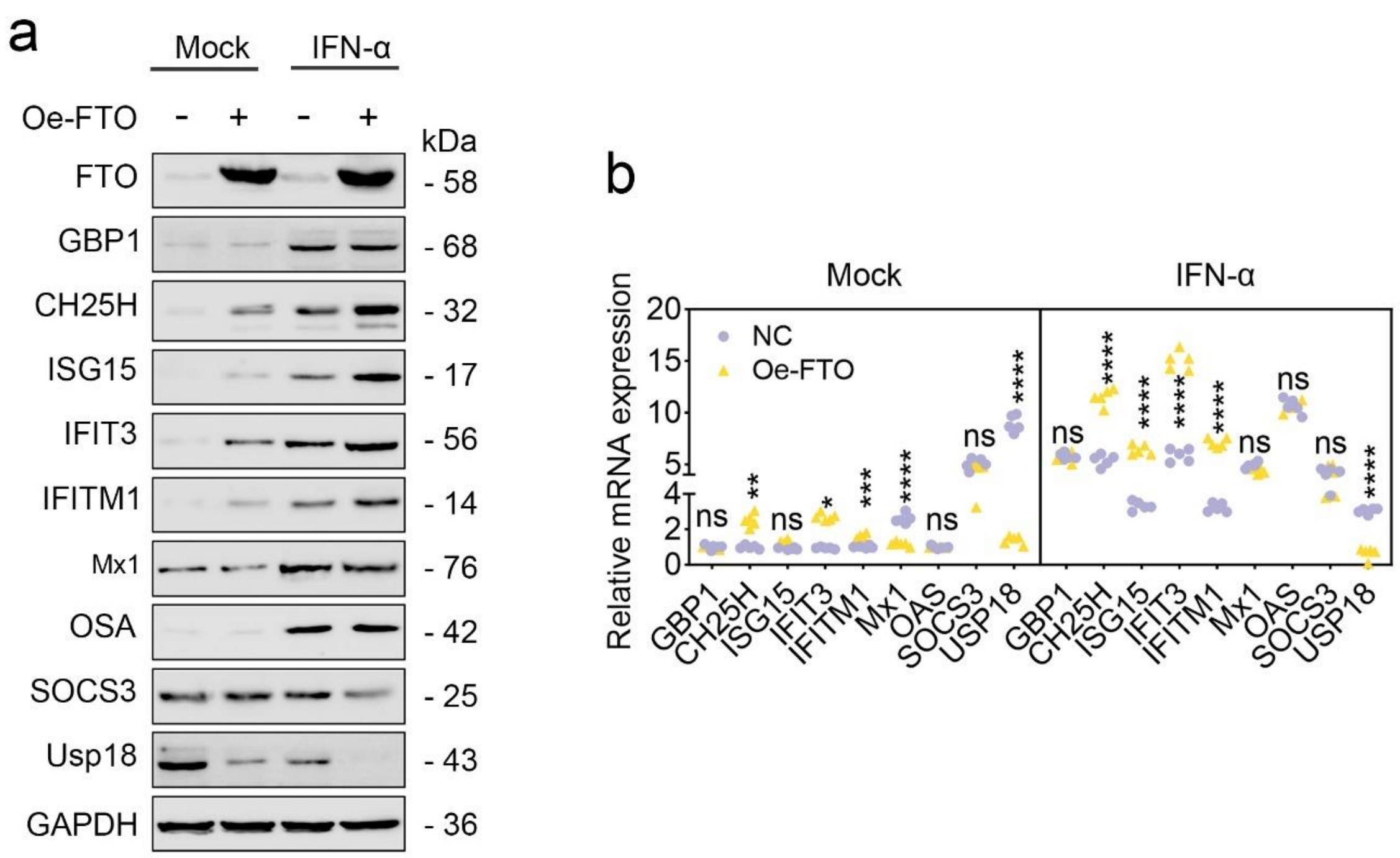

Fig. S7. Marc-145 cells were treated with or without IFN-α and transfected with Oe-NC and Oe-FTO. (a) Western blot analysis showed the protein levels of FTO and ISGs. (b) ISG expressions measured by qRT-PCR.

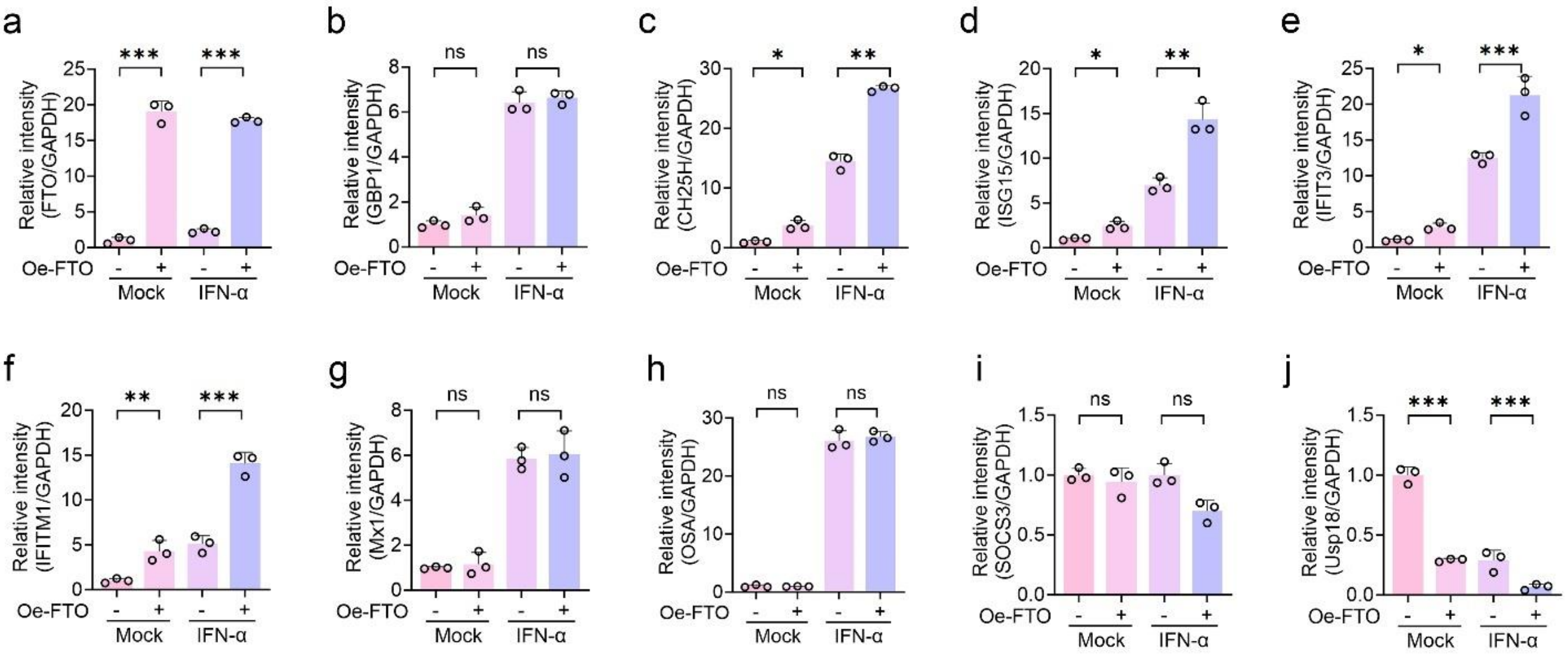

Fig. S8. rPAMs were treated with or without IFN-α and transfected with Oe-NC and Oe-FTO. The quantification analysis of FTO and ISG proteins, including GBP1, CH25H, ISG15, IFIT3, IFITM1, Mx1, OSA, SOCS3, and USP18.

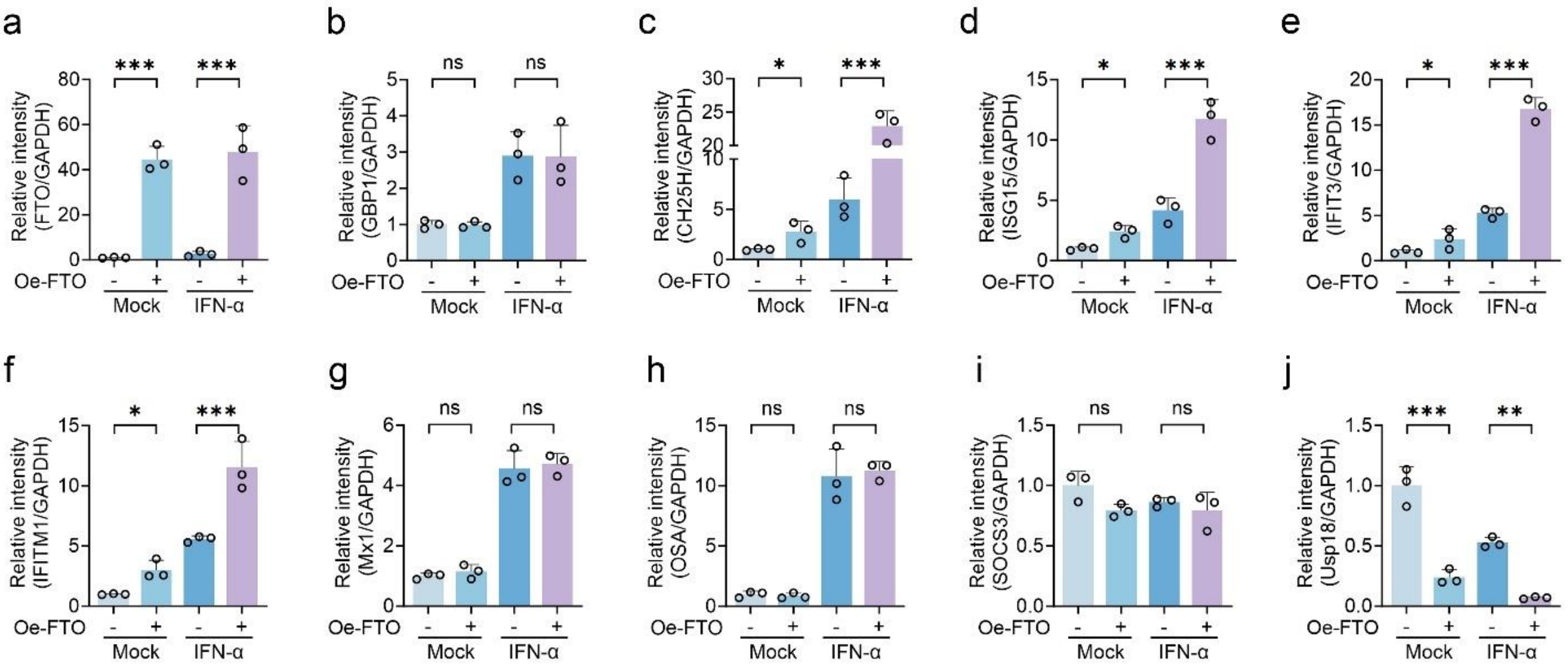

Fig. S9. Marc-145 cells were treated with or without IFN-α and transfected with Oe-NC and Oe-FTO. The quantification analysis of FTO and ISG proteins, including GBP1, CH25H, ISG15, IFIT3, IFITM1, Mx1, OSA, SOCS3, and USP18.

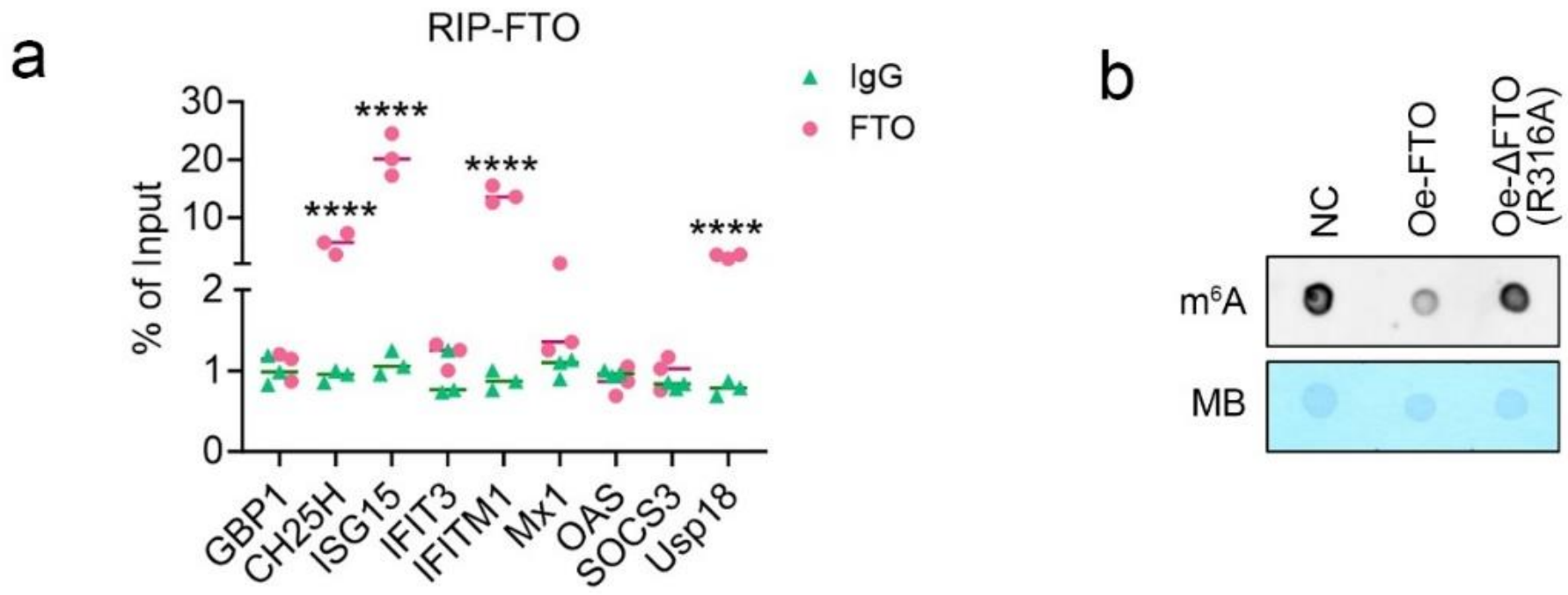

Fig. S10. (a) RIP-qPCR enrichment assay of FTO on the ISGs mRNA *m6A* region in Marc-145 cells. (b) Marc-145 cells were transfected with Oe-FTO and mutant Oe-ΔFTO (R316A). Dot blot analysis of *m6A* modification levels, and MB (methylene blue) staining was used as the loading control.

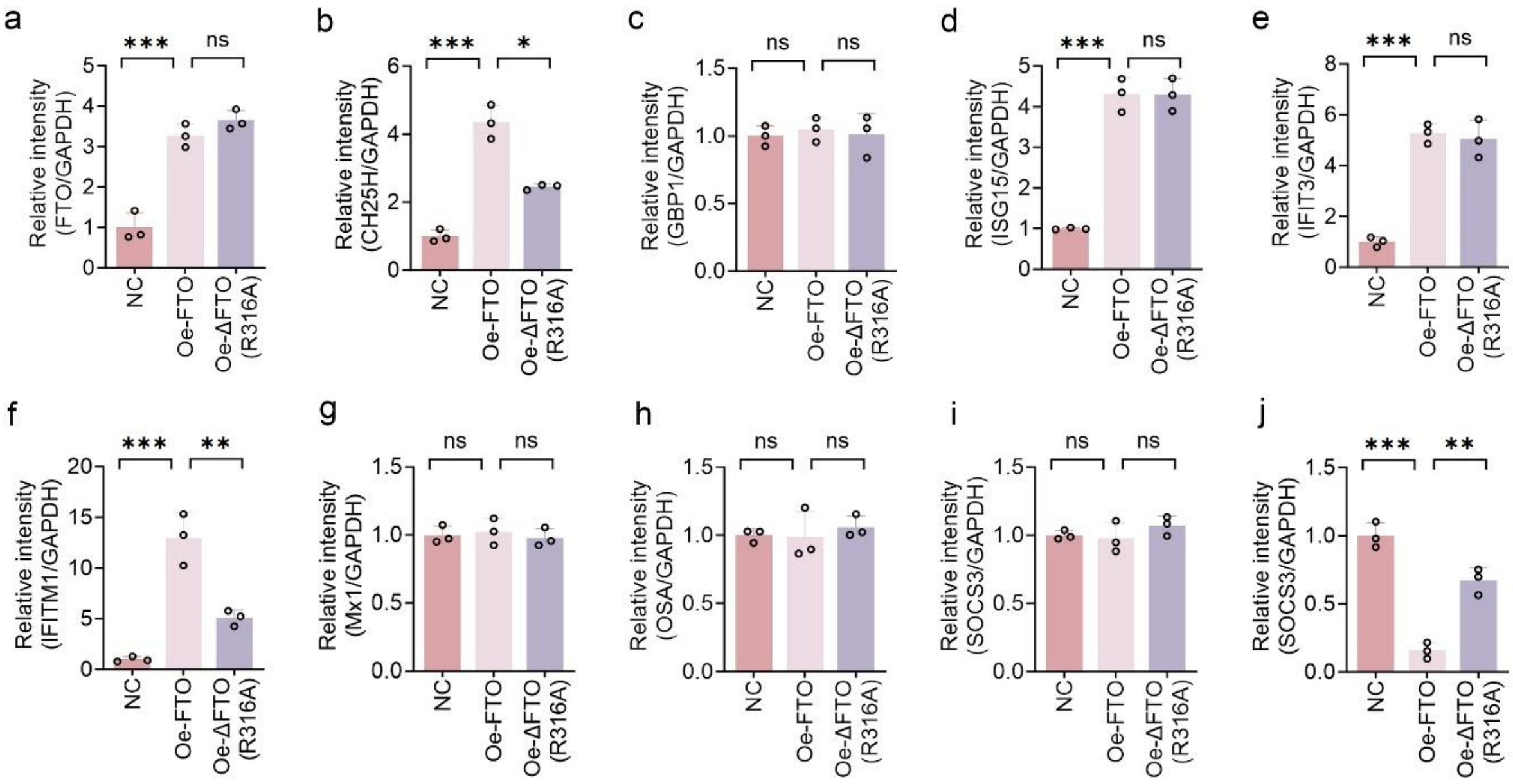

Fig. S11. rPAMs were transfected with Oe-FTO and mutant Oe-ΔFTO (R316A). The protein quantification of FTO and ISGs expression.

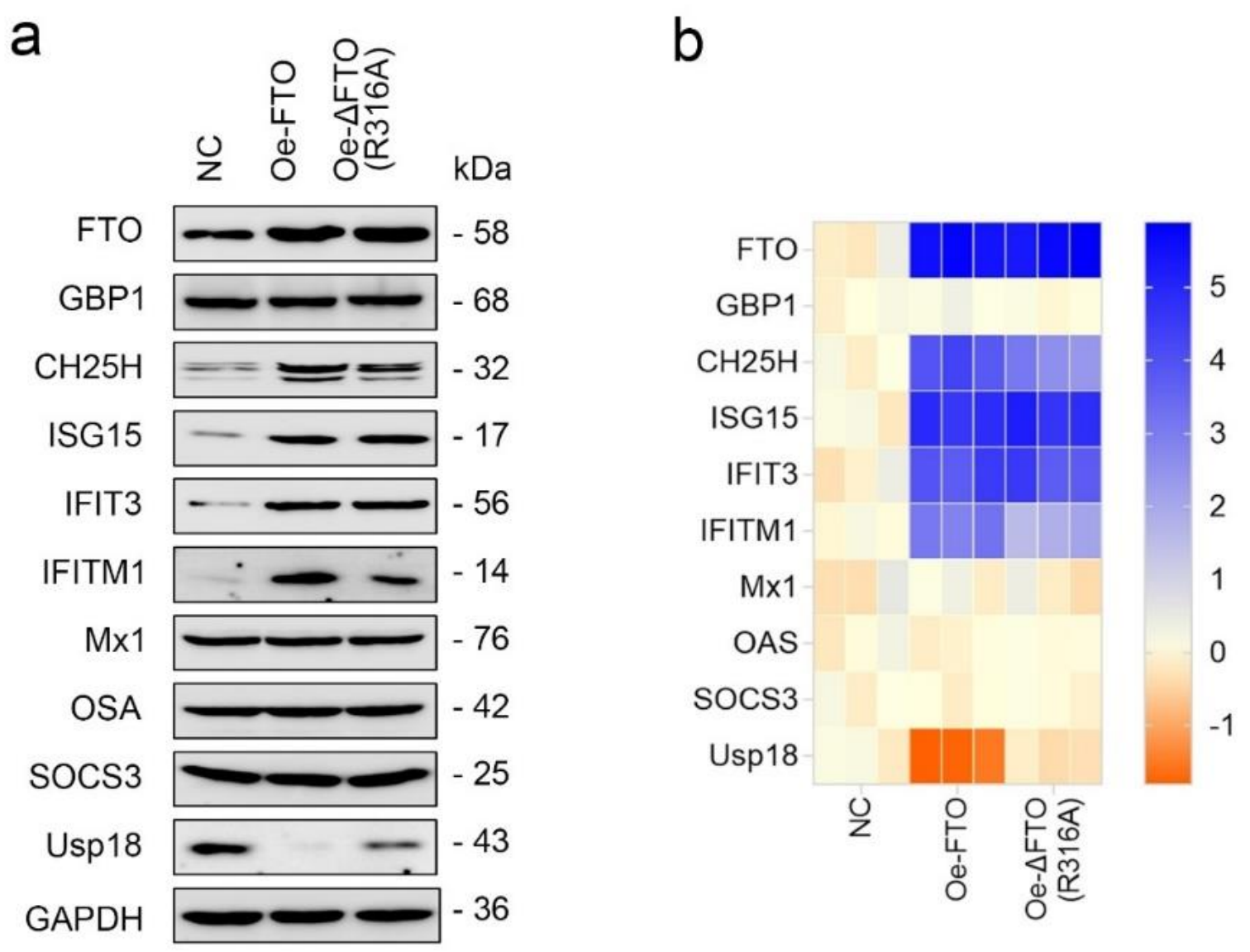

Fig. S12. Marc-145 were transfected with Oe-FTO and mutant Oe-ΔFTO (R316A). (a) Western blot analysis of FTO and ISGs protein expressions. (b) Heat map of FTO and ISG mRNA expression levels. The color bar indicates expression levels from low (orange) to high (blue).

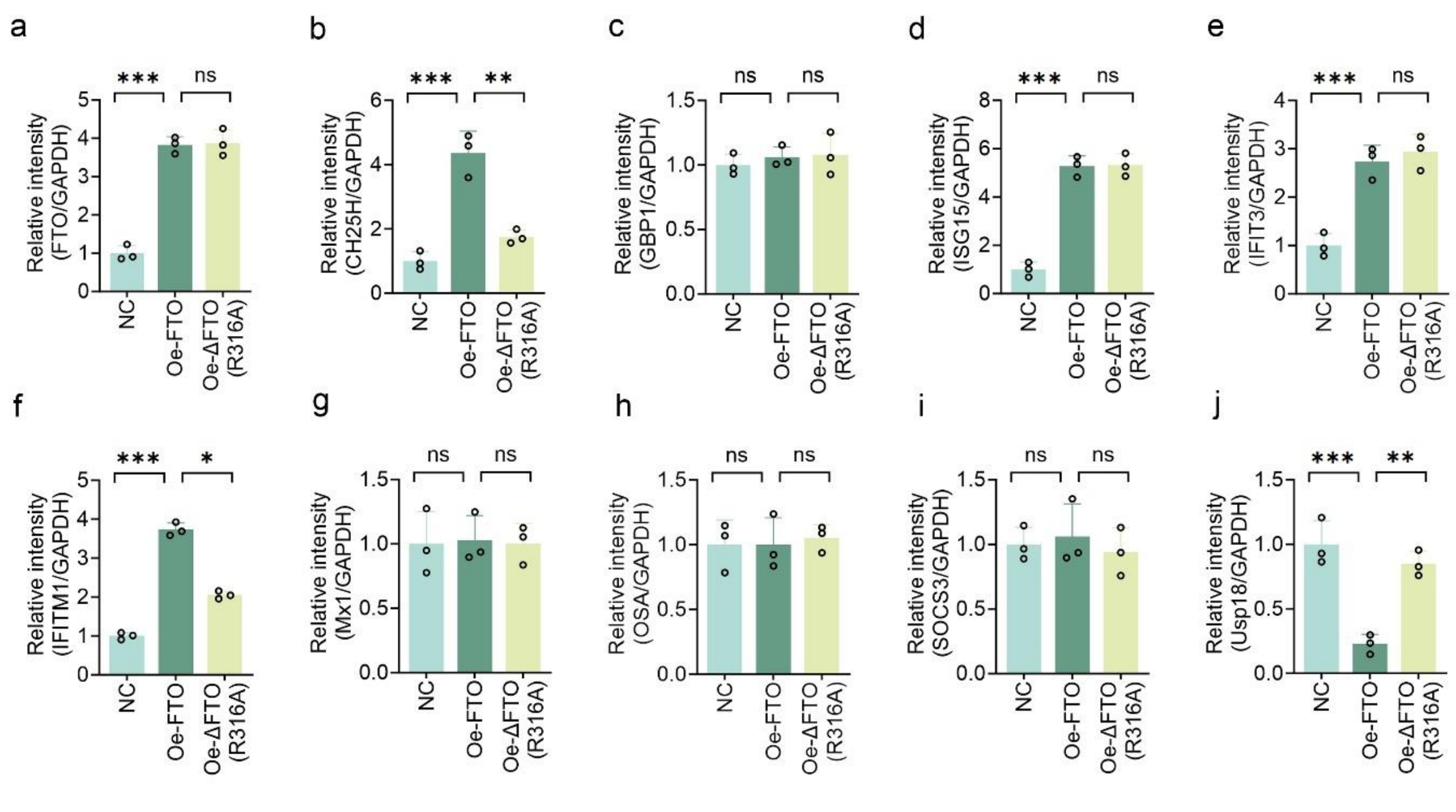

Fig. S13. Marc-145 were transfected with Oe-FTO and mutant Oe-ΔFTO (R316A). The protein quantification of FTO and ISGs expression levels.

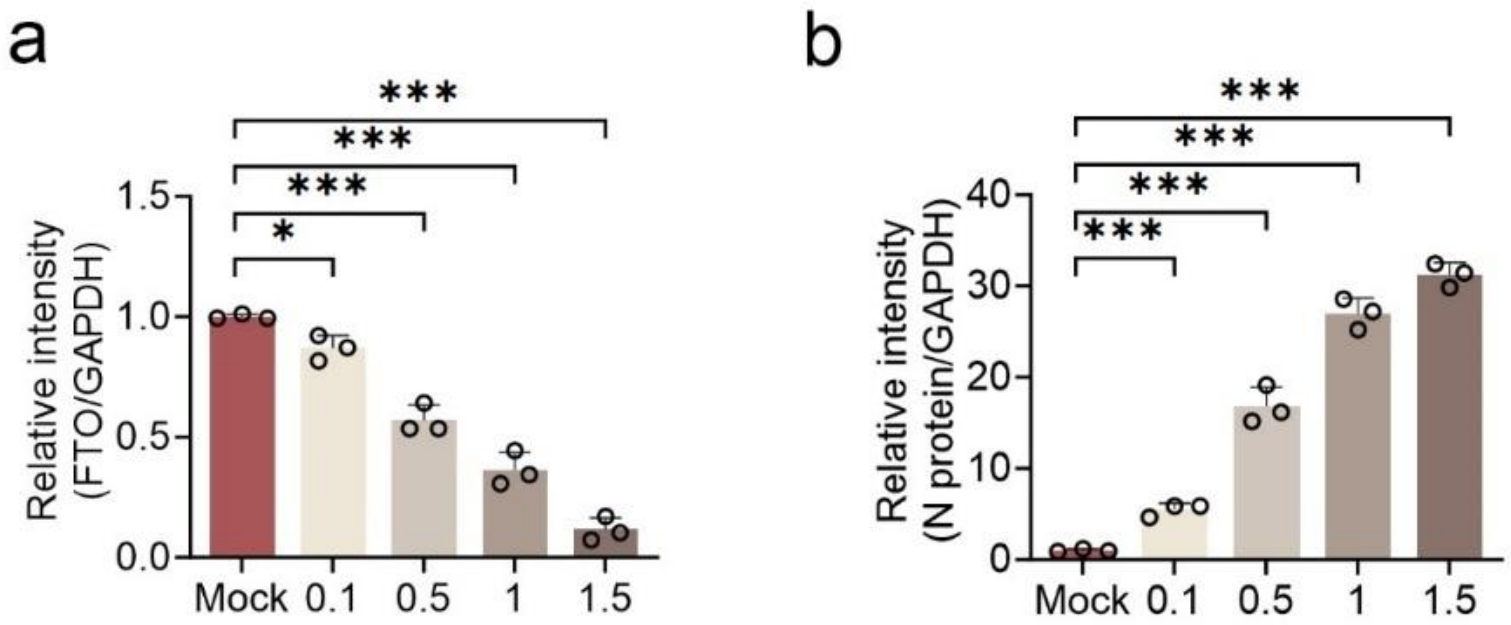

Fig. S14. rPAMs infected with PRRSV at MOIs of 0.1, 0.5, 1, and 1.5 for 36 h. The quantification of (a) FTO and (b) PRRSV N protein levels.

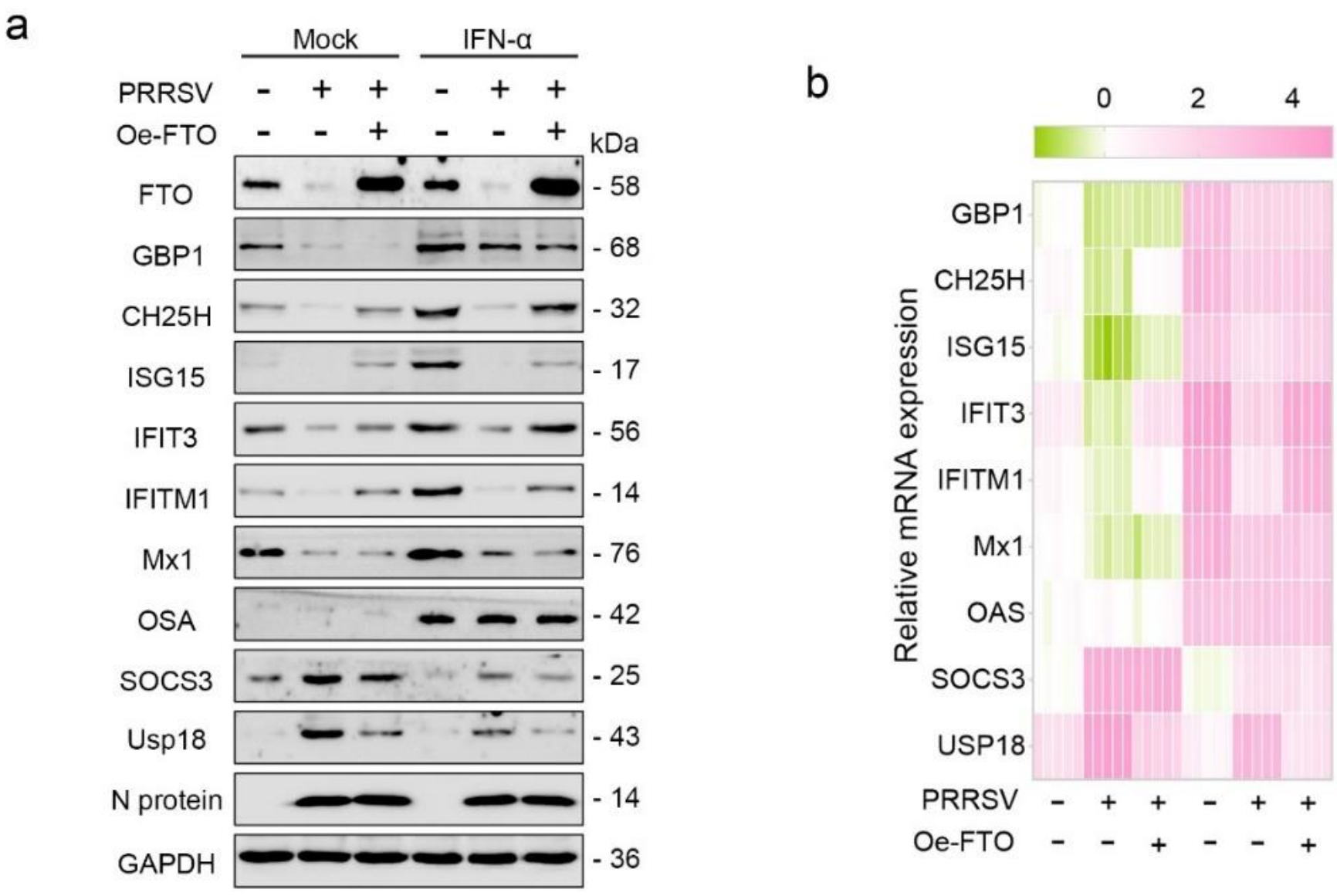

Fig. S15. Marc-145 cells were transfected with Oe-FTO 1 h post-PRRSV infection. (a) Western blot analysis of Marc-145 cells following the treatment protocol. (b) qRT-PCR analysis of ISGs mRNA level.

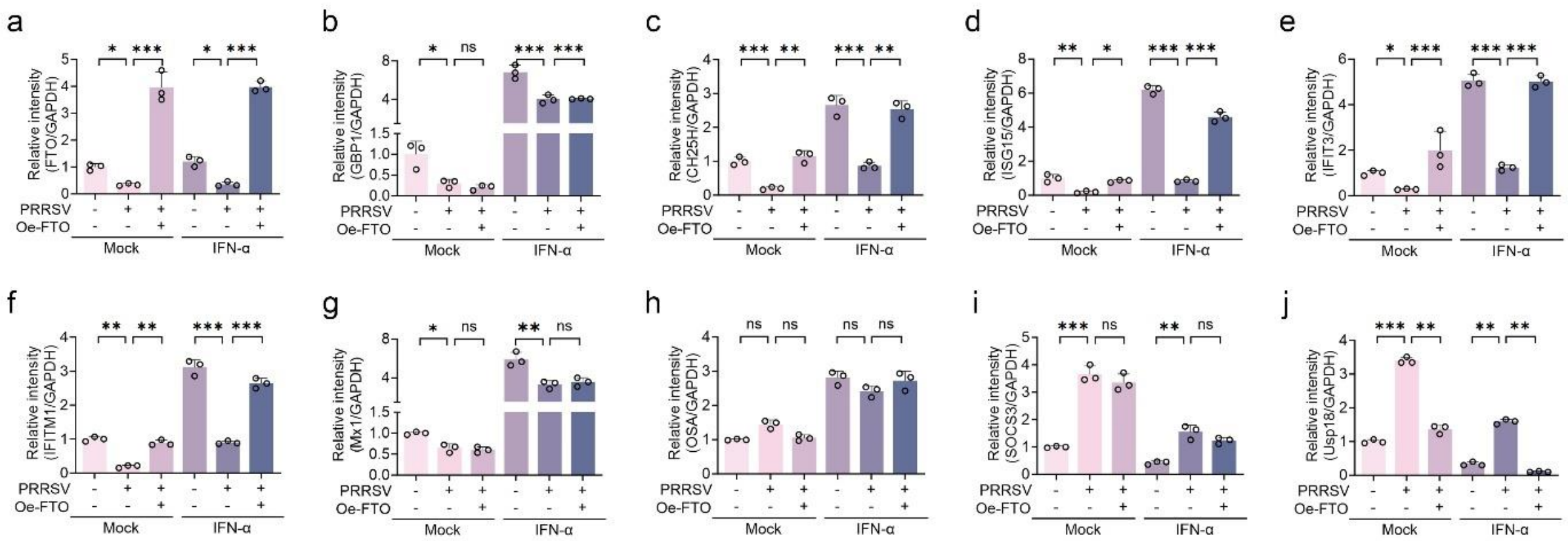

Fig. S16. rPAMs were transfected with Oe-FTO 1 h post-PRRSV infection. The protein quantification of FTO and ISGs expression levels.

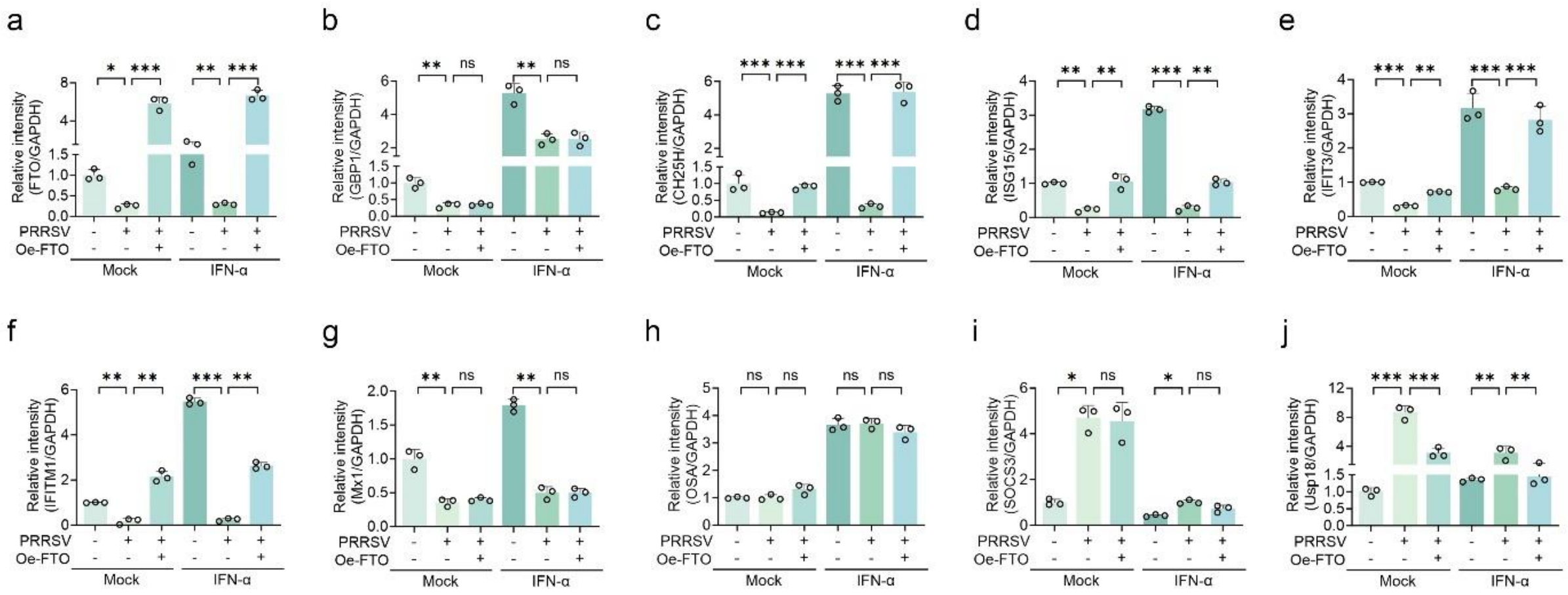

Fig. S17. Marc-145 cells were transfected with Oe-FTO 1 h post-PRRSV infection. The protein quantification of FTO and ISGs expression levels.

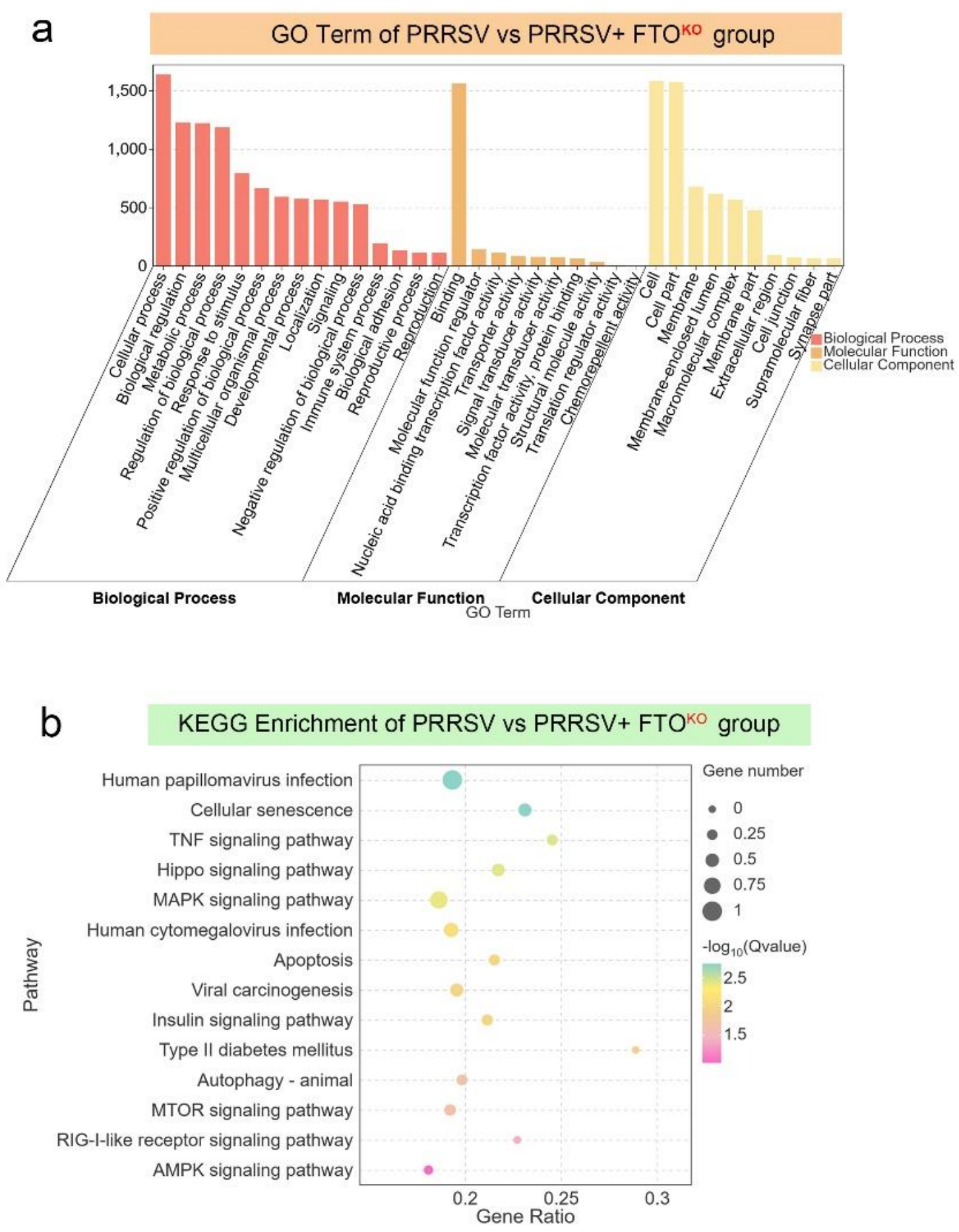

Fig. S18. (a) Gene Ontology (GO) enrichment analysis for biological process, molecular function, and cellular component terms among genes differentially methylated between PRRSV and PRRSV+FTO^{KO} groups. (b) KEGG pathway enrichment analysis of differentially

methylated transcripts between PRRSV and PRRSV + FTO^{KO} groups. Dot size corresponds to gene counts, and color indicates statistical significance (adjusted *p*-value).

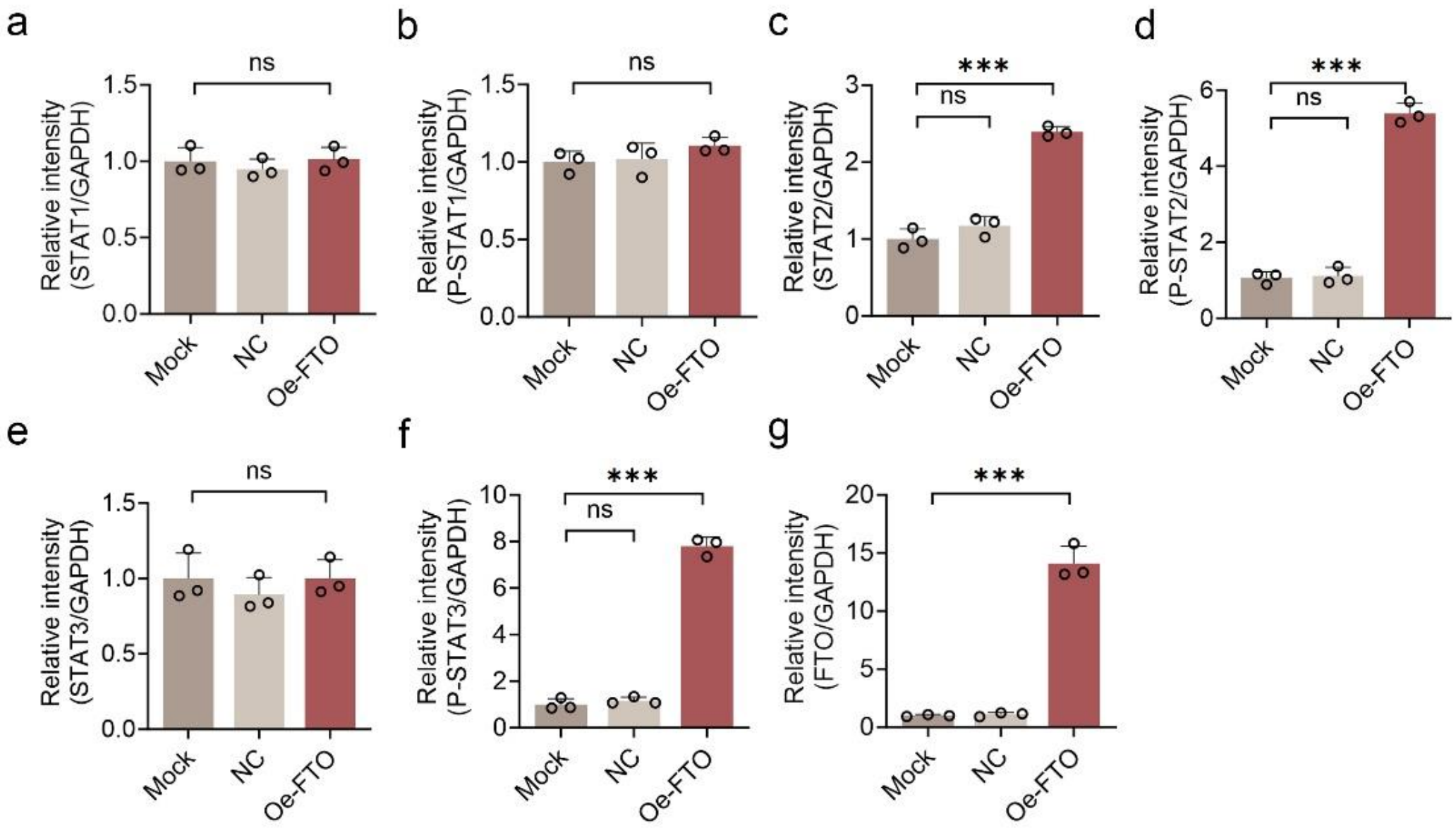

Fig. S19. rPAMs were transfected with Oe-NC and Oe-FTO. The quantification of Western blot intensity of STATs and p-STATs levels.

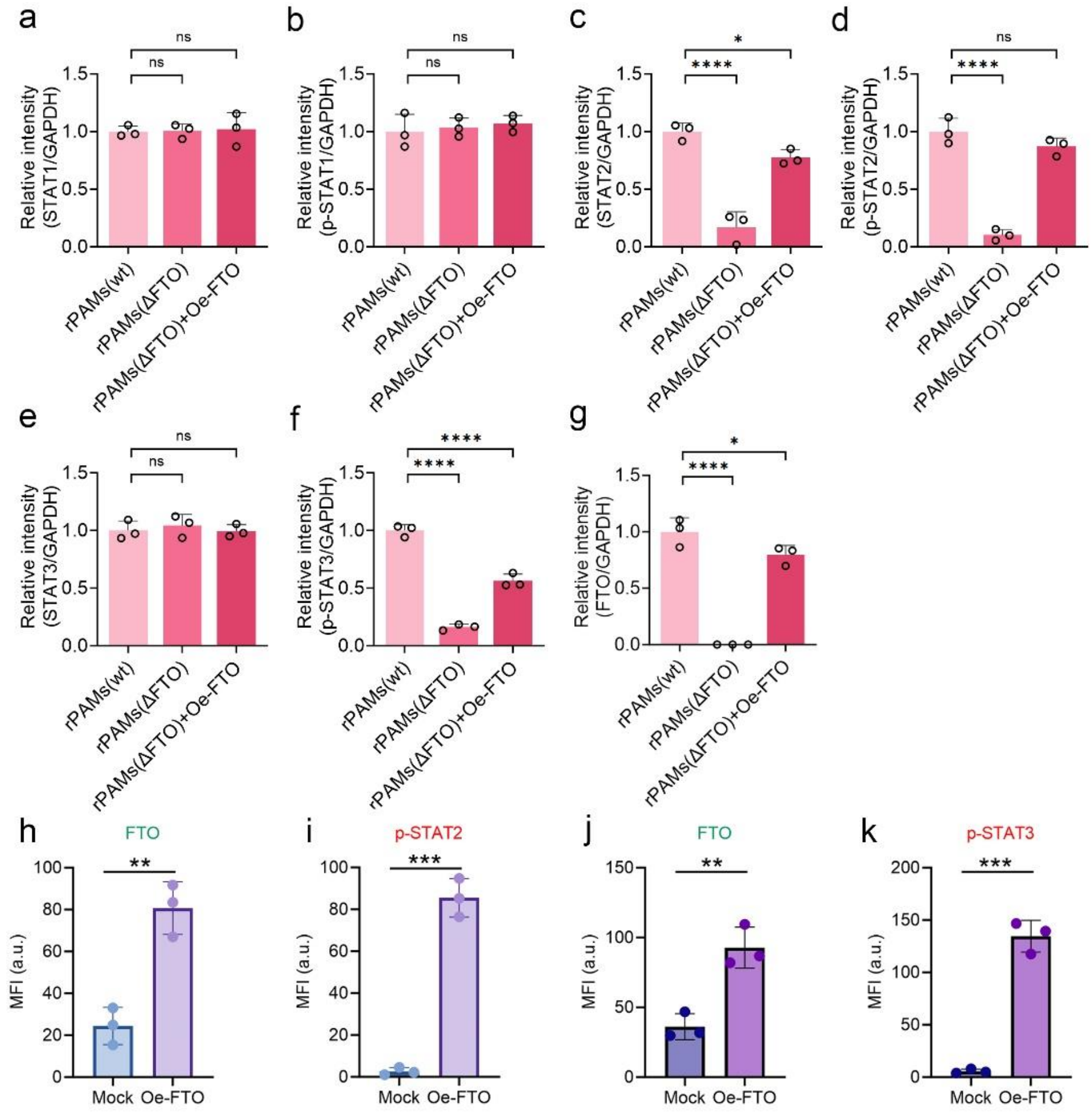

Fig. S20. (a-g) rPAMs (wild-type and FTO-knockout, ΔFTO) were transfected with or without the Oe-FTO plasmid. The quantification of Western blot intensity of STATs and p-STATs protein levels. (h, i) Quantification of the mean fluorescence intensity (MFI) of FTO (h) and p-STAT2 (i) from the corresponding immunofluorescence images. (j, k) Quantification of the MFI of FTO (j) and p-STAT3 (k) from the corresponding immunofluorescence images.

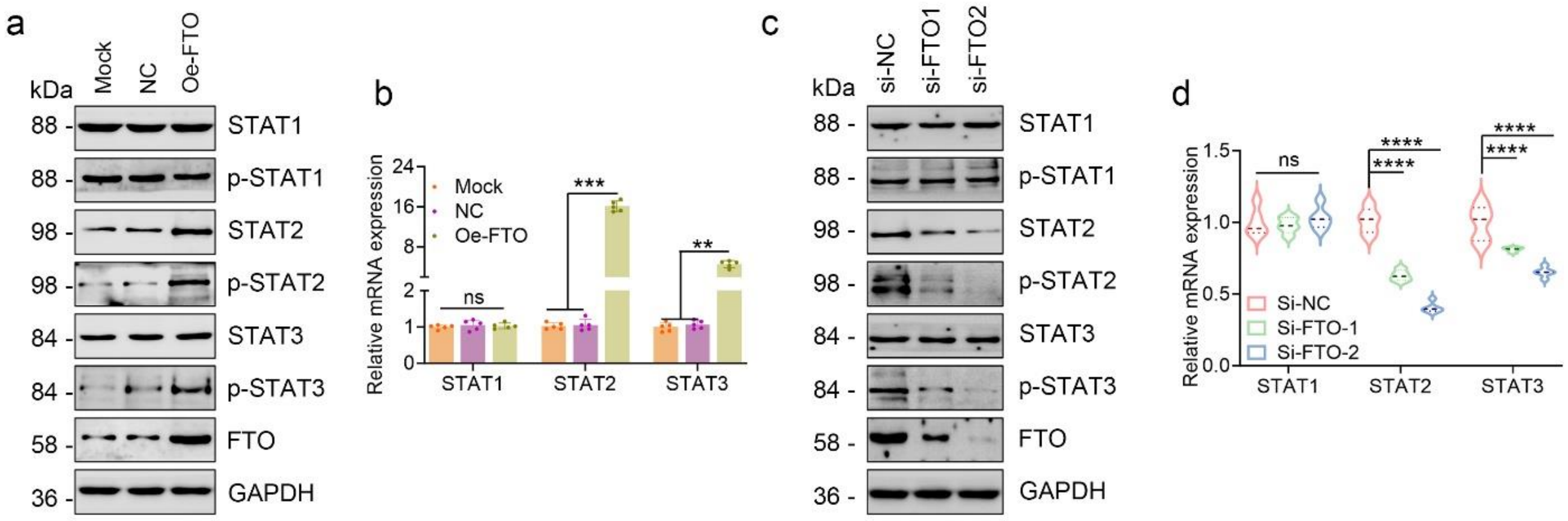

Fig. S21. (a, b) Transfection of Marc-145 cells with Oe-NC and Oe-FTO. (a) Western blot analysis of STATs and p-STATs protein expression levels. (b) qRT-PCR analysis of STATs mRNA expression levels. (c-d) Marc-145 cells were transfected with si-NC, si-FTO1, or si-FTO2. (c) Western blot analysis of STATs and p-STATs protein expression levels. (d) qRT-PCR analysis of STATs mRNA expression levels.

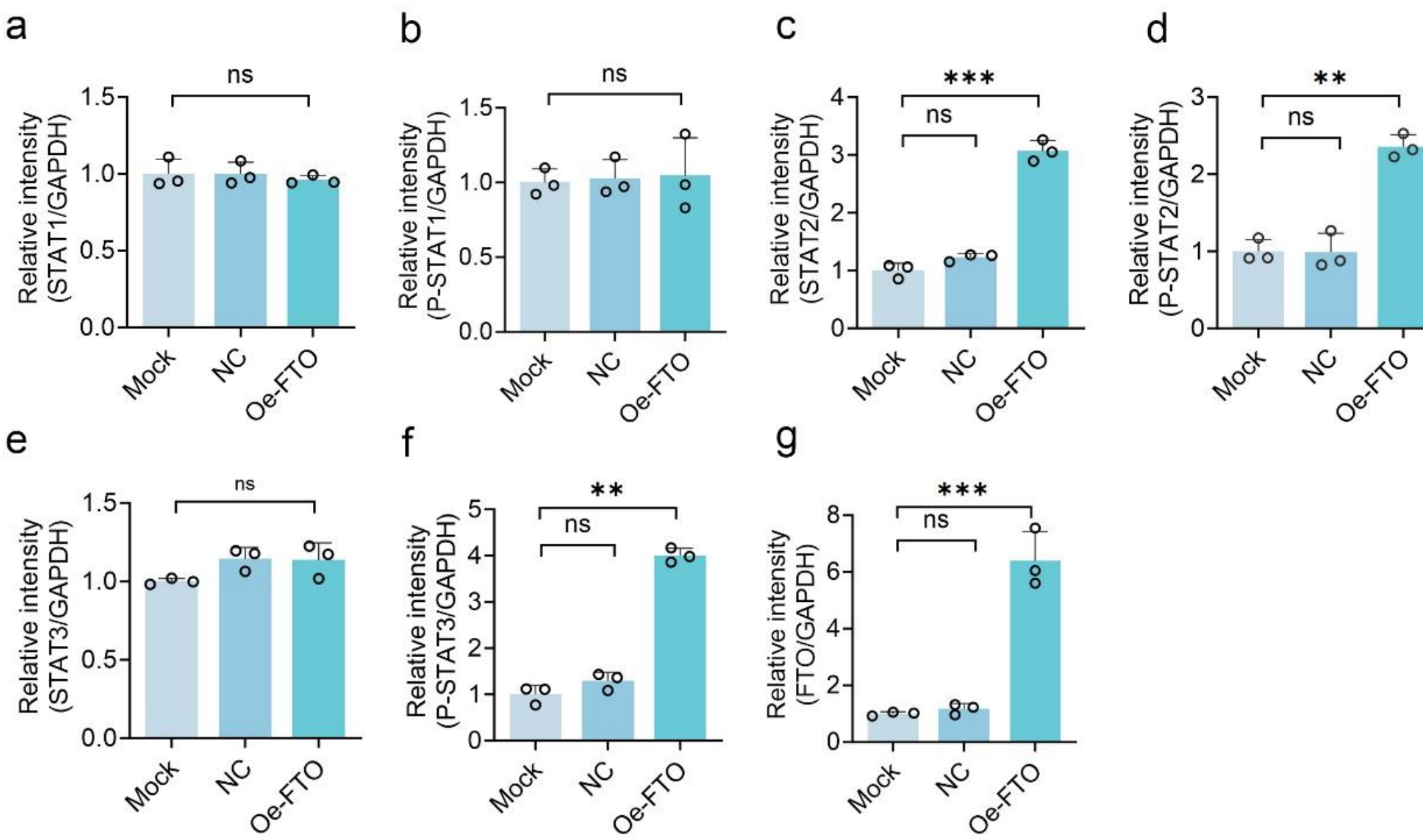

Fig. S22. Transfection of Marc-145 cells with Oe-NC and Oe-FTO. The quantification of Western blot intensity of STATs and p-STATs levels.

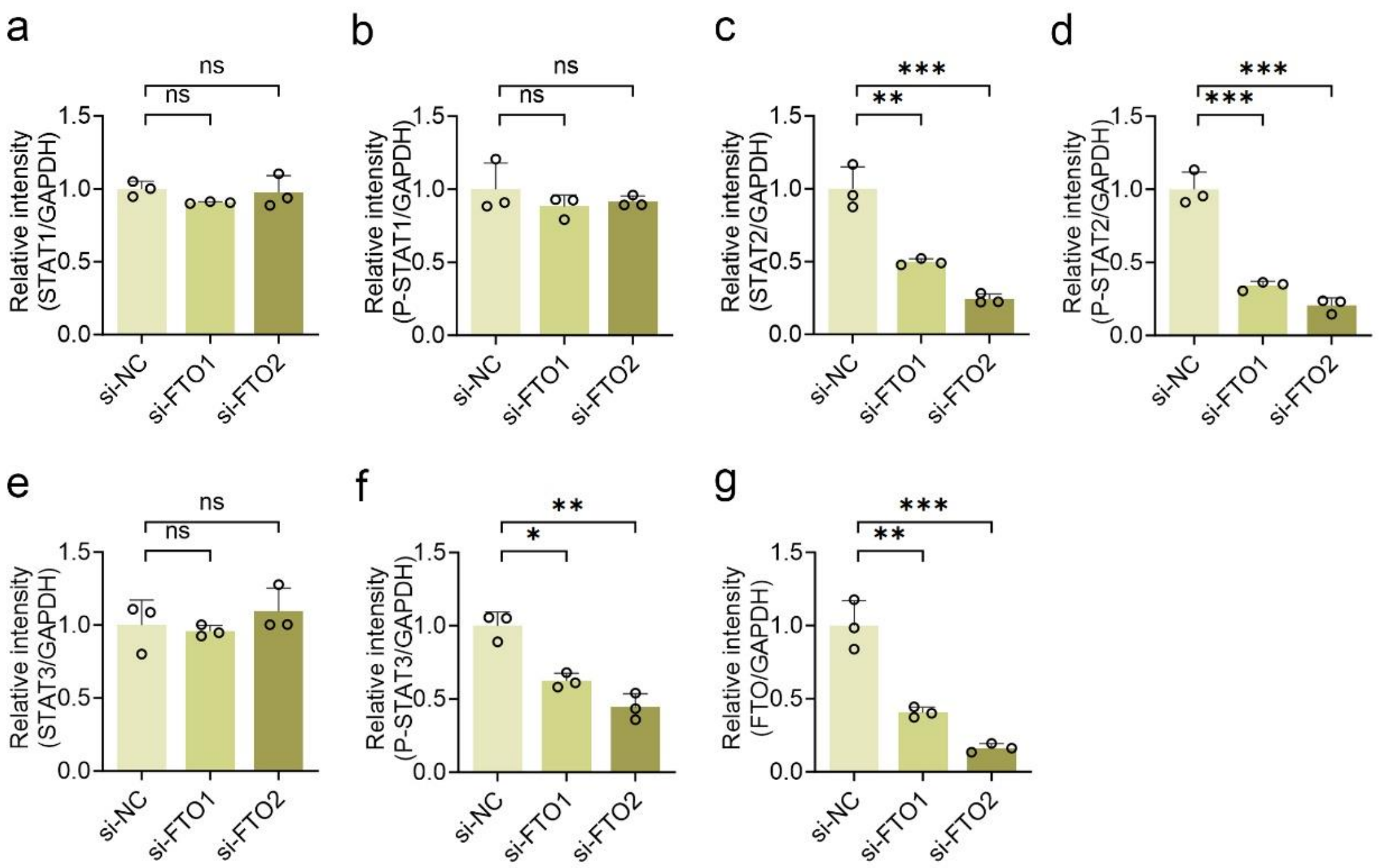

Fig. S23 Marc-145 cells were transfected with si-NC, si-FTO1, or si-FTO2. The quantification of Western blot intensity of STATs and p-STATs levels.

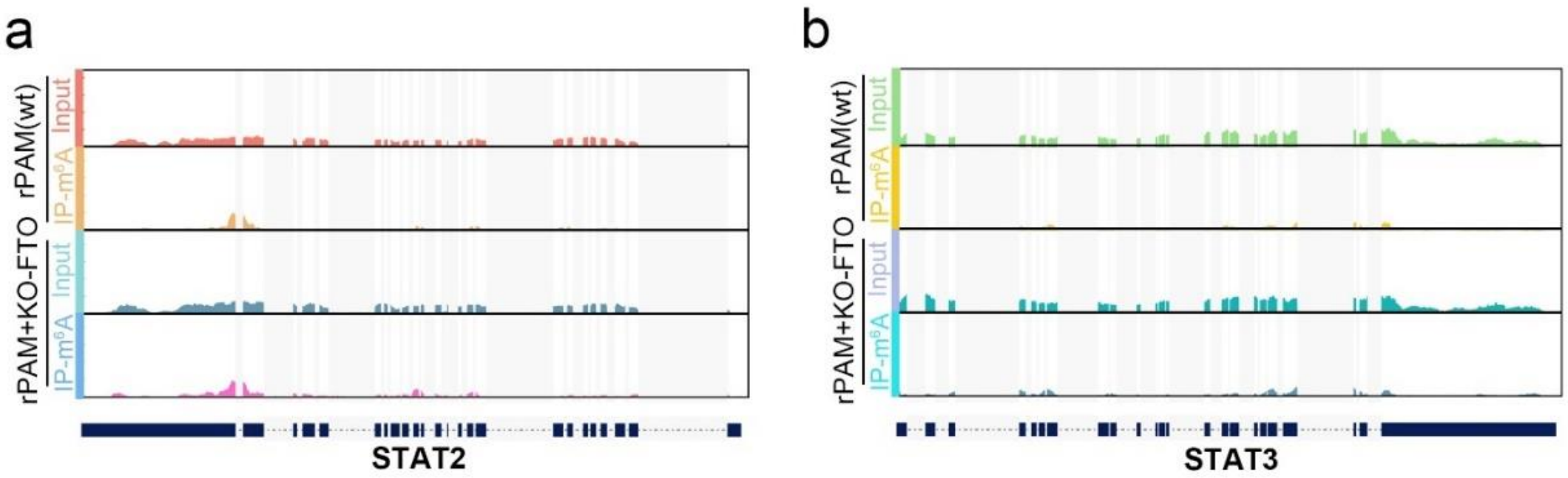

Fig. S24 IGV tracks of *m6A* peak distribution on STAT2 (a) and STAT3 (b) transcripts in rPAMs (wt) or rPAMs(ΔFTO).

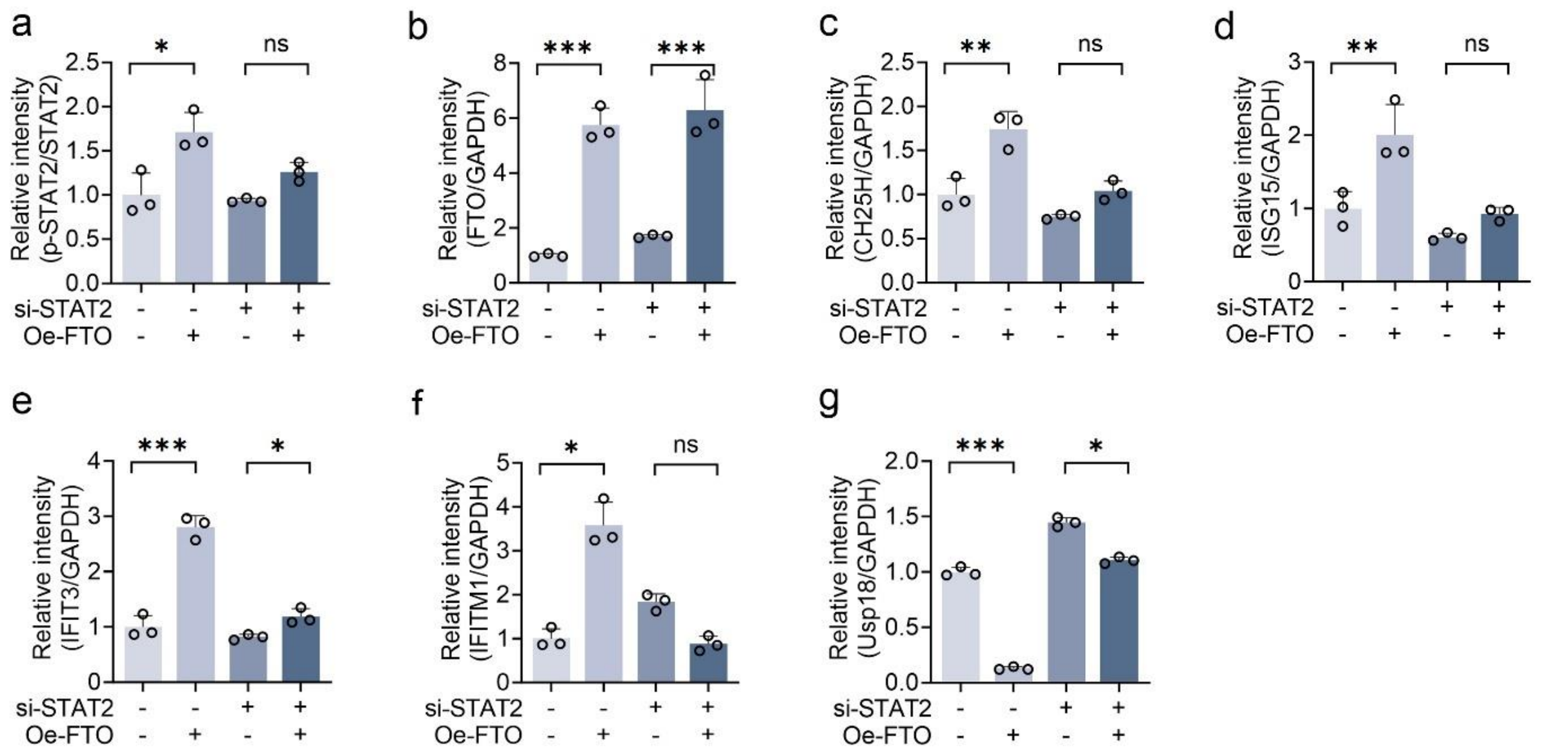

Fig. S25. rPAMs were transfected with Oe-NC or Oe-FTO in the presence or absence of siRNA targeting STAT2. Densitometric analysis of Western blot results shown in Figure 4n, including relative protein levels of (a) p-STAT2/STAT2, (b) FTO, (c) CH25H, (d) ISG15, (e) IFIT3, (f) IFITM1, and (g) USP18, normalized to GAPDH (or total STAT2 for p-STAT2). Data are presented as mean ± SD from n = 3 independent experiments. Statistical significance was determined by one-way ANOVA with post hoc tests: $*P \leq 0.05$, $**P \leq 0.01$, $***P \leq 0.001$; ns, not significant.

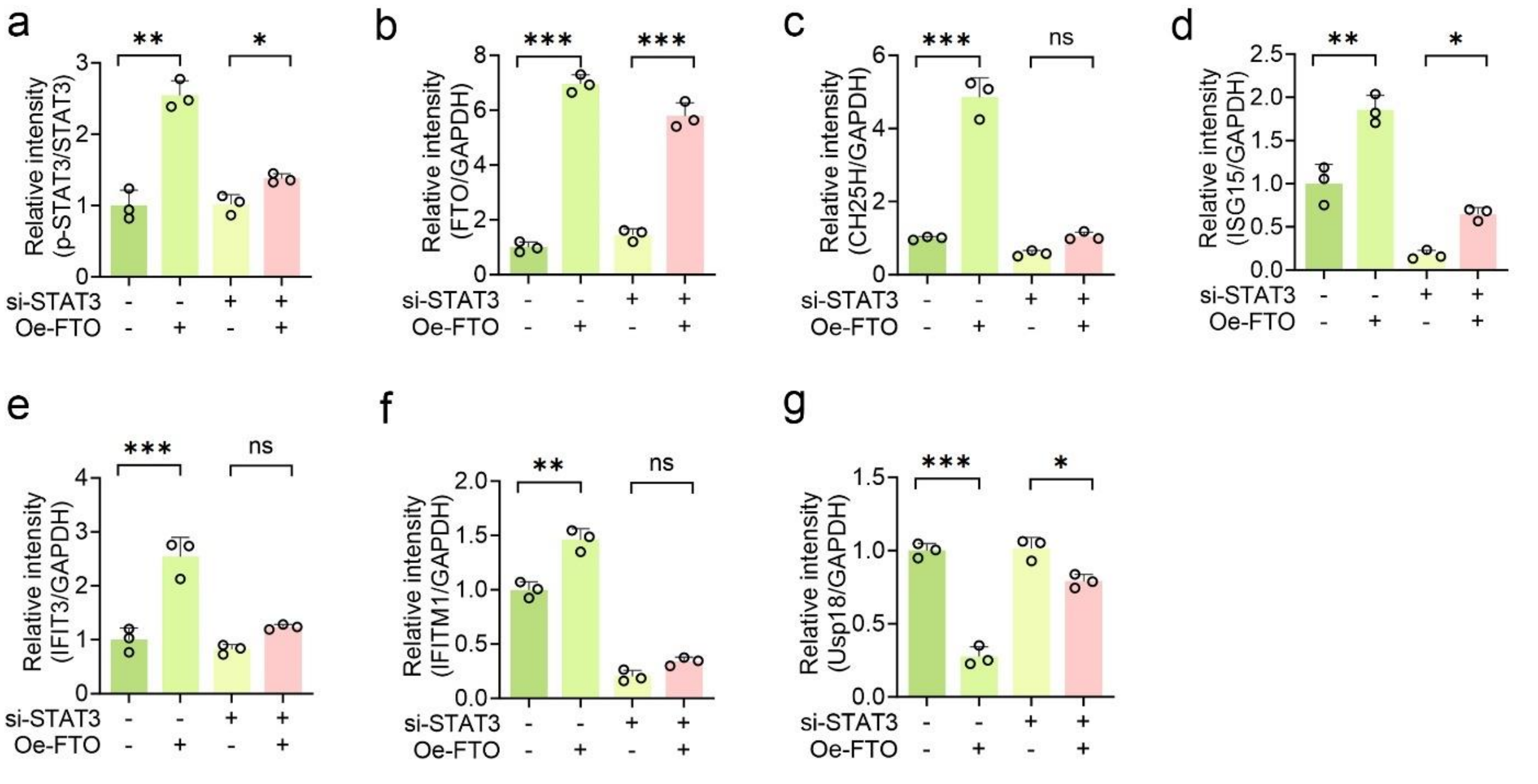

Fig. S26. rPAMS were transfected with Oe-NC or Oe-FTO in the presence or absence of siRNA targeting STAT3. Densitometric analysis of Western blot results shown in Figure 4q, including relative protein levels of (a) p-STAT3/STAT3, (b) FTO, (c) CH25H, (d) ISG15, (e) IFIT3, (f) IFITM1, and (g) USP18, normalized to GAPDH (or total STAT3 for p-STAT3). Data are presented as mean ± SD from n = 3 independent experiments. Statistical significance was

determined by one-way ANOVA with post hoc tests: *$P \leq 0.05$, **$P \leq 0.01$, ***$P \leq 0.001$*; ns, not significant.

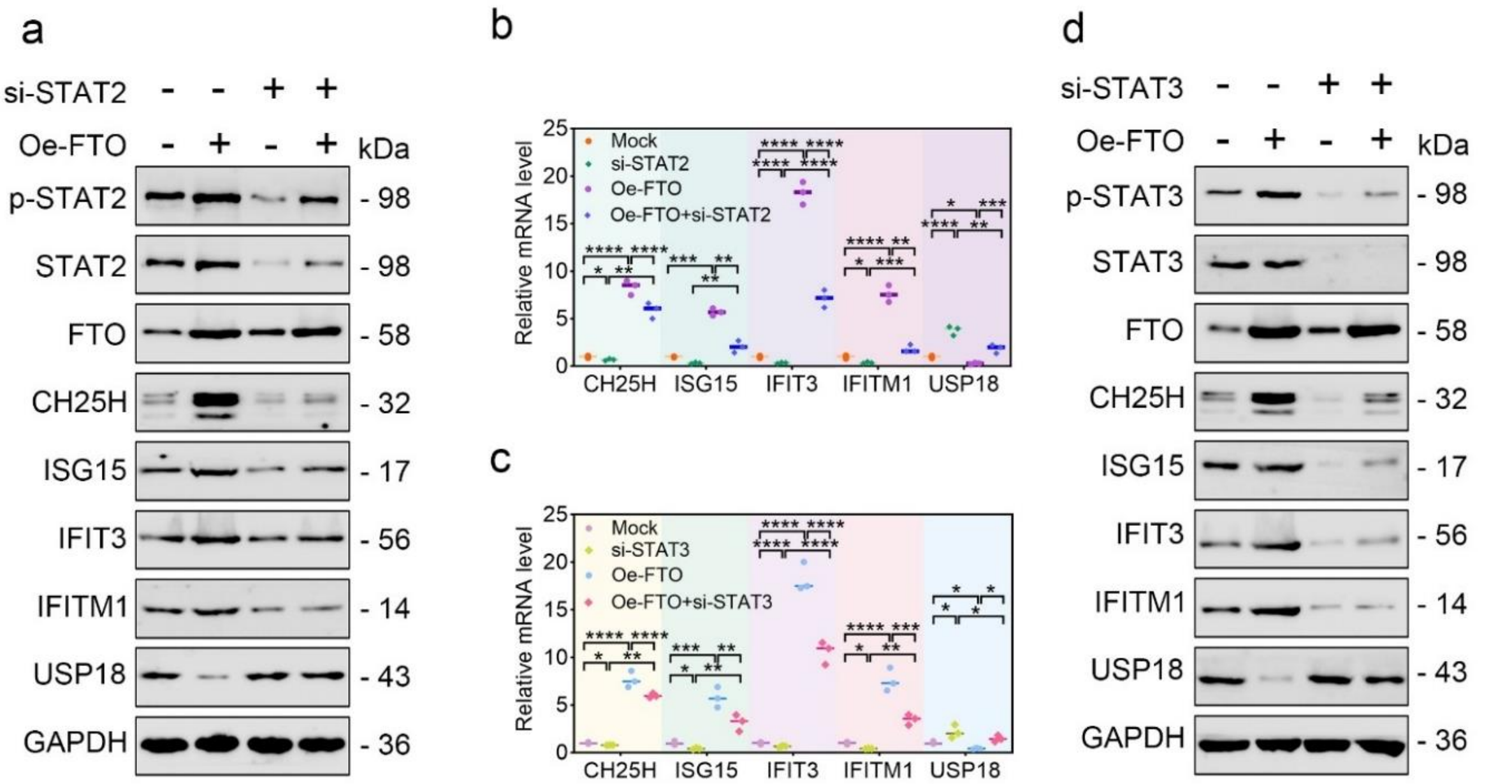

Fig. S27. Marc-145 cells were transfected with Oe-NC or Oe-FTO in the presence or absence of siRNA targeting STAT2 or STAT3. (a, d) Western blot analysis of phosphorylated STAT2/STAT3, total STAT2/STAT3, FTO, and ISG proteins (CH25H, ISG15, IFIT3, IFITM1, and USP18). GAPDH served as a loading control. (b, c) qRT-PCR analysis of ISG mRNA expression levels under the same conditions. Data are representative of three independent experiments, with (b, c) showing quantification from n = 5 biologically independent samples. Statistical significance was determined by one-way ANOVA with post hoc tests: *$P \leq 0.05$, **$P \leq 0.01$, ***$P \leq 0.001$, ****$P \leq 0.0001$*; ns, not significant.

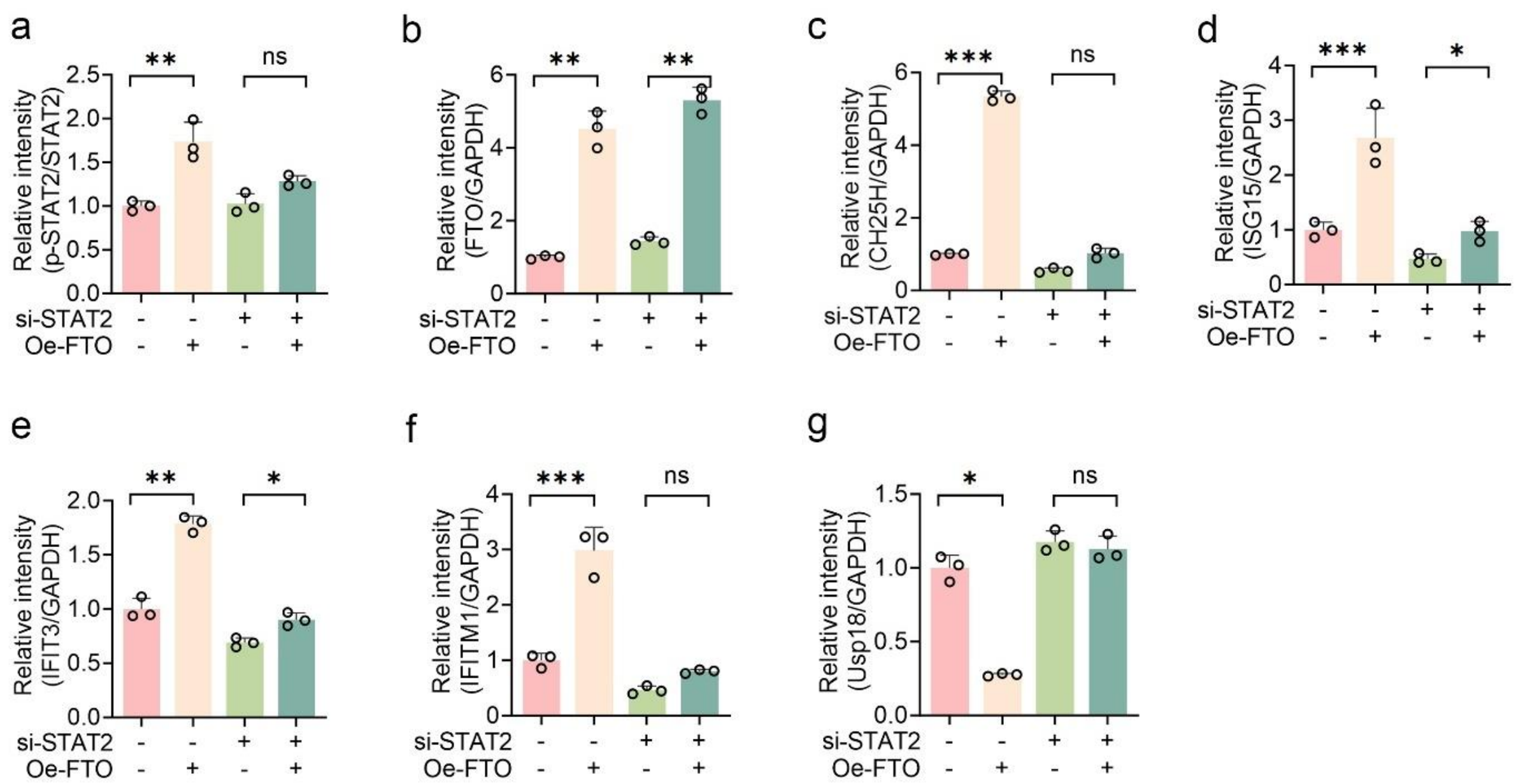

Fig. S28. Marc-145 cells were transfected with Oe-NC or Oe-FTO in the presence or absence of siRNA targeting STAT2. Densitometric analysis of Western blot results shown in Figure S27a, including relative protein levels of (a) p-STAT2/STAT2, (b) FTO, (c) CH25H, (d) ISG15, (e) IFIT3, (f) IFITM1, and (g) USP18, normalized to GAPDH (or total STAT2 for p-STAT2). Data are presented as mean ± SD from n = 3 independent experiments. Statistical significance was determined by one-way ANOVA with post hoc tests: *$P \leq 0.05$, **$P \leq 0.01$, ***$P \leq 0.001$; ns, not significant.

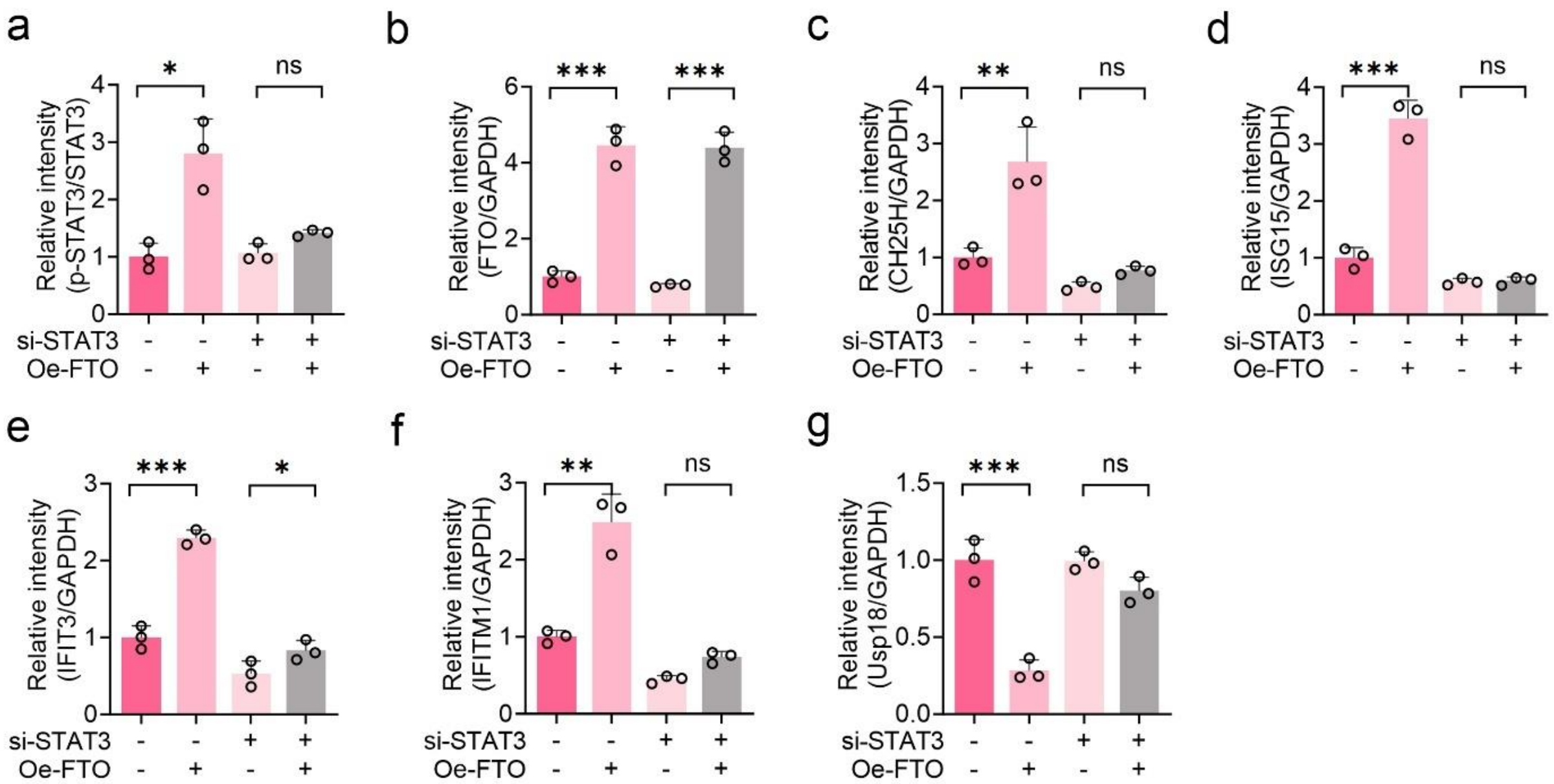

Fig. S29. Marc-145 cells were transfected with Oe-NC or Oe-FTO in the presence or absence of siRNA targeting STAT3. Densitometric analysis of Western blot results shown in Figure S27d, including relative protein levels of (a) p-STAT3/STAT3, (b) FTO, (c) CH25H, (d) ISG15, (e) IFIT3, (f) IFITM1, and (g) USP18, normalized to GAPDH (or total STAT3 for p-STAT3). Data are presented as mean ± SD from n = 3 independent experiments. Statistical significance was determined by one-way ANOVA with post hoc tests: *$P \leq 0.05$, **$P \leq 0.01$, ***$P \leq 0.001$; ns, not significant.

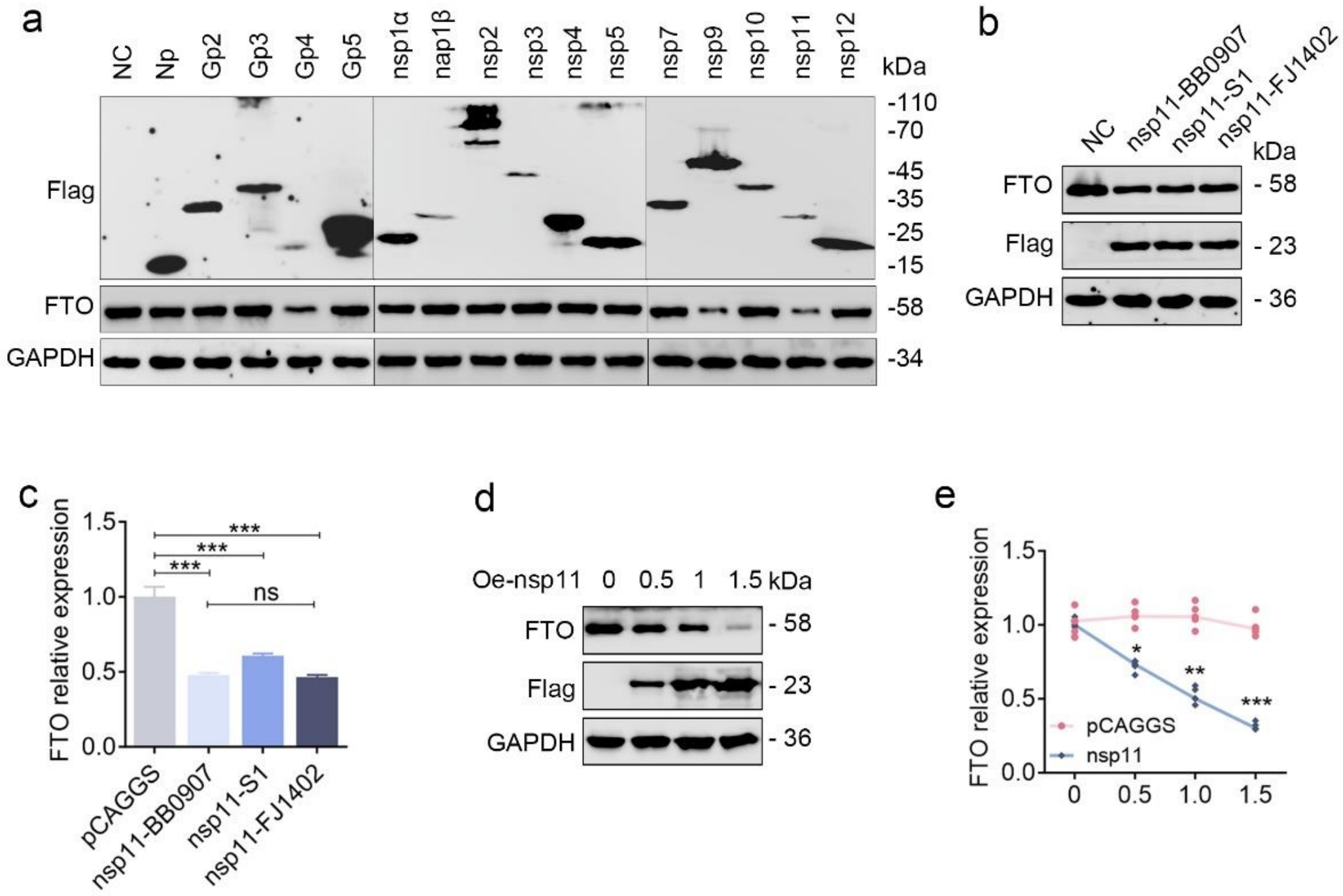

Fig. S30. (a) Western blot analysis of FTO protein levels in Marc-145 cells transfected with plasmids expressing various PRRSV structural (N, GP2–GP5) and nonstructural proteins (nsp1α–nsp12) for 36 h. GAPDH was used as a loading control. (b, c) Validation of nsp11-mediated FTO suppression across different viral strains. Marc-145 cells were transfected with nsp11 plasmids derived from PRRSV strains BB0907, S1, or FJ1402, followed by (b) Western blot and (c) qRT-PCR analysis of FTO expression (normalized to GAPDH; n = 3). (d, e) Dose-dependent effect of nsp11 on FTO expression. Marc-145 cells were transfected with increasing doses (0.5, 1.0, 1.5 μg) of nsp11 plasmid for 36 h, and FTO expression was examined by (d) Western blot and (e) qRT-PCR analysis (normalized to GAPDH; n = 3). Data are shown as mean ± SD. Statistical significance: *$p < 0.05$, **$p < 0.01$, ***$p < 0.001$; ns, not significant.

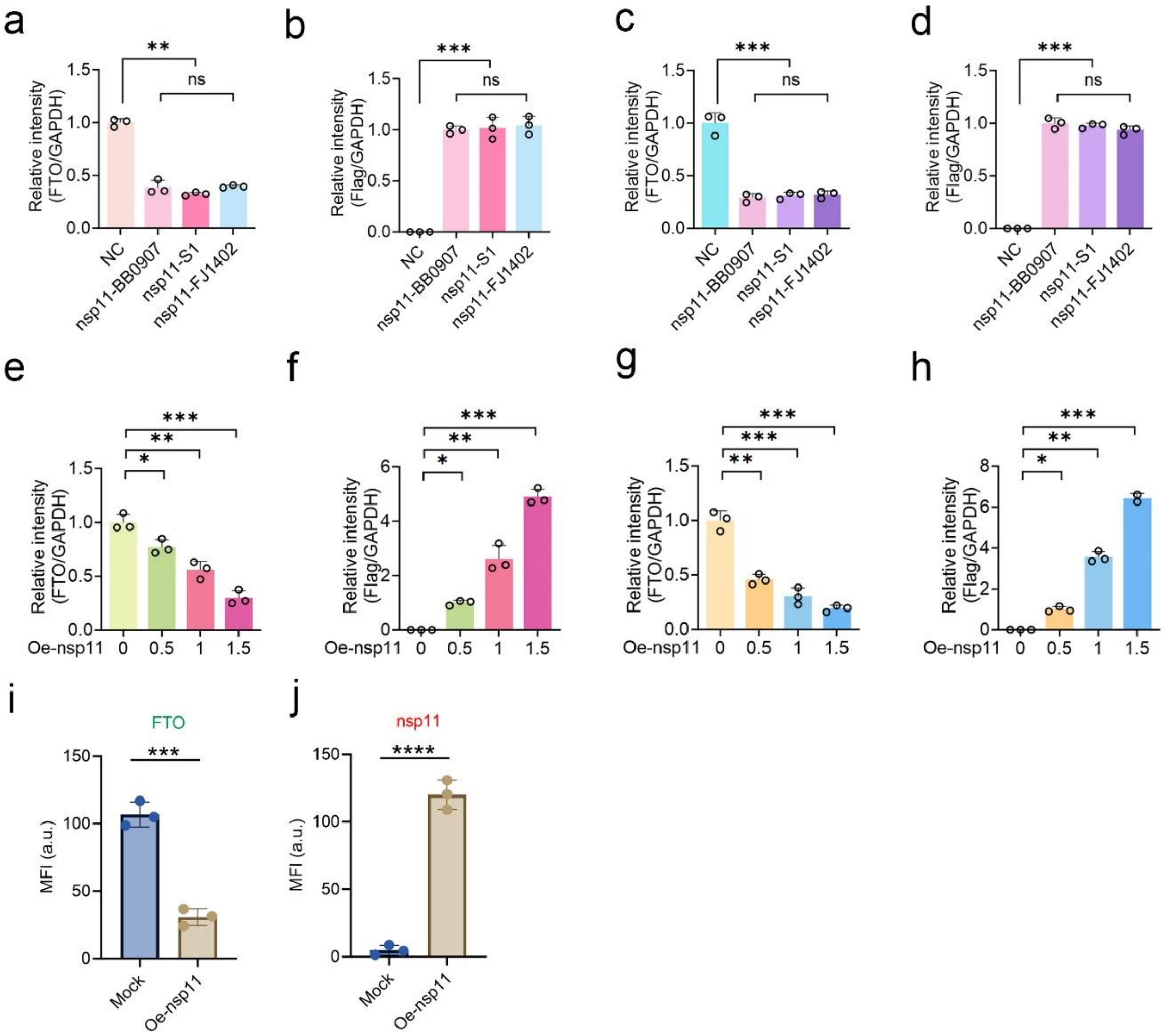

Fig. S31. (a, b) rPAMs were transfected with nsp11 plasmids derived from PRRSV strains BB0907, S1, or FJ1402 for 36 h. Densitometric quantifications of (a) FTO and (b) Flag protein expression normalized to GAPDH. (c, d) rPAMs were transfected with nsp11 plasmids derived from PRRSV strains BB0907, S1, or FJ1402 for 36 h. Densitometric quantifications of (c) FTO and (d) Flag protein expression normalized to GAPDH. rPAMs were transfected with increasing doses (0.5, 1.0, and 1.5 µg) of nsp11 plasmid for 36 h. Densitometric quantifications of (e) FTO and (f) Flag protein levels normalized to GAPDH. Marc-145 cells were transfected with the nsp11 plasmid at increasing doses (0.5, 1.0, and 1.5 µg) for 36 h. Densitometric quantifications of (g) FTO and (h) Flag protein levels normalized to GAPDH. (i, j) Quantification of the mean fluorescence intensity (MFI) of FTO (i) and nsp11 (j) in Mock and nsp11-overexpressing cells. Data represent mean ± SD (n = 3). Statistical significance: *$p < 0.05$, **$p < 0.01$, ***$p < 0.001$.
