# Supplementary material for: PRRSV suppresses FTO-dependent m6A demethylation to reprogram STAT signaling and innate immunity
Source: mBio. 2026 Aug 13;17(9):e00843-26. doi: 10.1128/mbio.00843-26 (PMC13556206; doi:10.1128/mbio.00843-26)
Supplement: Supplementary file 2 [file mbio.00843-26-s0002.pdf]

Supporting Information

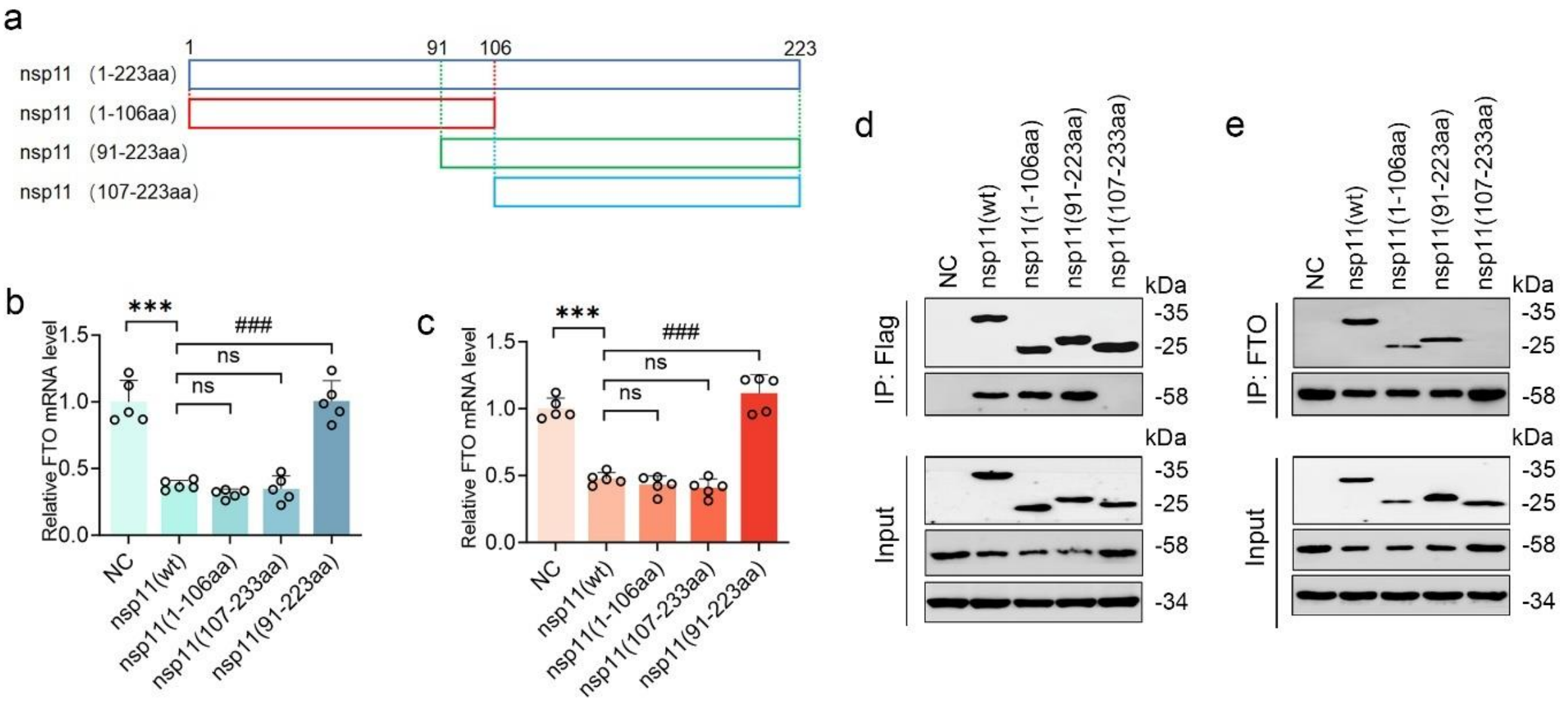

Fig. S32. (a) Schematic representation of nsp11 truncation mutants. (b, c) Marc-145 cells were transfected with plasmids expressing nsp11(wt) or the indicated truncated mutants, and FTO mRNA levels were quantified by qRT-PCR (normalized to GAPDH; n = 3). (d, e) Co-immunoprecipitation (Co-IP) analysis of the interaction between nsp11 and FTO in Marc-145 cells. (d) Immunoblotting and (e) Co-IP of FTO using an anti-FTO antibody, followed by immunoblotting for Flag. Input samples were used as loading controls.

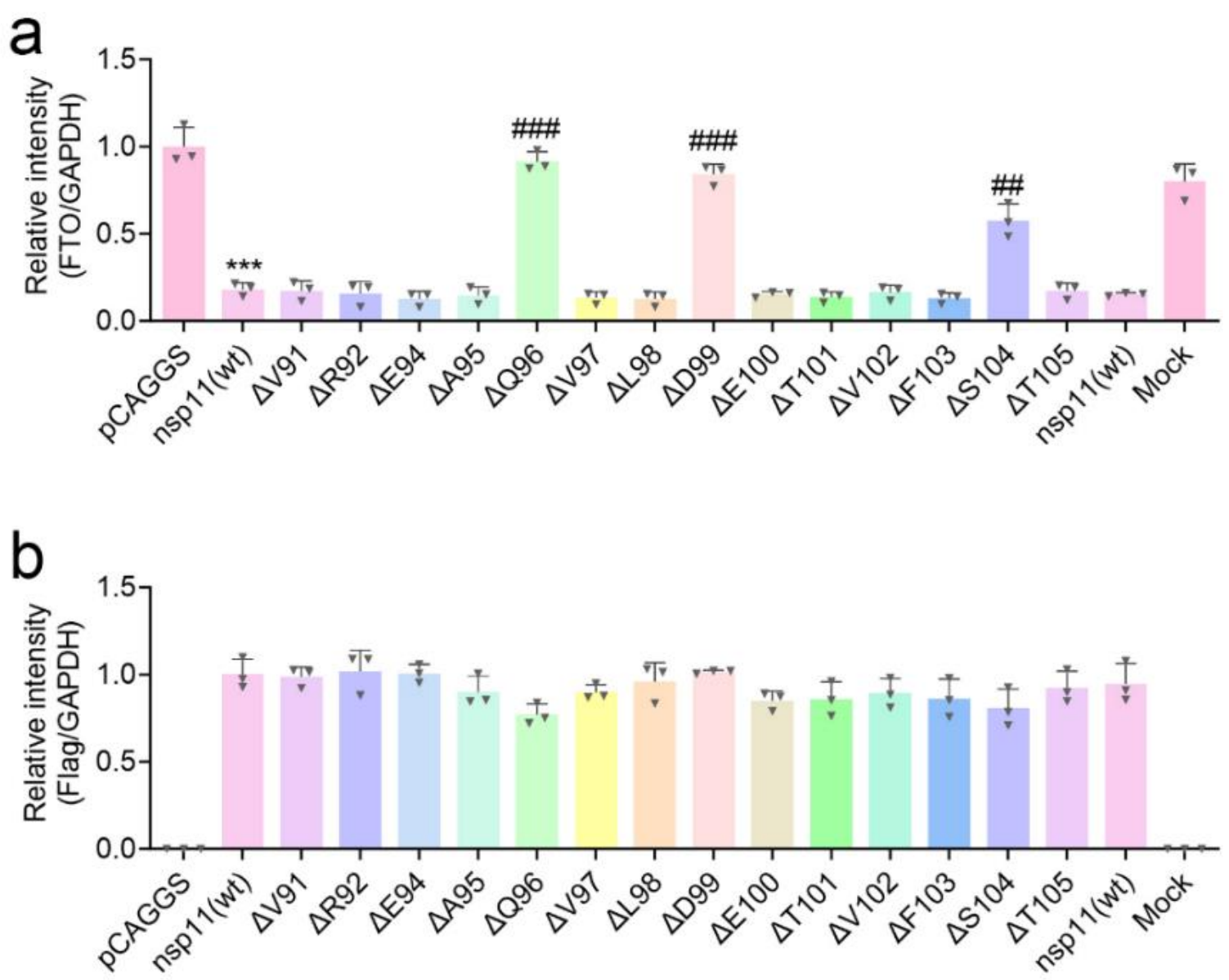

Fig. S33. Quantification of FTO and nsp11 protein expression in rPAMs transfected with wild-type (wt) or site-directed mutant nsp11 plasmids. (a) Densitometric analysis of Western blot results showing relative FTO protein levels normalized to GAPDH. (b) Densitometric analysis of Flag-tagged nsp11 protein levels normalized to GAPDH. Data are presented as mean

± SD (n = 3). Statistical significance was assessed by one-way ANOVA with post hoc testing (**p < 0.05, **p < 0.01, ***p < 0.001* vs. Mock; *#p < 0.05, ##p < 0.01, ###p < 0.001* vs. nsp11(wt)).

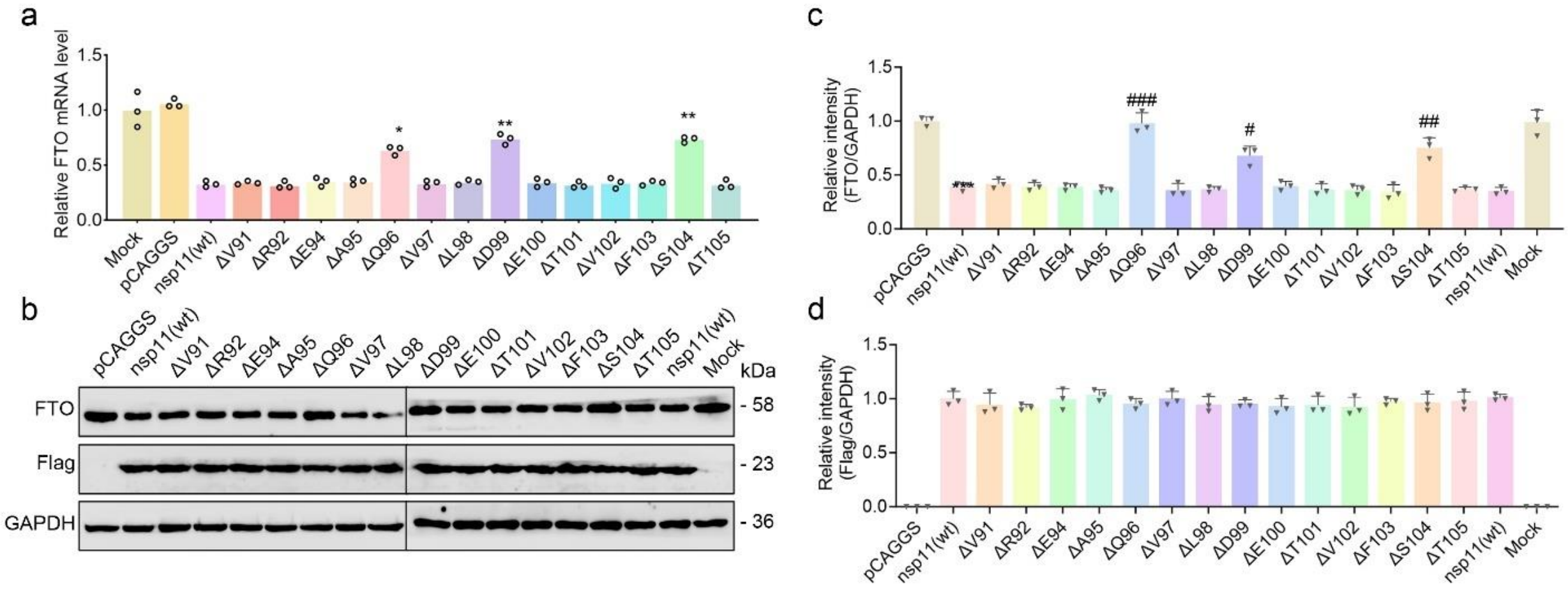

Fig. S34. Analysis of FTO expression in Marc-145 cells transfected with wild-type (wt) or site-directed mutant nsp11 expression plasmids. (a) qRT-PCR analysis of FTO mRNA levels. (b) Western blot analysis of FTO and Flag-tagged nsp11 protein expression. (c, d) Quantification of FTO (c) and Flag (d) protein levels normalized to GAPDH. Data are shown as mean ± SD (n = 3). Statistical significance was assessed by one-way ANOVA with post hoc testing *(*p < 0.05, **p < 0.01* vs. Mock; *#p < 0.05, ##p < 0.01, ###p < 0.001* vs. nsp11(wt)).

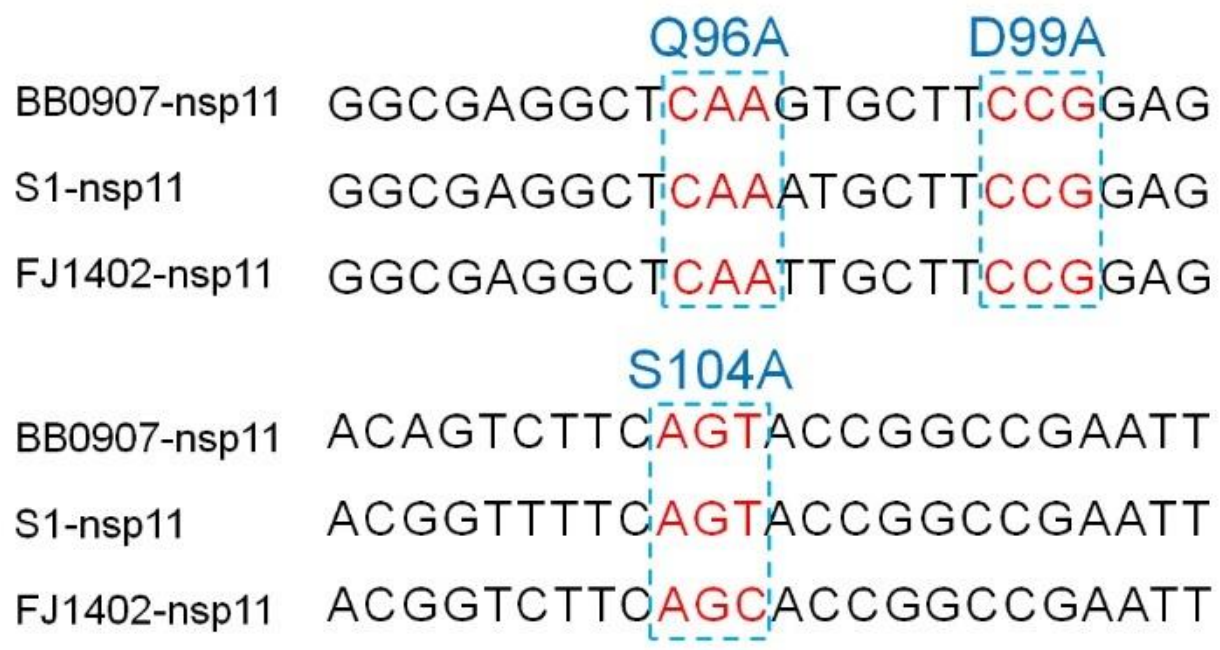

Fig. S35. Sequence alignment of nsp11 from three PRRSV strains (BB0907, S1, and FJ1402). The conserved glycine-rich region containing residues Q96, D99, and S104 is highlighted, with the corresponding alanine substitution sites (Q96A, D99A, and S104A) indicated.

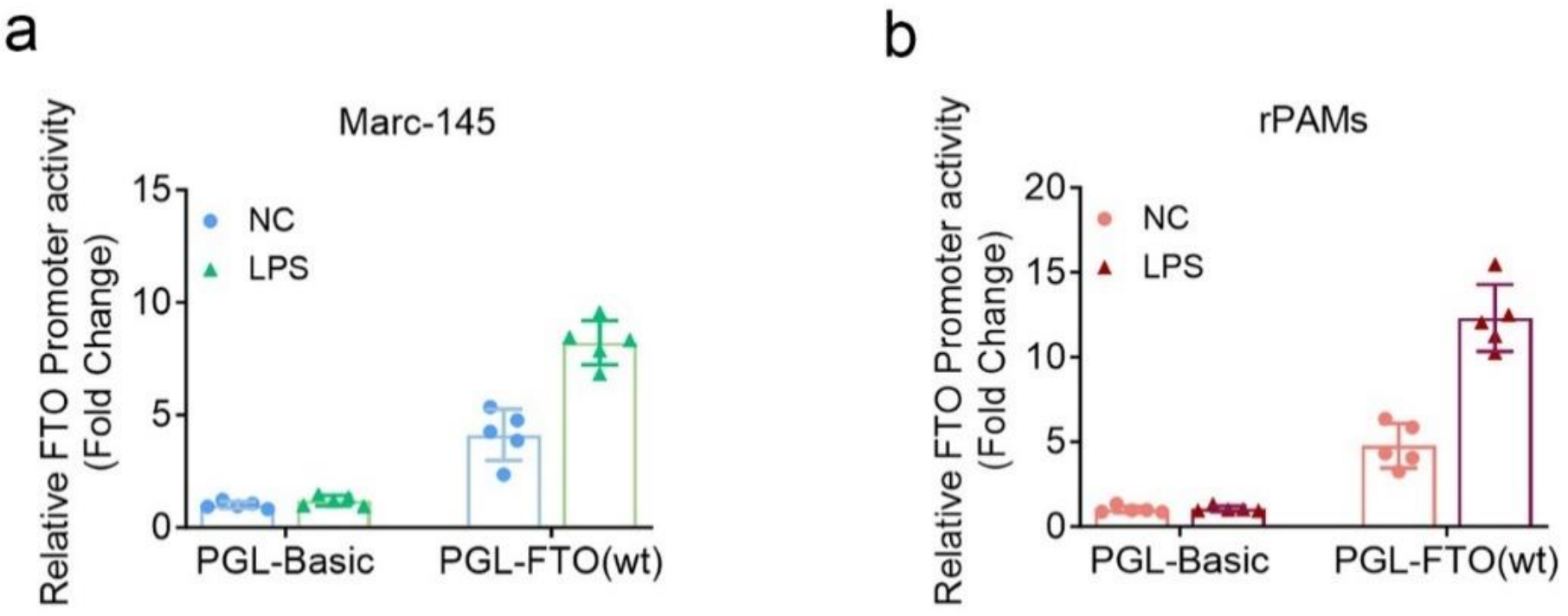

Fig. S36. Validation of FTO promoter activity using a dual-luciferase reporter system. Marc-145 cells (a) and rPAMs (b) were transfected with wild-type FTO promoter (pGL-FTO wt) or empty vector (pGL-Basic), followed by dual-luciferase reporter assay with or without LPS treatment. Promoter activity is shown as fold change relative to controls. Data represent mean ± SD from five independent experiments (n = 5).

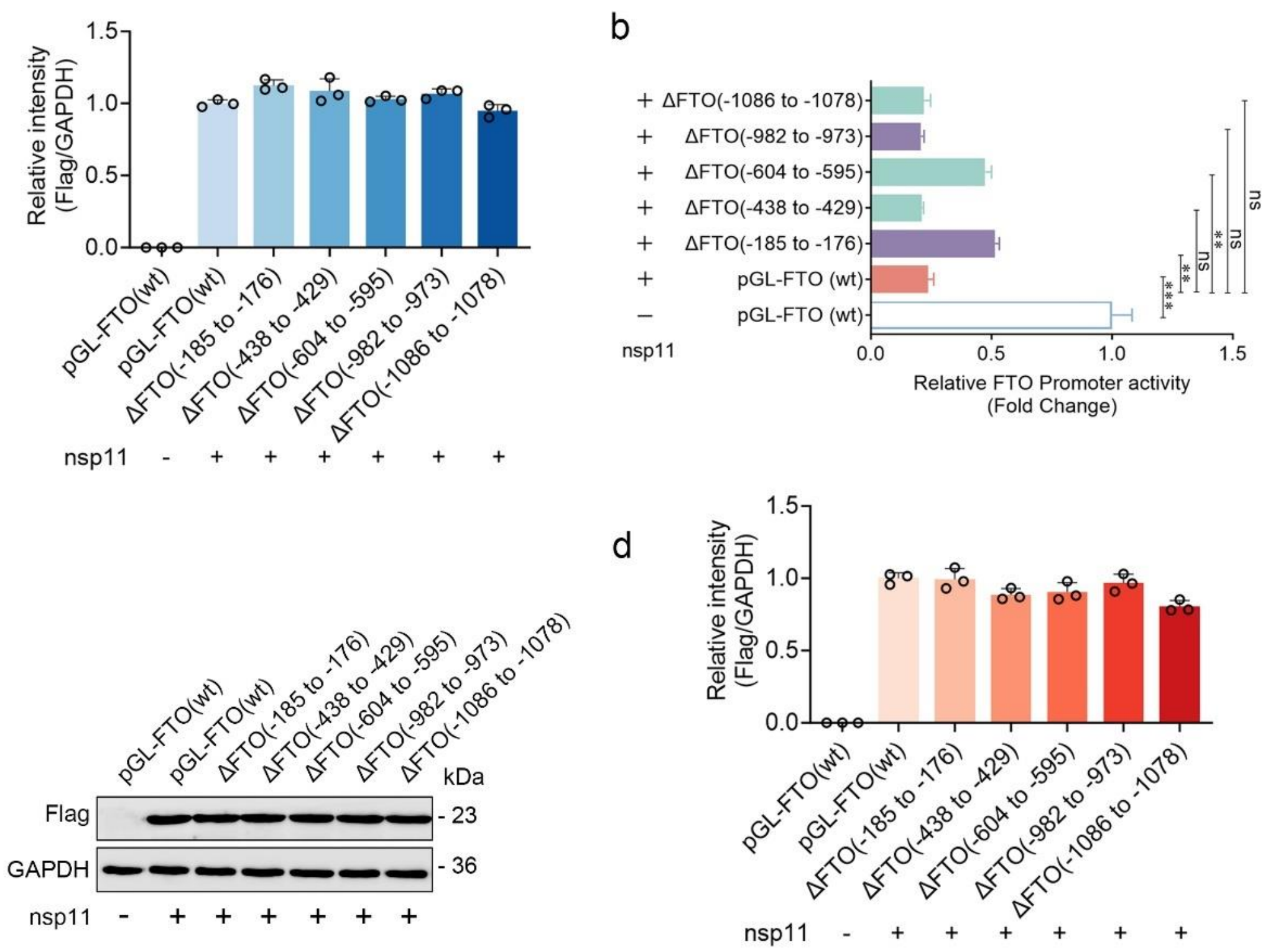

Fig. S37. (a) rPAMs were transfected with either mutant FTO promoter constructs or the pGL3 empty vector together with Oe-nsp11. Densitometric quantification of Flag protein levels normalized to GAPDH. (b) Dual-luciferase reporter assay showing relative FTO promoter activity in Marc-145 cells transfected with wild-type (pGL-FTO wt) or truncated mutant FTO promoter constructs together with Oe-nsp11. (c) Western blot analysis of nsp11 expression (Flag-tag) in the same experimental setting. (d) Quantification of Flag protein levels from (c).

Data represent mean ± SD from three independent experiments (n = 3). **P ≤ 0.05, **P ≤ 0.01, ***P ≤ 0.001*; ns, not significant.

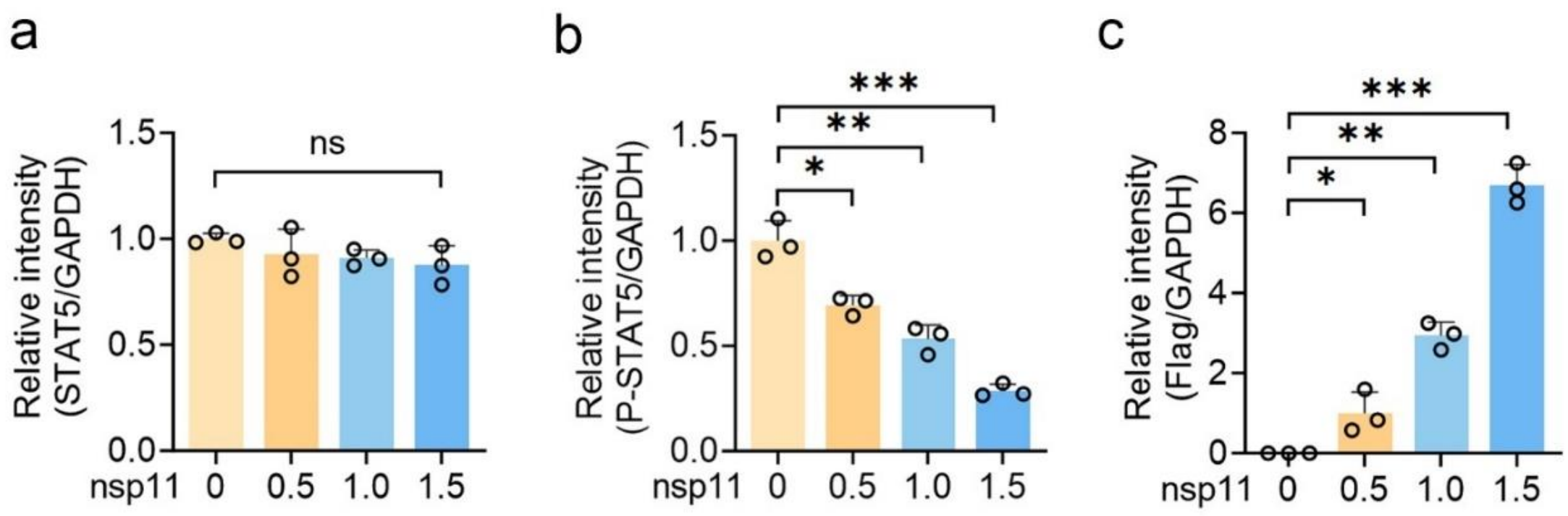

Fig. S38. rPAMs were transfected with increasing doses (0.5, 1.0, 1.5 μg) of nsp11 plasmid for 36 h. Relative protein levels of (a) STAT5, (b) p-STAT5, and (c) Flag (nsp11 expression control) were quantified by densitometry and normalized to GAPDH. Data represent mean ± SD from three independent experiments (n = 3). **P ≤ 0.05, **P ≤ 0.01, ***P ≤ 0.001*; ns, not significant.

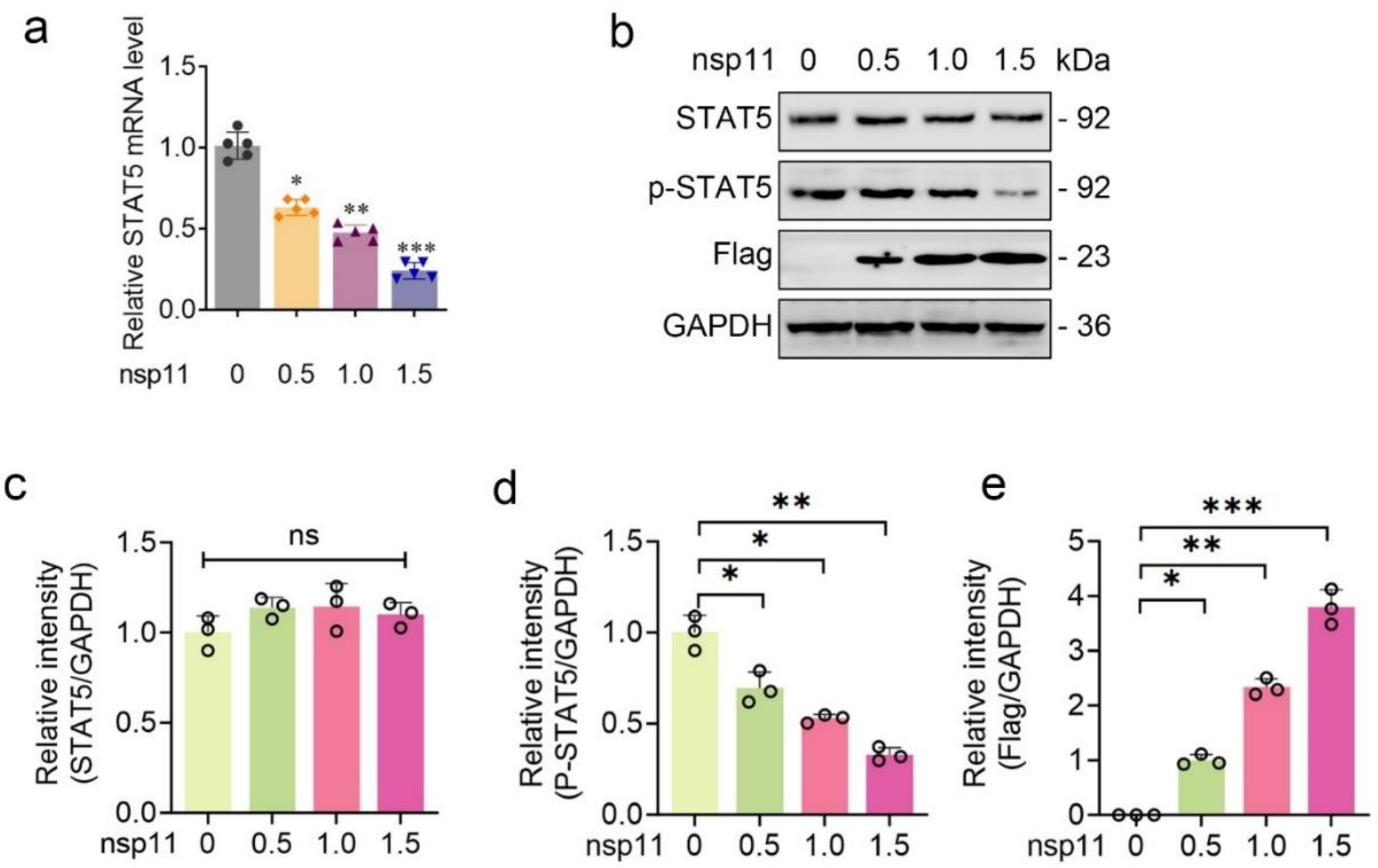

Fig. S39. Marc-145 cells were transfected with increasing doses (0.5, 1.0, 1.5 μg) of nsp11 plasmid for 36 h. (a) qRT-PCR analysis of STAT5 mRNA expression levels. (b) Western blot analysis of STAT5 and p-STAT5 protein expression levels. (c–e) Quantification of protein band intensities for STAT5 (c), p-STAT5 (d), and Flag (e), normalized to GAPDH. Data represent mean ± SD from three independent experiments (n = 3). **P ≤ 0.05, **P ≤ 0.01, ***P ≤ 0.001*; ns, not significant.

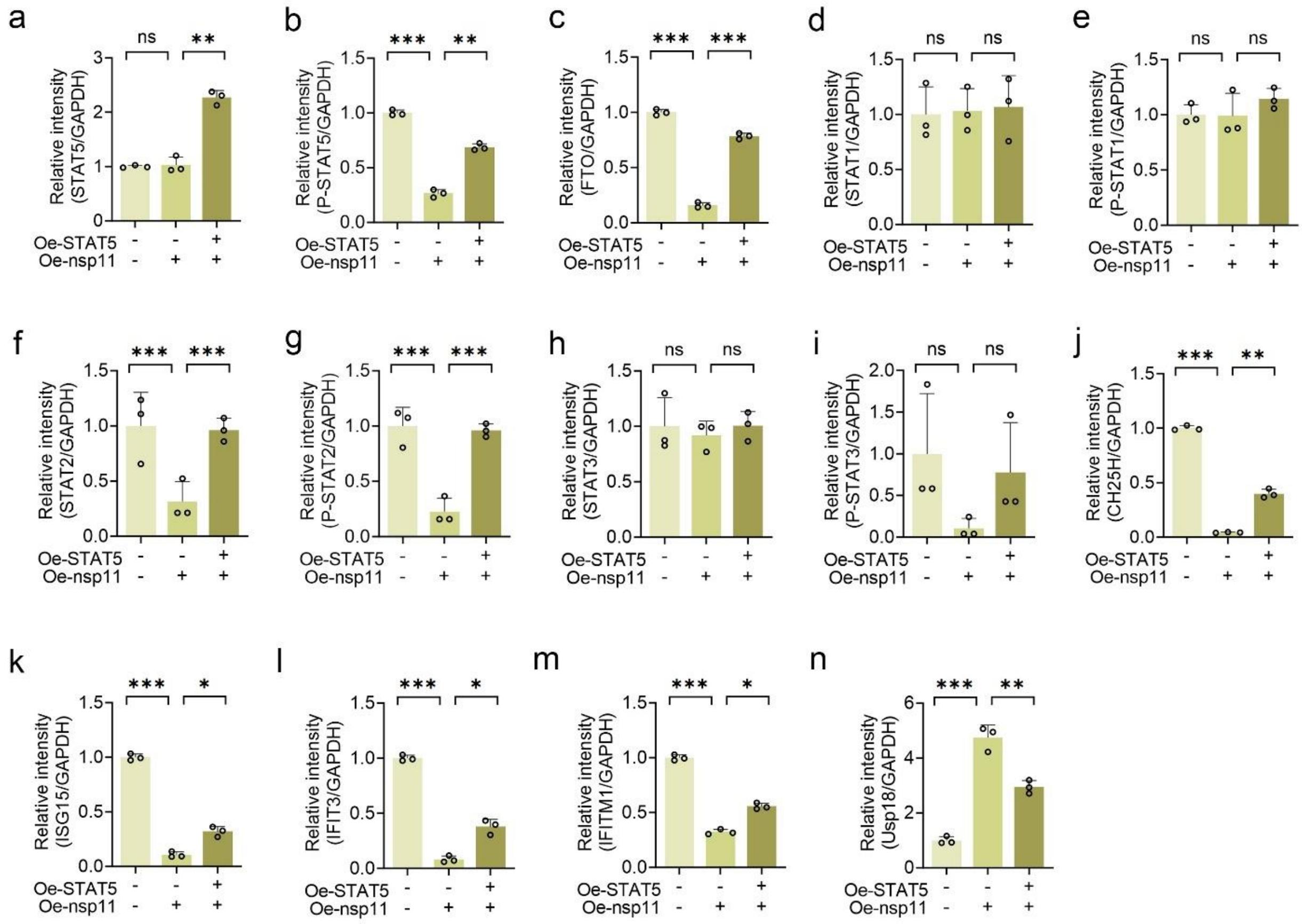

Fig. S40. Quantification of protein results from rPAMs transfected with Oe-nsp11 in the presence or absence of Oe-STAT5. Relative protein expression levels of (a–c) STAT5, p-STAT5, and FTO; (d, e) STAT1 and p-STAT1; (f, g) STAT2 and p-STAT2; (h, i) STAT3 and p-STAT3; (j–n) CH25H, ISG15, IFIT3, IFITM1, and USP18 were normalized to GAPDH. Data represent mean ± SD from three independent experiments (n = 3). $*P \leq 0.05$, $**P \leq 0.01$, $***P \leq 0.001$. ns, not significant.

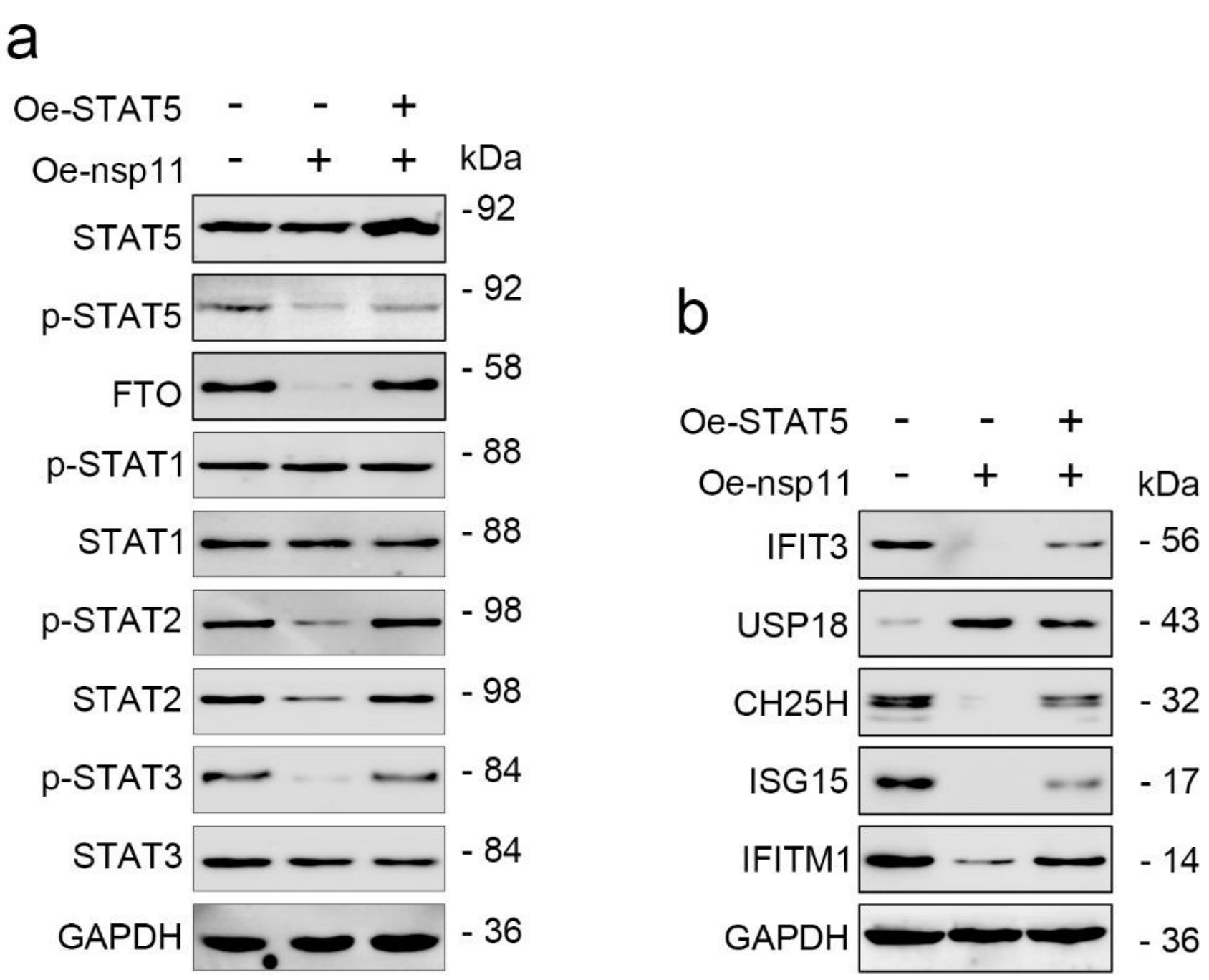

Fig. S41. Marc-145 cells were transfected with Oe-nsp11 in the presence or absence of Oe-STAT5. (a) Western blot analysis of FTO, STAT family proteins, and their phosphorylated forms (p-STAT1, p-STAT2, p-STAT3, and p-STAT5). (b) Western blot analysis of representative interferon-stimulated genes (ISGs: IFIT3, ISG15, IFITM1) and interferon negative regulators (USP18, CH25H). GAPDH was used as a loading control. Data are representative of three independent experiments.

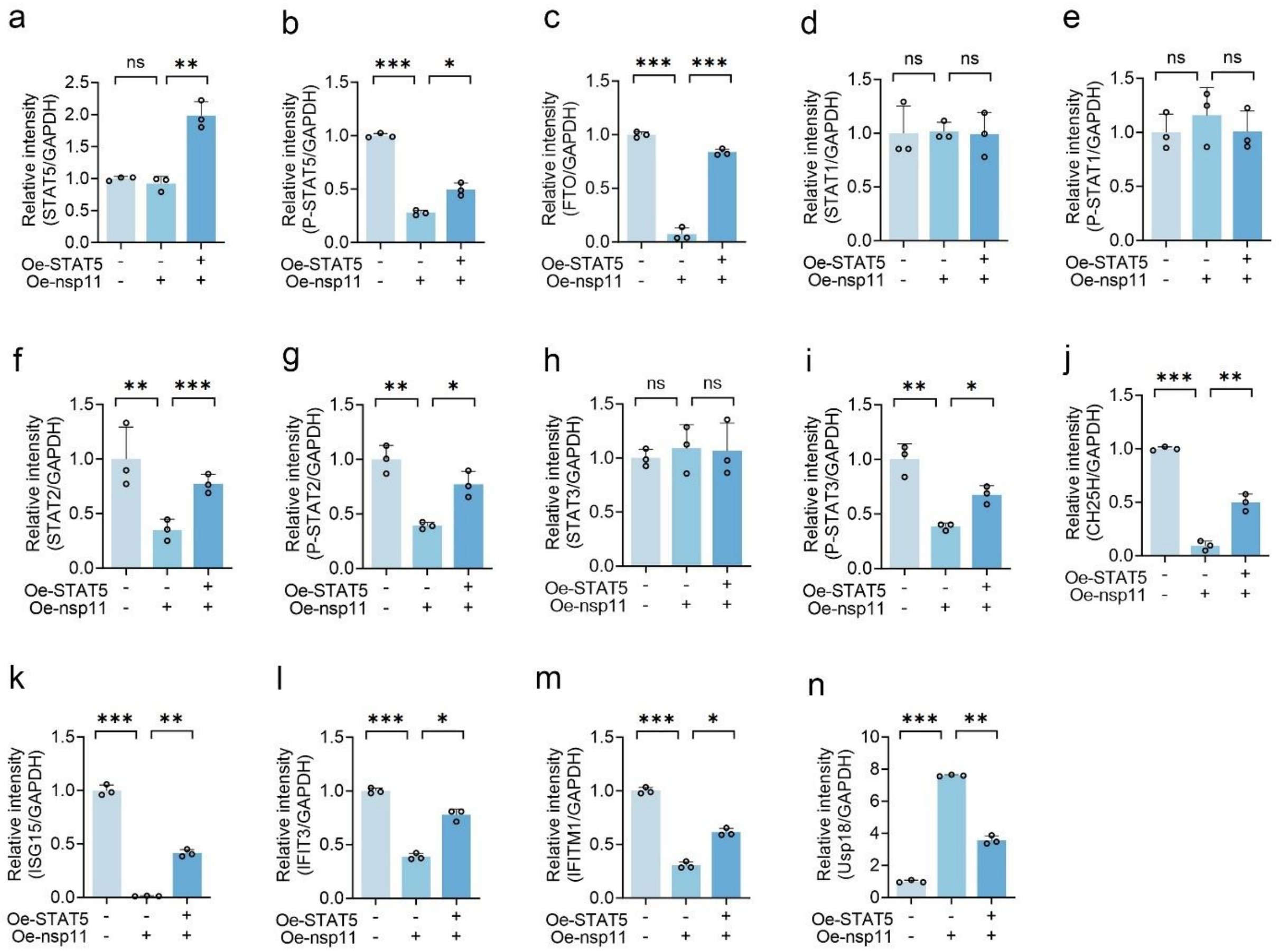

Fig. S42. Quantification of protein from Marc-145 cells transfected with Oe-nsp11 in the presence or absence of Oe-STAT5. Relative protein levels of (a–c) STAT5, p-STAT5, and FTO; (d, e) STAT1 and p-STAT1; (f, g) STAT2 and p-STAT2; (h, i) STAT3 and p-STAT3; (j–n) CH25H, ISG15, IFIT3, IFITM1, and USP18 were normalized to GAPDH. Data represent mean ± SD from three independent experiments (n = 3). *$*P \leq 0.05$, $**P \leq 0.01$, $***P \leq 0.001$*. ns, not significant.

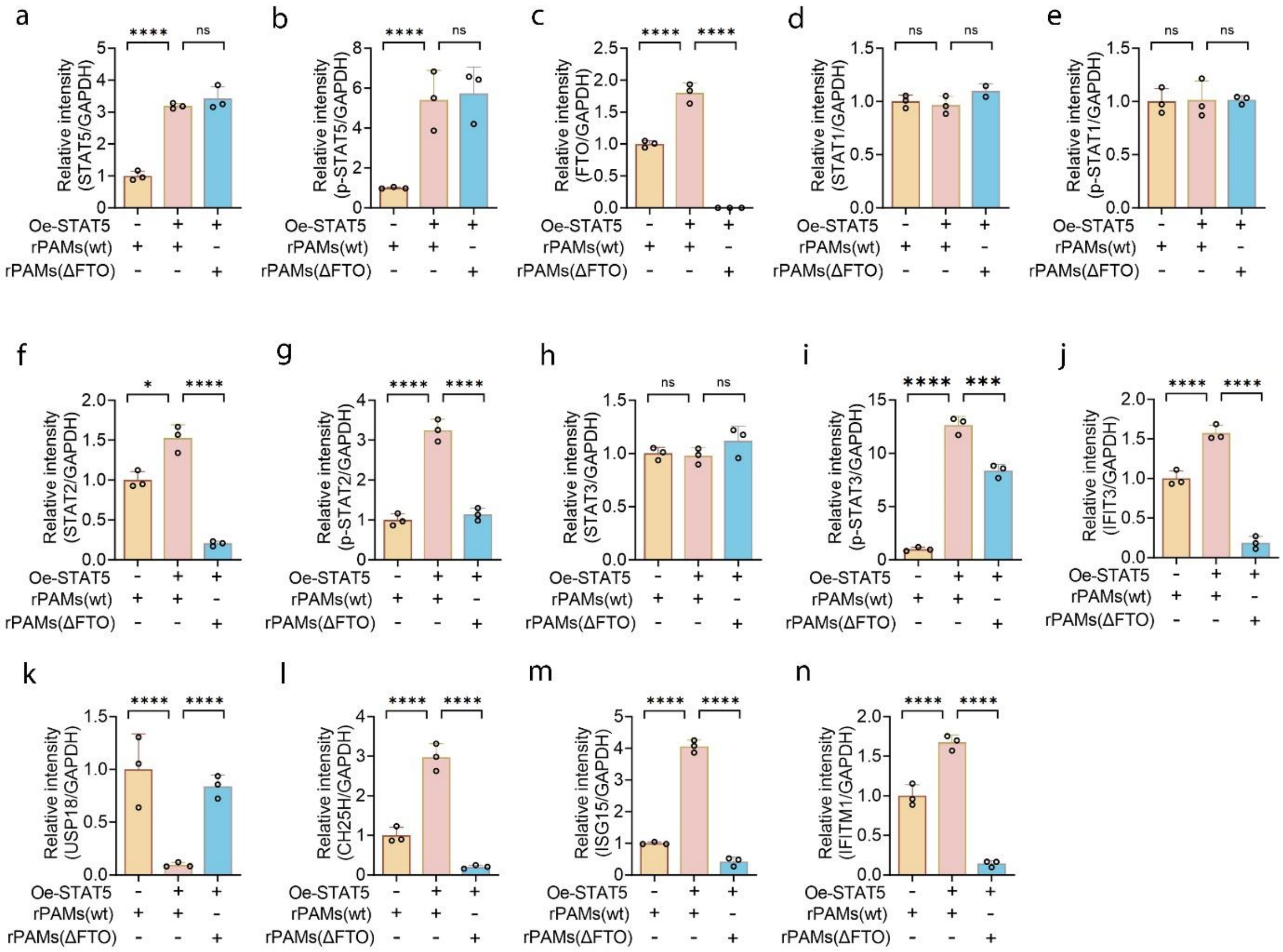

Fig. S43 Densitometric quantification of protein in rPAMs (wild-type and FTO-knockout, ΔFTO) transfected with or without Oe-STAT5. Relative protein levels normalized to GAPDH are shown for (a, b) STAT5 and p-STAT5, (c) FTO, (d, e) STAT1 and p-STAT1, (f, g) STAT2 and p-STAT2, (h, i) STAT3 and p-STAT3, and (j–n) ISGs and IFN negative regulators (IFIT3, USP18, CH25H, ISG15, IFITM1). Data represent mean ± SD (n = 3 independent experiments). **P ≤ 0.05; **P ≤ 0.01; ***P ≤ 0.001; ****P ≤ 0.0001*; ns, not significant.

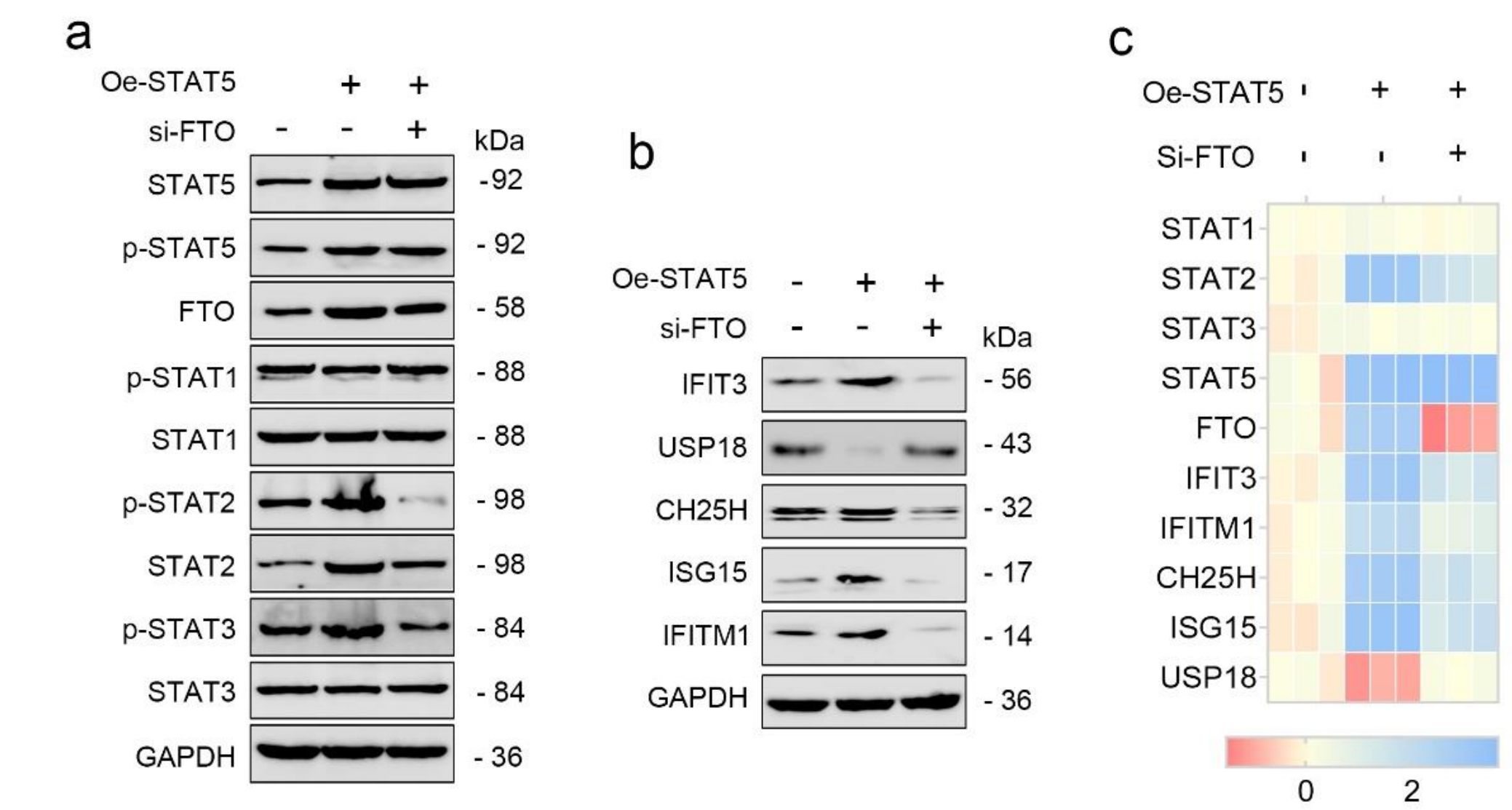

Fig. S44 Marc-145 cells were transfected with Oe-STAT5 in the presence or absence of si-FTO.

(a) Immunoblots of FTO, STAT1–3, STAT5, and their phosphorylated forms. (b) Immunoblots of ISGs (IFIT3, CH25H, ISG15, IFITM1) and the IFN negative regulator USP18. (c) qRT-PCR analysis of the same genes, presented as a Z-score heatmap. GAPDH served as a loading control. Data represent mean ± SD (n = 3 independent experiments).

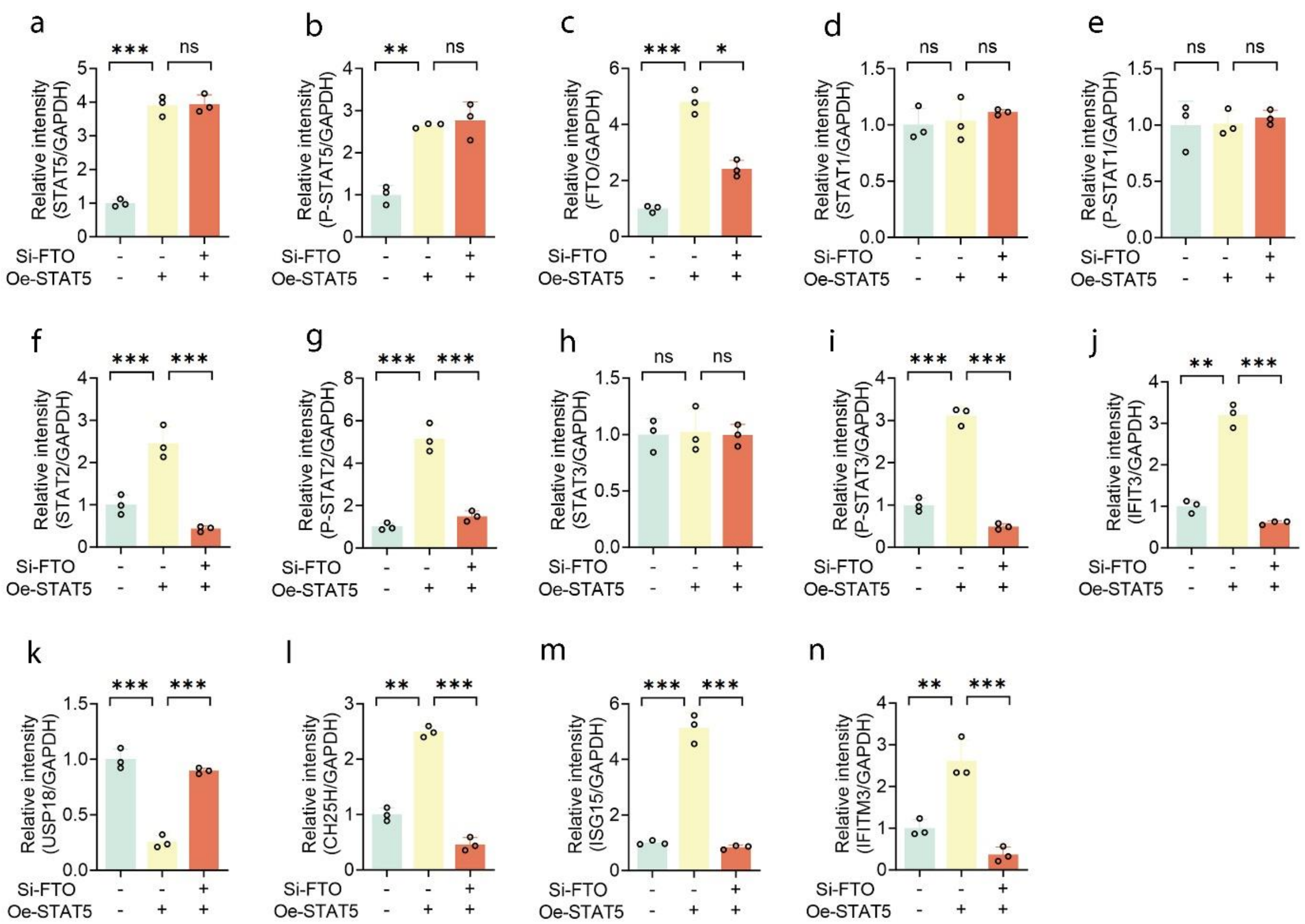

Fig. S45 Densitometric quantification of protein in Marc-145 cells transfected with Oe-STAT5 in the presence or absence of si-FTO. Relative protein levels normalized to GAPDH are shown for (a, b) STAT5 and p-STAT5, (c) FTO, (d, e) STAT1 and p-STAT1, (f, g) STAT2 and p-STAT2, (h, i) STAT3 and p-STAT3, (j–n) ISGs (IFIT3, CH25H, ISG15, IFITM1) and the IFN negative regulator USP18. Data represent mean ± SD (n = 3 independent experiments). *$P \leq 0.05$; **$P \leq 0.01$; ***$P \leq 0.001$*; ns, not significant.

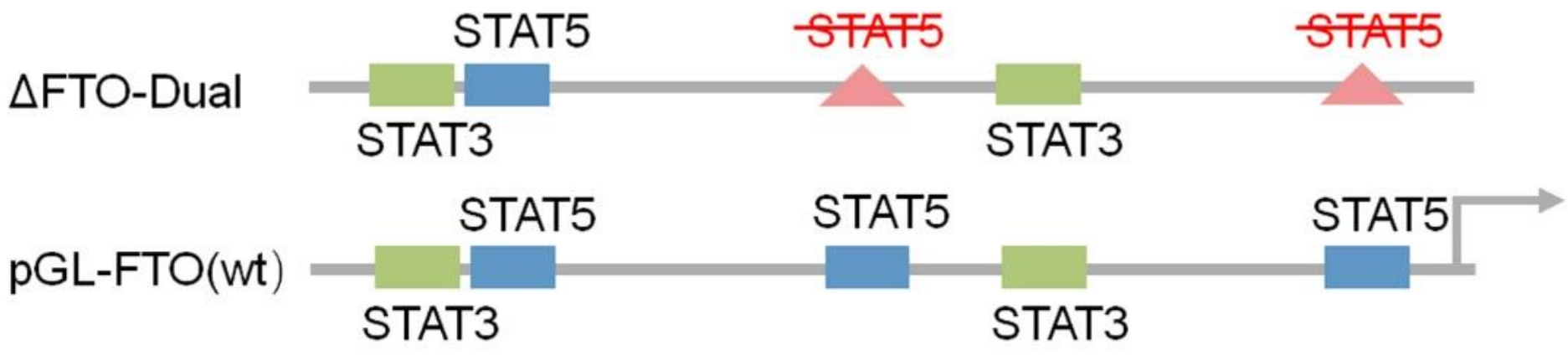

Fig. S46. Schematic representation of the FTO promoter constructs and Bioinformatic analysis of STAT3/5 binding sites within the FTO promoter. In the wild-type reporter (pGL-FTO [wt]), all predicted binding sites are intact. In the mutant construct (ΔFTO-dual), two STAT5 binding sites were simultaneously mutated to generate the dual-site mutant.

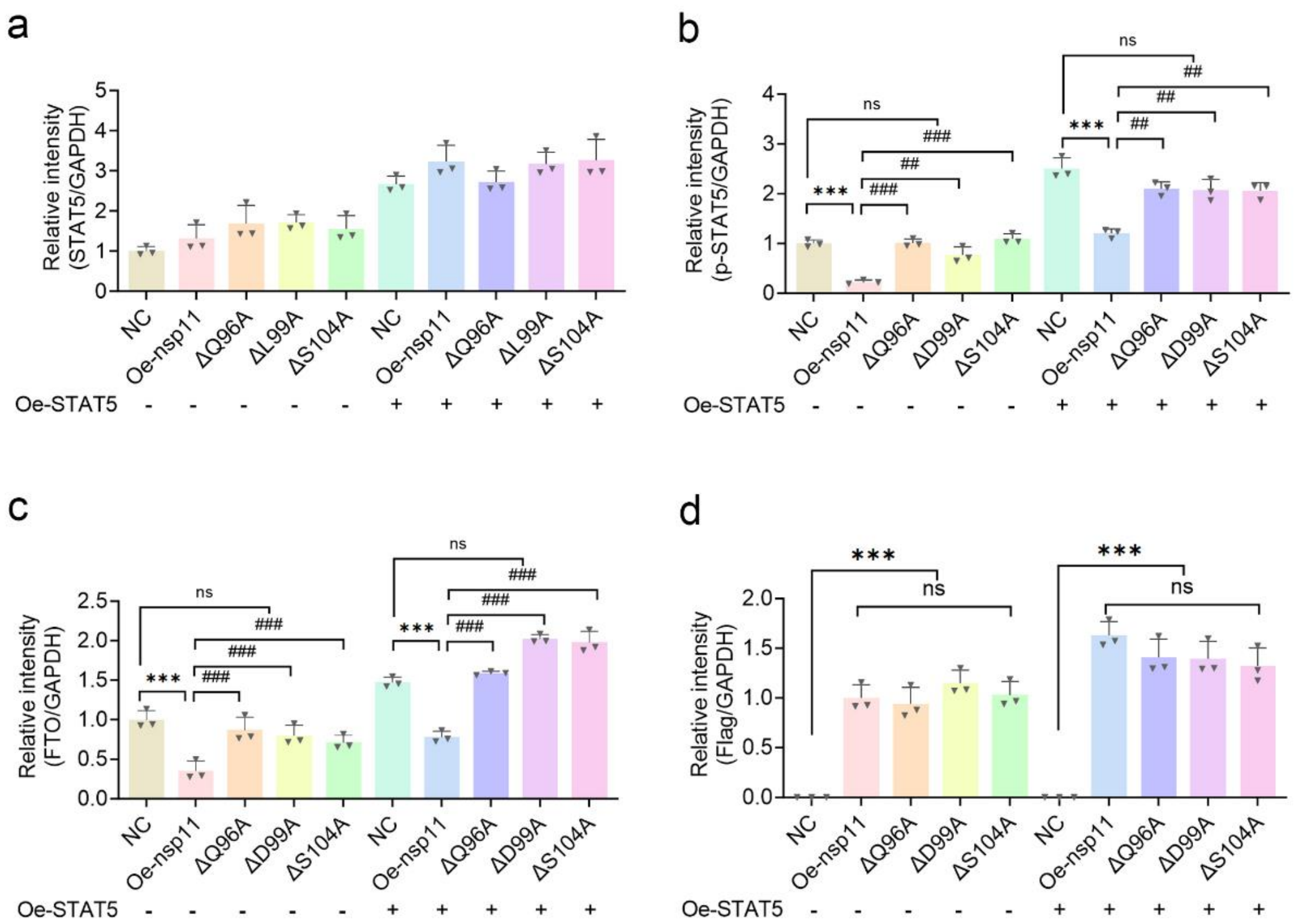

Fig. S47. Densitometric quantification of protein from rPAMs transfected with wild-type or mutant nsp11 (Q96A, D99A, S104A) in the presence or absence of Oe-STAT5. Relative protein levels normalized to GAPDH are shown for (a) STAT5, (b) p-STAT5, (c) FTO, and (d) Flag-nsp11. Data represent mean ± SD from three independent experiments. *$P \leq 0.05$; **$P \leq 0.01$; ***$P \leq 0.001$*; ns, not significant.

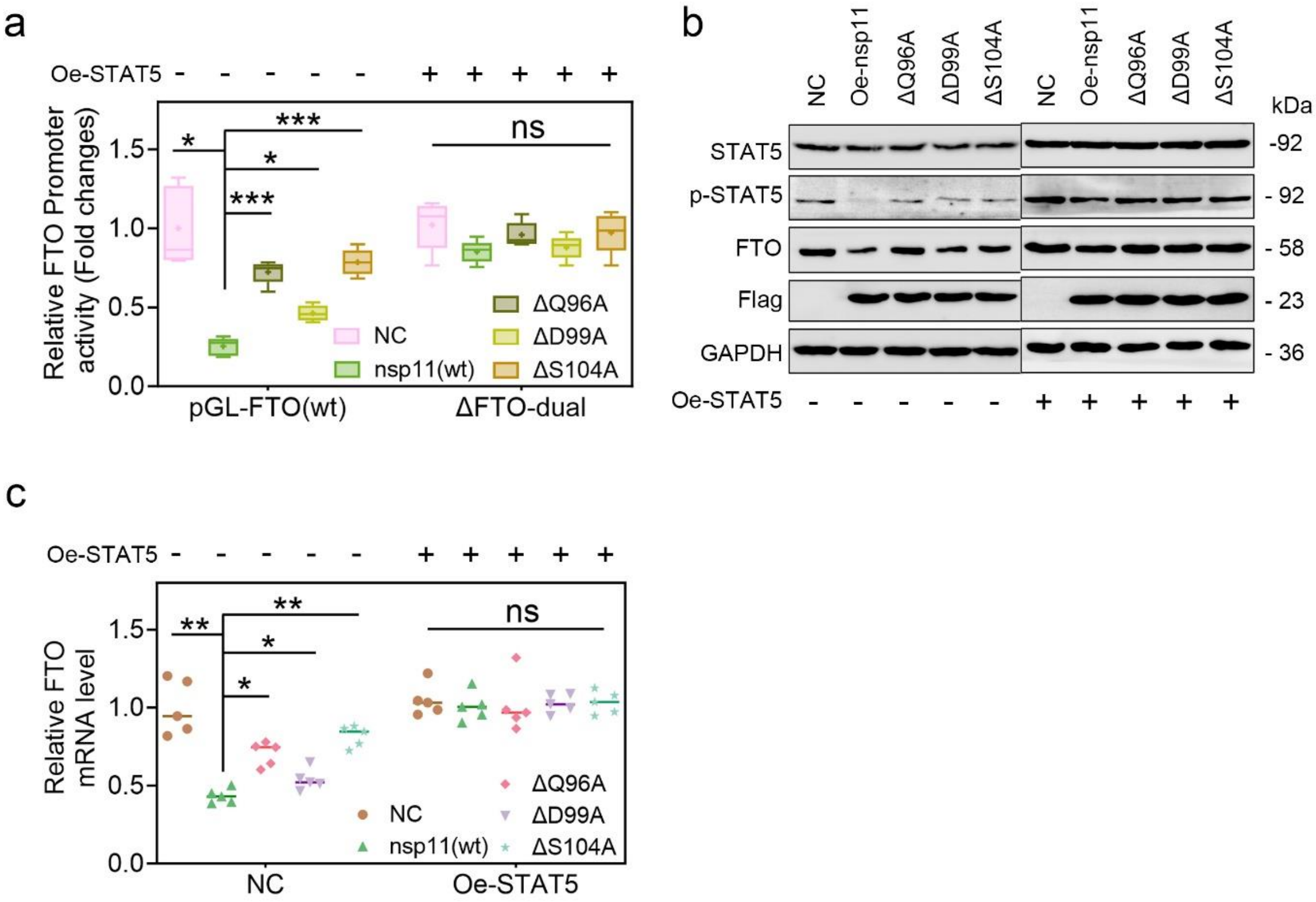

Fig. S48. Marc-145 cells were transfected with wild-type (WT) or mutant nsp11 (Q96A, D99A, S104A), together with luciferase reporter plasmids [pGL-FTO (wt) or ΔFTO-dual] and Oe-STAT5. (a) Dual-luciferase assay of FTO promoter activity. (b) Immunoblots of FTO, STAT5, p-STAT5, and Flag-tagged nsp11, with GAPDH as loading control. (c) qRT-PCR analysis of FTO mRNA expression. Data are mean ± SD from five independent experiments. *$*P \leq 0.05$; $**P \leq 0.01$; $***P \leq 0.001$; $****P \leq 0.0001$*; ns, not significant.

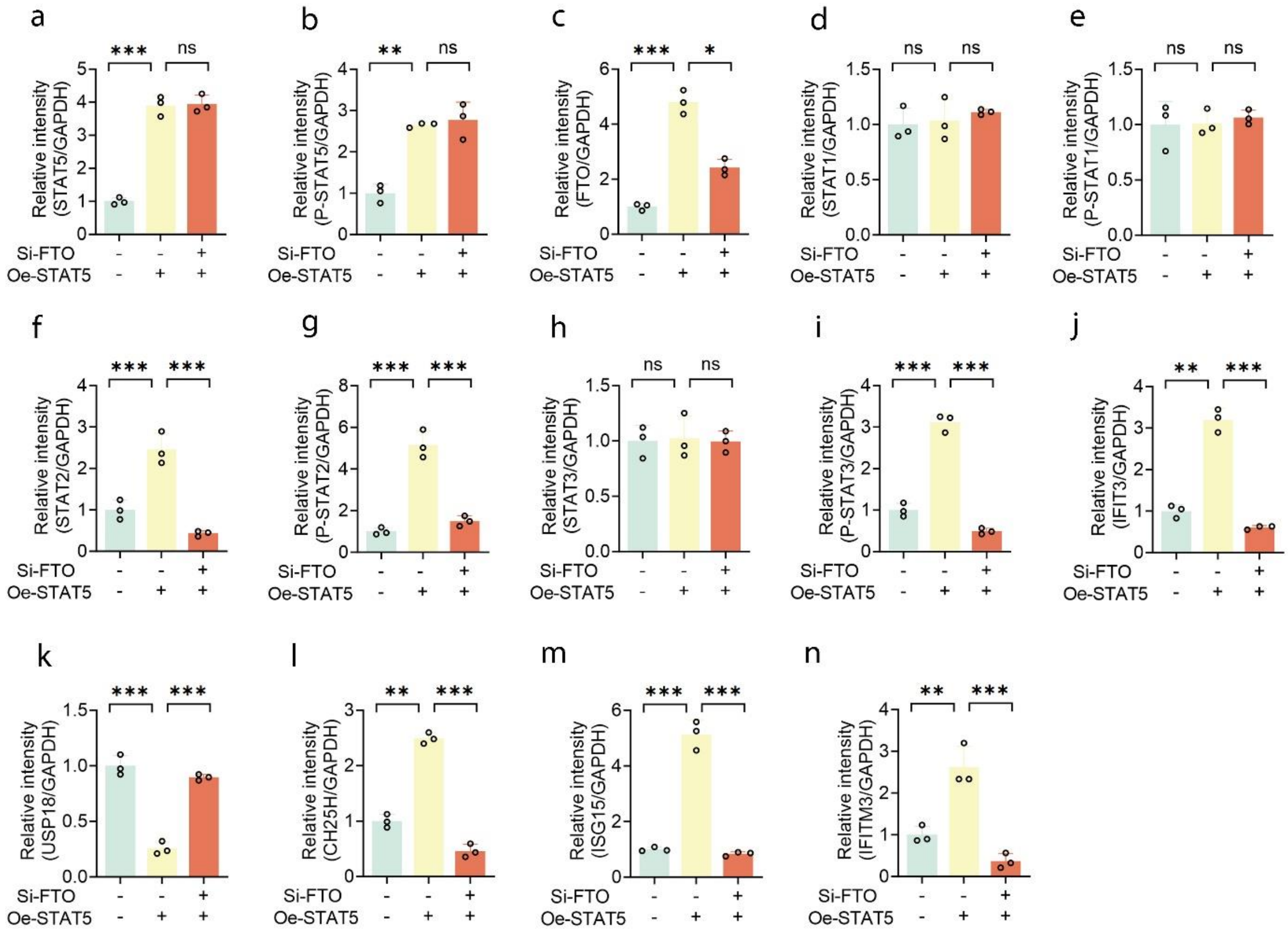

Fig. S49. Densitometric quantification of protein from Marc-145 cells transfected with wild-type or mutant nsp11 (Q96A, D99A, S104A) in the presence or absence of Oe-STAT5. Relative protein levels normalized to GAPDH are shown for (a) STAT5, (b) p-STAT5, (c) FTO, and (d) Flag-nsp11. Data represent mean ± SD from three independent experiments. **P* ≤ *0.05;* ***P* ≤ *0.01;* ****P* ≤ *0.001*; ns, not significant.

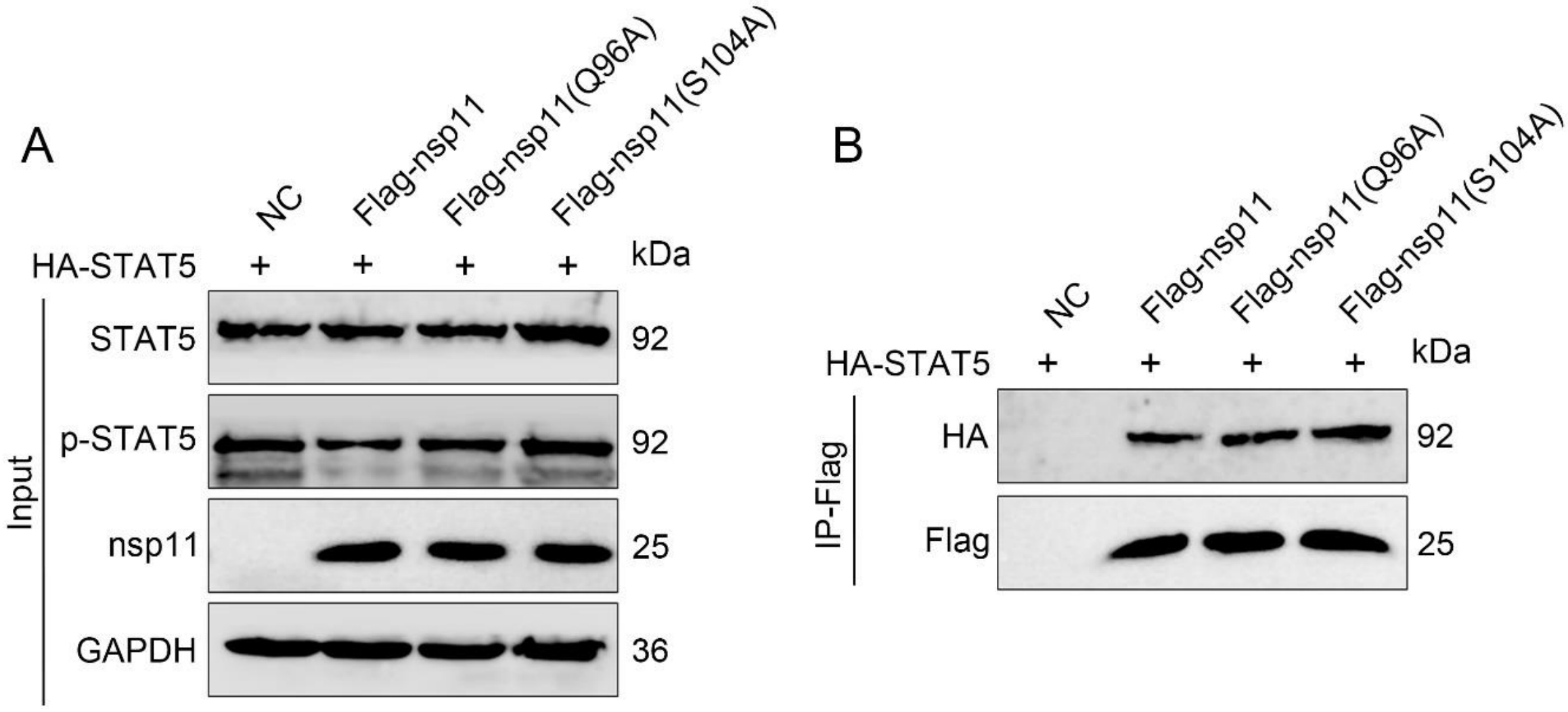

Figure S50. Co-immunoprecipitation analysis of nsp11 and STAT5. (a) Input samples showing the expression of HA-STAT5, p-STAT5, Flag-nsp11, Flag-nsp11(Q96A), and Flag-nsp11(S104A) in the indicated groups. GAPDH was used as the loading control. (b) Co-immunoprecipitation assay performed using an anti-Flag antibody, followed by immunoblotting with anti-HA and anti-Flag antibodies.

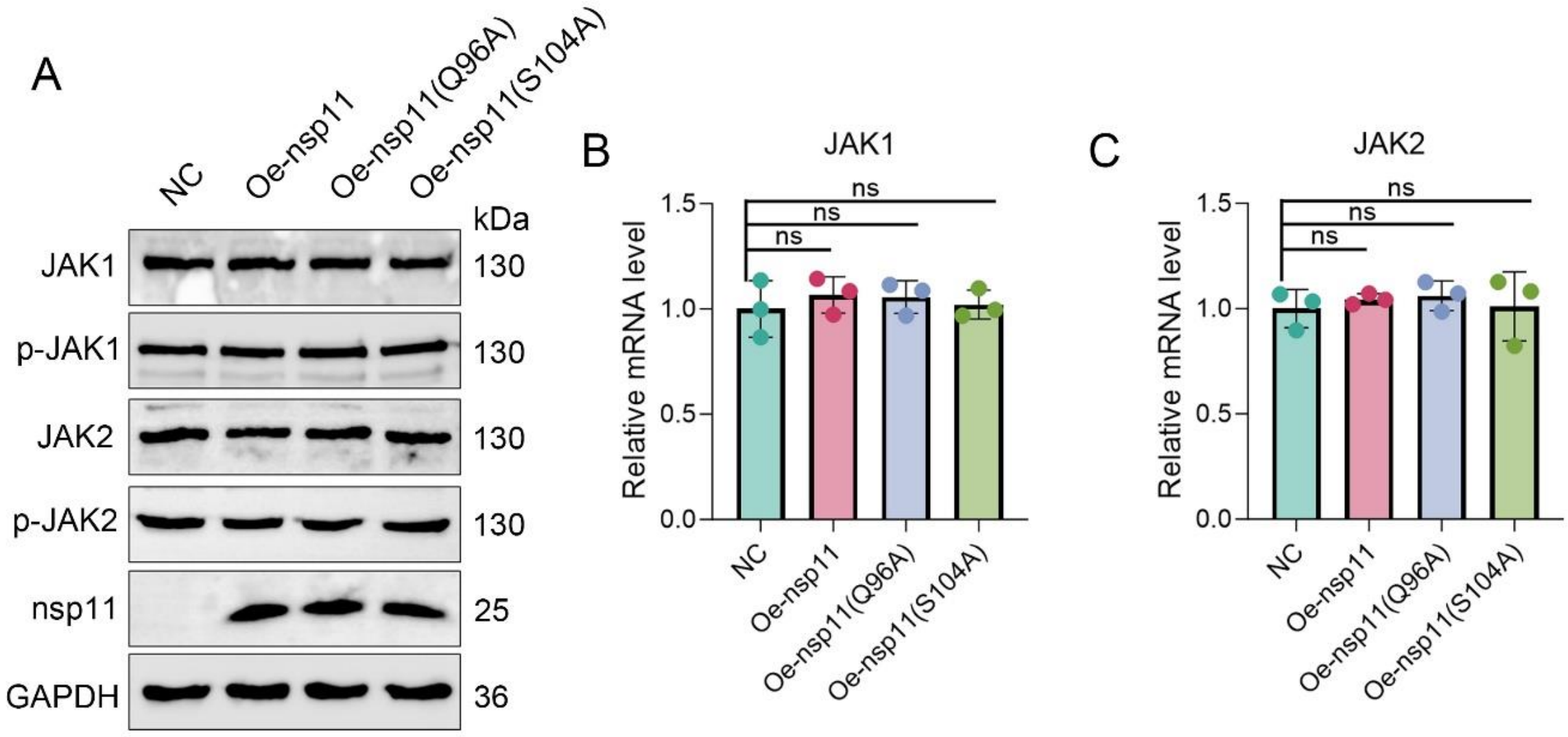

Figure S51. nsp11 does not affect JAK1/2 expression or phosphorylation. (a) Western blot analysis of JAK1, p-JAK1, JAK2, and p-JAK2 protein levels in cells overexpressing wild-type nsp11 or the nsp11 mutants Q96A and S104A. GAPDH was used as the loading control. (b, c) RT-qPCR analysis of the mRNA levels of JAK1 (b) and JAK2 (c) in the indicated groups. GAPDH was used as the internal control. Data are presented as the mean ± SD from three independent experiments. ns, not significant.

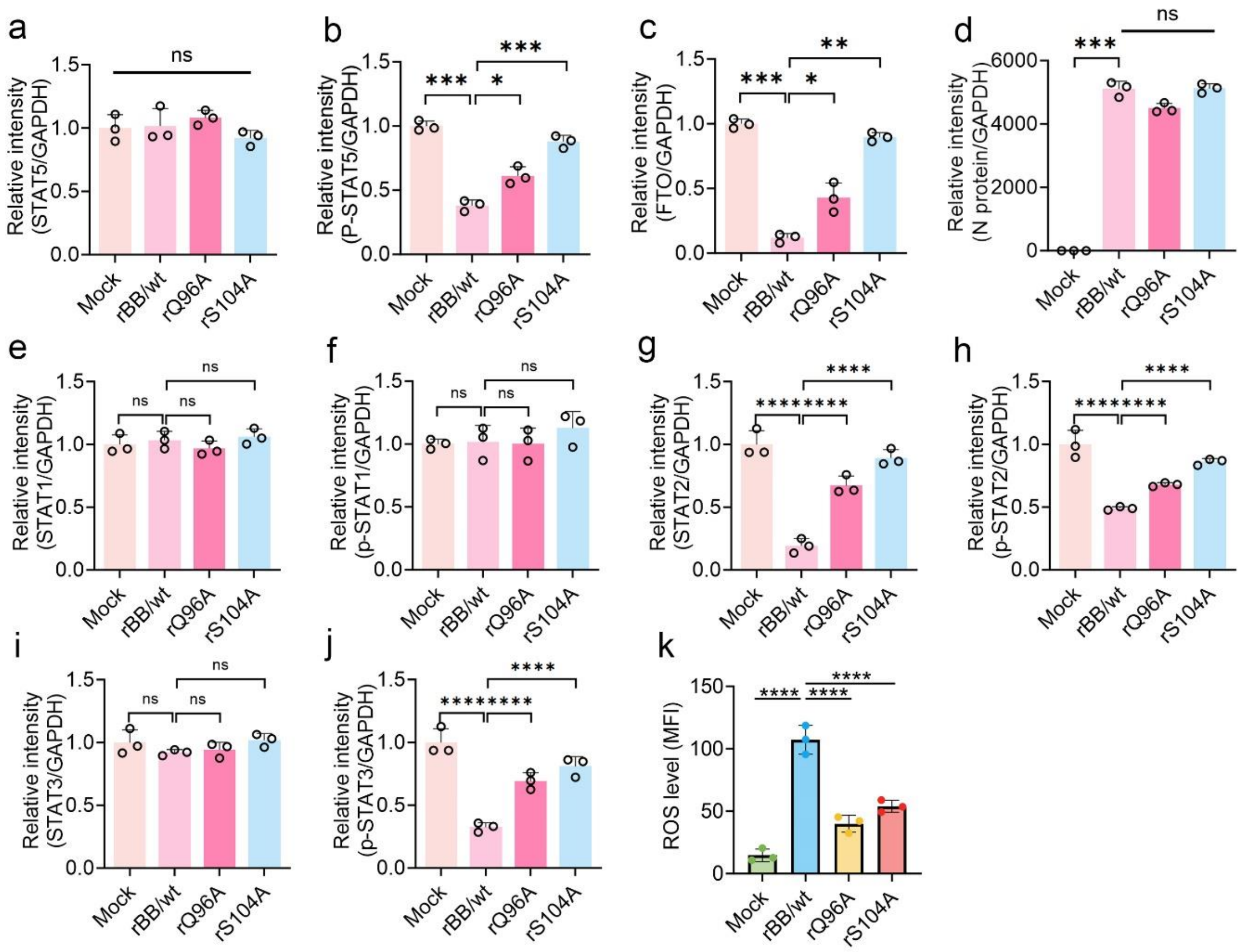

Fig. S52 Densitometric quantification of protein from rPAMs infected with rBB/wt, rQ96A, or rS104A for 36 h. Relative protein levels normalized to GAPDH are shown for (a, b) STAT5 and p-STAT5, (c) FTO, (d) viral N protein, (e, f) STAT1 and p-STAT1, (g, h) STAT2 and p-STAT2, and (i, j) STAT3 and p-STAT3. (k) Quantification of the mean fluorescence intensity (MFI) from the corresponding immunofluorescence images in the indicated groups. Data represent mean ± SD (n = 3 biologically independent samples). *$*P \leq 0.05$; $**P \leq 0.01$; $***P \leq 0.001$; $****P \leq 0.0001$*; ns, not significant.

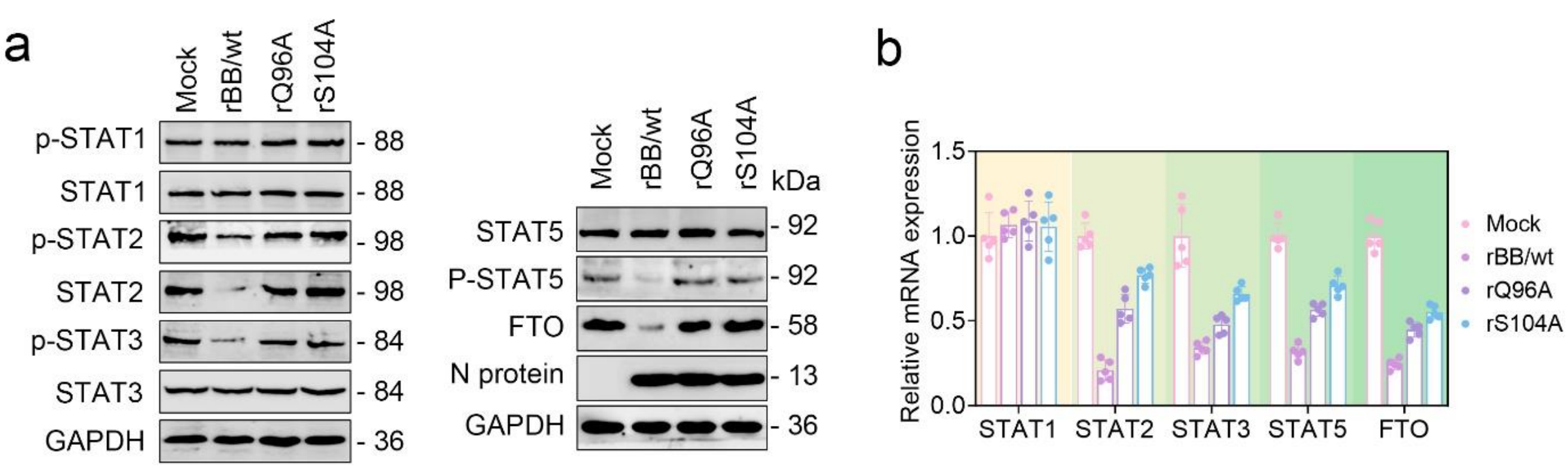

Fig. S53 Marc-145 cells were infected with rBB/wt, rQ96A, or rS104A for 36 h. (a) Immunoblots of FTO, STAT1–3, STAT5, their phosphorylated forms, and viral N protein, with GAPDH as loading control. (b) qRT-PCR analysis of FTO and STAT transcripts. Data are shown as mean ± SD (n = 3 biologically independent samples).

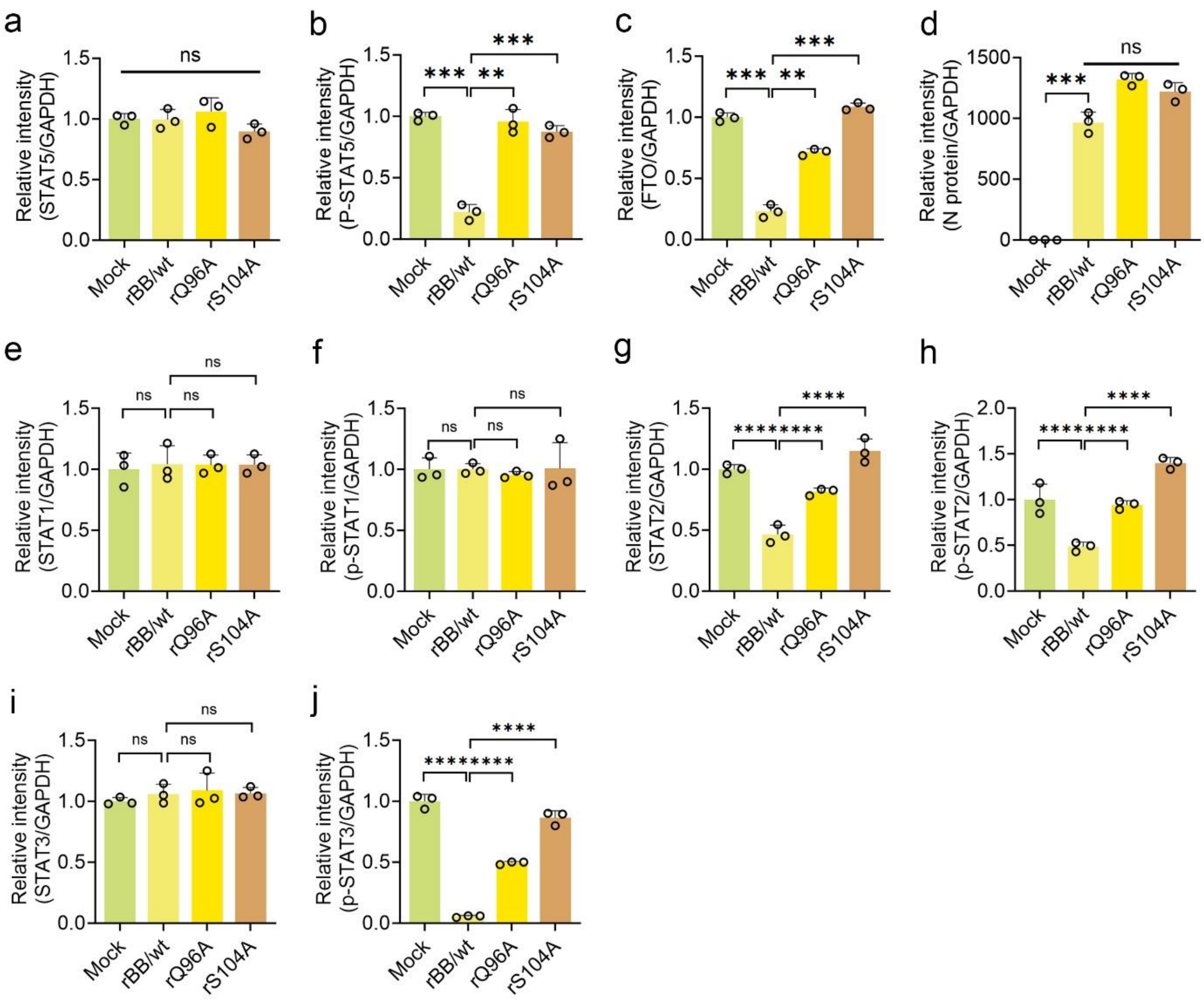

Fig. S54 Densitometric quantification of protein from Marc-145 cells infected with rBB/wt, rQ96A, or rS104A for 36 h. Relative protein levels normalized to GAPDH are shown for (a, b) STAT5 and p-STAT5, (c) FTO, (d) viral N protein, (e, f) STAT1 and p-STAT1, (g, h) STAT2 and p-STAT2, and (i, j) STAT3 and p-STAT3. Data represent mean ± SD (n = 3 biologically independent samples). **P ≤ 0.05; **P ≤ 0.01; ***P ≤ 0.001; ****P ≤ 0.0001*; ns, not significant.

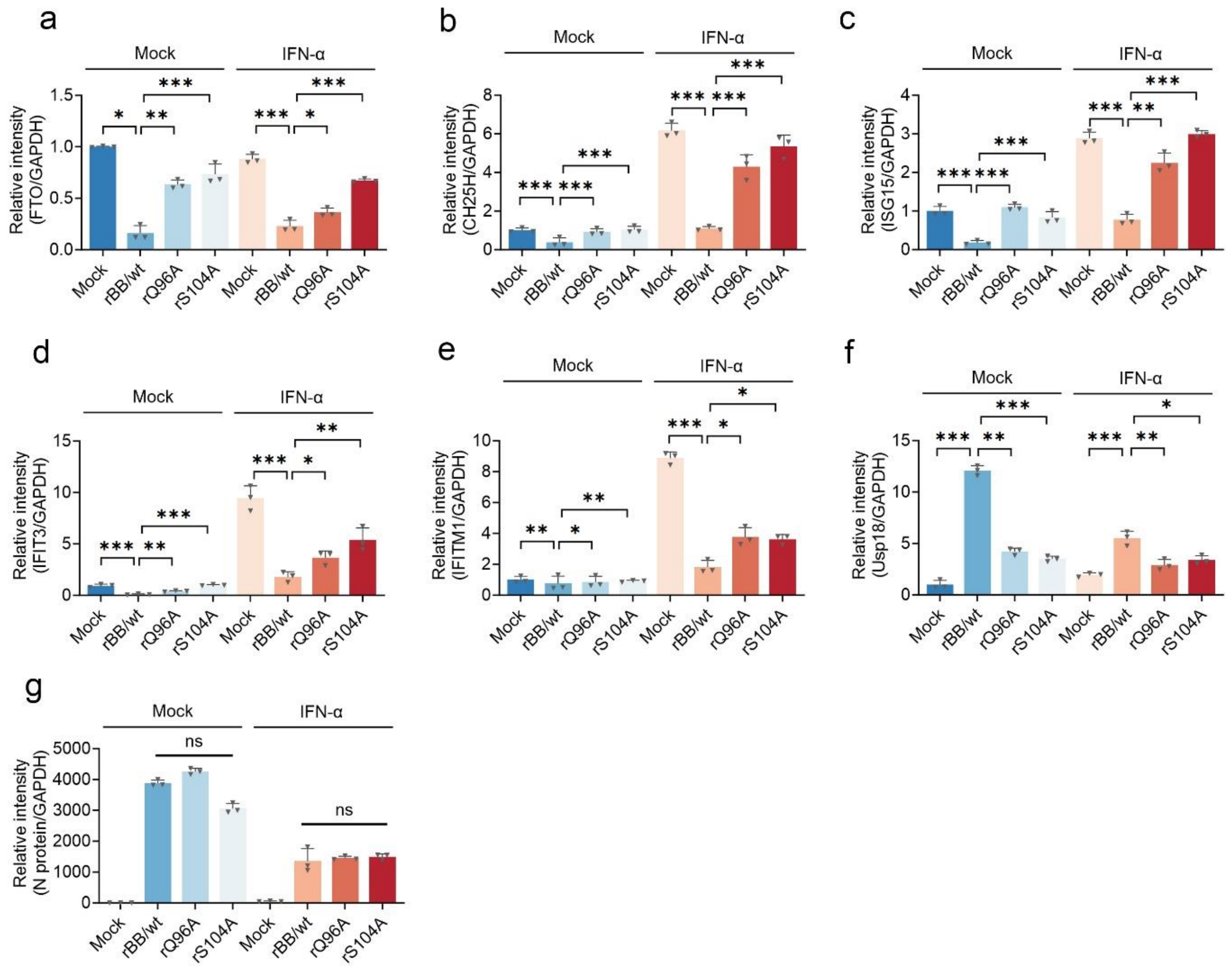

Fig. S55 Densitometric quantification of protein in PAMs infected with recombinant PRRSV (rBB/wt, rQ96A, rS104A) for 36 h, with or without IFN-α treatment. Relative protein levels normalized to GAPDH are shown for (a) FTO, (b–e) ISGs (CH25H, ISG15, IFIT3, IFITM1), (f) IFN negative regulator USP18, and (g) viral N protein. Data represent mean ± SD (n = 5 biologically independent samples). **P ≤ 0.05; **P ≤ 0.01; ***P ≤ 0.001; ****P ≤ 0.0001*; ns, not significant.

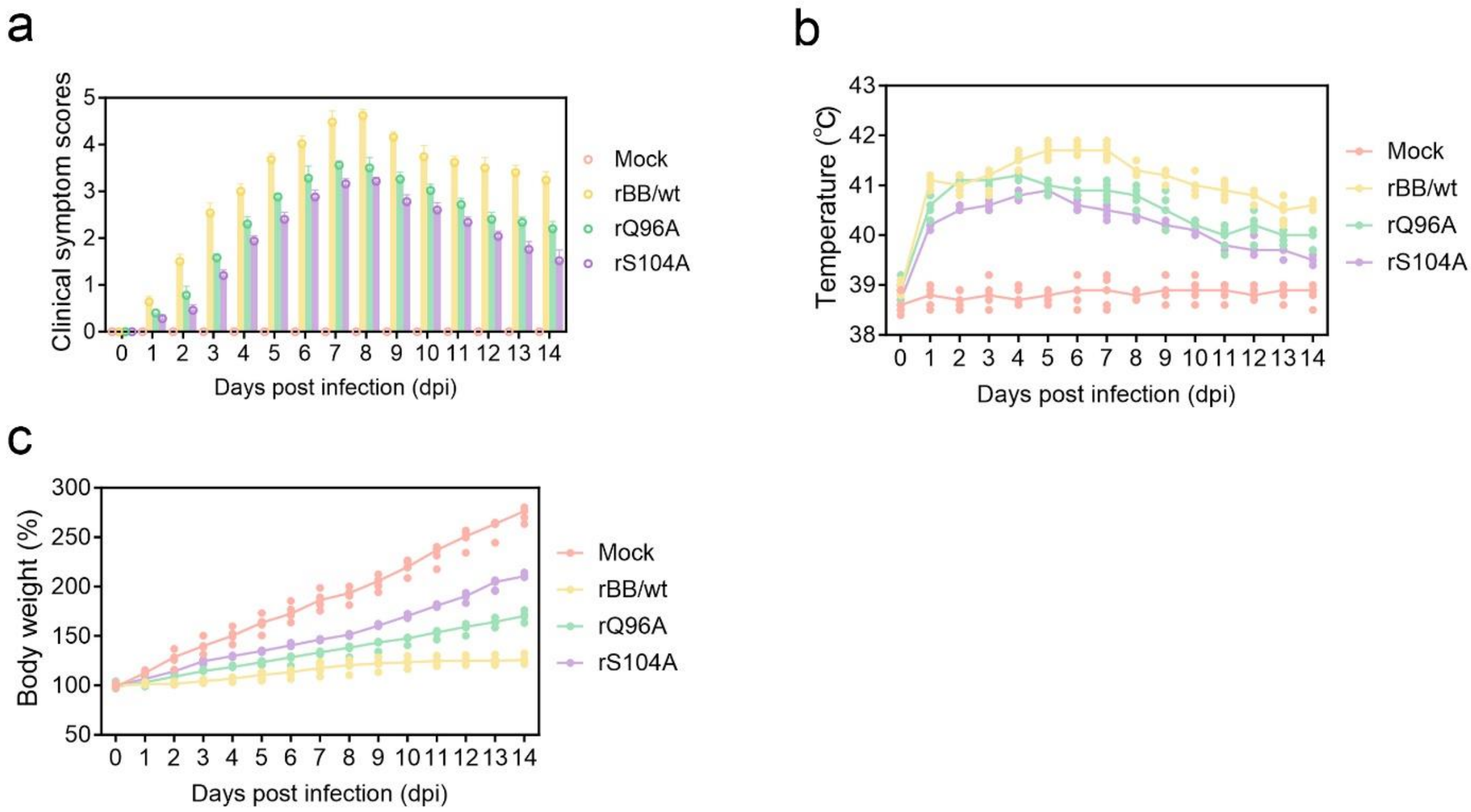

Fig. S56 Clinical evaluation of recombinant PRRSV infection in piglets. (a) Daily clinical symptom scores of 6-week-old SPF piglets infected with rBB/wt, rQ96A, or rS104A compared with mock controls (0–14 dpi). (b) Rectal temperature profiles during the infection period. (c) Relative body weight changes expressed as percentages of baseline. Data are shown as mean ± SD (n = 5 per group).

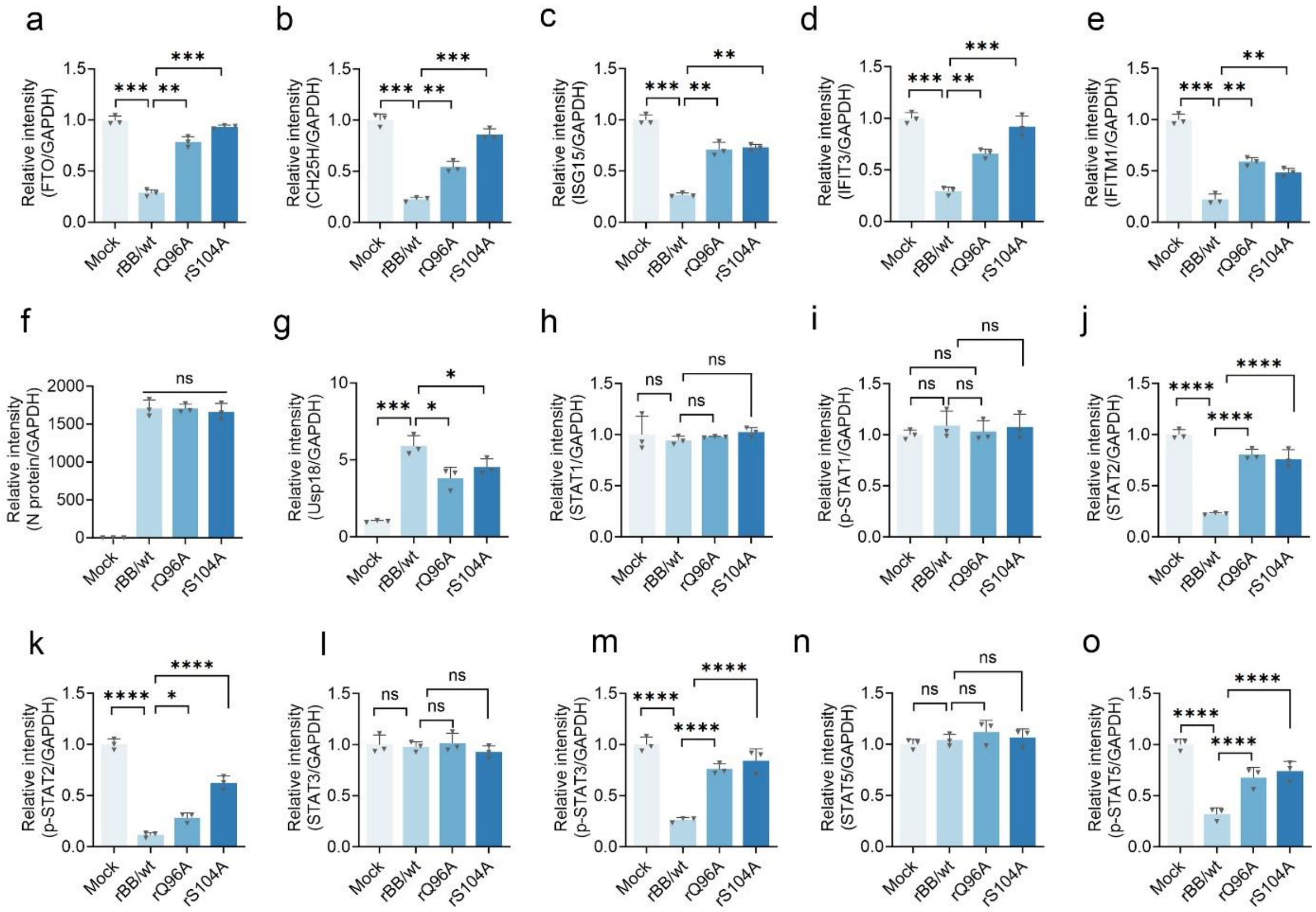

Fig. S57 Densitometric quantification of protein from lung tissues of piglets infected with recombinant PRRSV. Relative protein levels normalized to GAPDH are shown for (a) FTO, (b–e) ISGs (CH25H, ISG15, IFIT3, IFITM1), (f) viral N protein, (g) IFN negative regulator USP18, (h, i) STAT1 and p-STAT1, (j, k) STAT2 and p-STAT2, (l, m) STAT3 and p-STAT3, and (n, o) STAT5 and p-STAT5. Data represent mean ± SD (n = 5 biologically independent samples). *$P \leq 0.05$; **$P \leq 0.01$; ***$P \leq 0.001$; ****$P \leq 0.0001$; ns, not significant.
