# Supplementary material for: PRRSV suppresses FTO-dependent m6A demethylation to reprogram STAT signaling and innate immunity
Source: mBio. 2026 Aug 13;17(9):e00843-26. doi: 10.1128/mbio.00843-26 (PMC13556206; doi:10.1128/mbio.00843-26)
Supplement: Supplementary file 3 [file mbio.00843-26-s0003.pdf]

Table S1. Primer sequences used for the construction of plasmids expressing porcine FTO, STAT1, STAT2, STAT3, STAT5, and PRRSV nsp11 truncations/mutants.

Primer	Sequence (5'-3')[a]
nsp11-F	CCCAAGCTTATGGGGTCGAGCTCCCCGCTCCC
nsp11-R	CCGCTCGAGTCACTTATCGTCGTCATCCTTGTAATCTTCAAGTTGAAAATAGGCCGT
nsp11- Ecor I-91-F	CCCGAATTCATGGTTAGAGGCGAGGCTCAAGTGCTTC
nsp11- Ecor I-107-F	CCCGAATTCATGCGAATTGAGGTAGATTGTCGAGAGT
nsp11- Xho I -90-R	TGCCTCGAGTCACTTATCGTCGTCATCCTTGTAATCAAATTTTGTGAGATAGTATGACA
nsp11- Xho I -106-R	TGCCTCGAGTCACTTATCGTCGTCATCCTTGTAATCGCCGGTACTGAAGACTGTCTCCG
nsp11- EcoR I -91-F	CCCAAGCTTGCCACCATGGTTAGAGGCGAGGCTCAAGTGCTTC
nsp11- EcoR I -107-F	CCCAAGCTTGCCACCATGCGAATTGAGGTAGATTGTCGAGAGT
STAT2-Kpn I-F	CGGGGTACCATGGCGCAGTGGGAAATGCTGCAGA
STAT2-Xho I-R(HA)	CCGCTCGAGTCAAGCGTAATCTGGAACATCGTATGGGTACACAGTTTTTTCCATTGGCTCA
STAT3-Kpn I-F	CGGGGTACCATGGCCCAATGGAATCAGCTACAGC
STAT3-Xho I-R(HA)	CCGCTCGAGTCAAGCGTAATCTGGAACATCGTATGGGTACATGGGGGAGGTAGCGCACTCC
STAT5-Kpn I-F	CGGGGTACCTCAGGGGACA GTGACTGCTG
STAT5-Xho I-R(HA)	CCGCTCGAGTCAAGCGTAATCTGGAACATCGTATGGGTAAATCTGAAGC CATTCACCACGCGGG
nsp11-91-F	TATCTCACAAAATTTGGTAGAGGCGAG
nsp11-91-R	TTGAGCCTCGCCTCTACCAAATTTTGT
nsp11-92-F	CTCACAAAATTTGTTGGAGGCGAGGCT
nsp11-92-R	CACTTGAGCCTCGCCTCCAACAAATTT
nsp11-94-F	AAATTTGTTAGAGGCGGGGCTCAAGTG
nsp11-94-R	GAAGCACTTGAGCCCCGCCTCTAAC
nsp11-95-F	TTTGTTAGAGGCGAGGGTCAAGTGCTT
nsp11-95-R	CTCCGGAAGCACTTGACCCTCGCCTCT
nsp11-96-F	GTTAGAGGCGAGGCTGGAGTGCTTCCG
nsp11-96-R	ACTGTCTCCGGAAGCACTCCAGCCTCGCCT
nsp11-97-F	TTAGAGGCGAGGCTCAAGGGCTTCCGGAGA
nsp11-97-R	AAGACTGTCTCCGGAAGCCCTTGAGCCTC
nsp11-98-F	TTAGAGGCGAGGCTCAAGTGGGTCCGGAGACA
nsp11-98-R	GAAGACTGTCTCCGGACCCACTTGAGC
nsp11-99-F	GAGGCTCAAGTGCTTGGGGAGACAGTC
nsp11-99-R	ACTGAAGACTGTCTCCCCAAGCACTTG
nsp11-100-F	GCTCAAGTGCTTCCGGGGACAGTCTTC
nsp11-100-R	GGTACTGAAGACTGTCCCCGGAAGCAC
nsp11-102-F	GTGCTTCCGGAGACAGGCTTCAGTACC
nsp11-102-R	ATTCGGCCGGTACTGAAGCCTGTCTCCGGA
nsp11-103-F	TTCCGGAGACAGTCGGCAGTACC
nsp11-103-R	AATTCGGCCGGTACTGCCGACTGTCTC
nsp11-104-F	GGAGACAGTCTTCGGTACCGGCCGAAT
nsp11-104-R	CTCAATTCGGCCGGTACCGAAGACTGT
nsp11-105-F	GAGACAGTCTTCAGTGGCGGCCGAATT
nsp11-105-R	TACCTCAATTCGGCCGCCACTGAAGAC

a The genomic positions of the primers were based on the sequence under GenBank accession number XM_001928655. The restriction enzyme sites used for cloning are underlined. The C-terminal flag tag sequence in the "R" primers is indicated by double underlining. Locations of mutations are indicated in bold.

Table S2. Primer sequences used for the amplification and mutagenesis of the porcine FTO promoter region for luciferase reporter assays.

Primer	Sequence (5'-3')[a]
FTO-promoter-F	CCCAAGCTTCCAAGTCTCAGGTGGAGGAG
FTO-promoter -R	CCGCTCGAGCAGAACTTCCACGTGCCATGGG
ΔFTO(-185~-176)-F	AGCACACGGGGCCCGTGAATACTCA
ΔFTO(-185~-176)-R	TGAGTATTCACGGGCCCCGTGTGCT
ΔFTO(-438~-429)-F	CAATATGGCAGCCACTAGGTAGATG
ΔFTO(-438~-429)-R	CATCTACCTAGTGGCTGCCATATTG
ΔFTO(-604~-595)-F	CCGGGGTTGCCGTGAGCTGTGGTGT
ΔFTO(-604~-595)-R	ACACCACAGCTCACGGCAACCCCGG
ΔFTO(-982~-973)-F	GAGCTGCTCCCCCCACAGGTGAAAT
ΔFTO(-982~-973)-R	ATTTCACCTGTGGGGGGAGCAGCTC
ΔFTO(-1086~-1078)-F	GACAGGTGGAATTGAGGCGGGAAGA
ΔFTO(-1086~-1078)-R	CTGTCCACCTTAACTCCGCCCTTCT

Table S3. Primer sequences used for RT-qPCR analysis of host genes (FTO, STATs, ISGs, cytokines) and viral genes (PRRSV N, nsp11).

Name	Sequence (5'-3')
STAT1-(p) F	CTCATTTGTGGTGGAA
STAT1-(p) R	TCATCCAGCTGCGACAGAAG
STAT2-(p) F	TGGATGGAGAGTTGGAGCAG
STAT2-(p) R	CTTGCTCCCAGTCTTGAGGA
STAT3-(p) F	TGCTGGAGGAGAGAATCGT
STAT3-(p) R	GGGAATTTGACCAGCAATC
STAT5-(p) F	GCTGGACATCCTGCAGTCCT
STAT5-(p) R	GTGATGGTGCTGTTGAGGTC
GAPDH-(p) F	CCTTCCGTGTCCCTACTGCCAAC
GAPDH-(p) R	GACGCCTGCTTCACCACCTTCT
STAT1-(m) F	CACGCTGCCAATGATGTTTC
STAT1-(m) R	ATCTGTATTGGGTCTTCTTG
STAT2-(m) F	CCTGTAATGGAGCCCACACT
STAT2-(m) R	GTAAGCCTCATCCACGGTGT
STAT3-(m) F	GTGATGCTTCCCTGATTGTG
STAT3-(m) R	GCAAGGAGTGGGTCTCTAGG
STAT5-(m) F	CTGAGCACCTCTGCCAGCAG
STAT5-(m) R	CACGTTCAGCTTCCCGCCCA
GAPDH-(m) F	TCATGACCACAGTCCATGCC
GAPDH-(m) R	GGATGACCTTGCCCACAGCC
Mx1-(p) F	AGCGCAGTGACACCAGCGAC
Mx1-(p) R	GCCCGGTTCAGCCTGGGAAC
GBP1-(p) F	GAAGGGTGACAACCAGAACGAC
GBP1-(p) R	AGGTTCCGACTTTGCCCTGATT
IFIT3-(p) F	CCGCCATCATGAGTGAGGTC
IFIT3-(p) R	TCATGCCAGACATGTTCTTCCT
USP18-(p) F	CAATGACTCCAATGICTGTT
USP18-(p) R	TCTGAAGGTGTAGATGCATC
SOCS3-(p) F	GCCCCCCTAGAAGAGCCTATTA
SOCS3-(p) R	TCCGACAGAGATGCTGGAGA
CH25H -(p) F	CACTCACAGACTAGTACCTTTCG
CH25H -(p) R	TCCCAGTATTTTGTCCCAGTG
IFITM1-(p) F	CCGGAAGATGGGGGAGACAT
IFITM1-(p) R	CCCAGGATCAGAGCCCAGATG
ISG15-(p) F	GGTGCAAAGCTTCAGAGACC
ISG15-(p) R	GTCAGCCAGACCTCATAGGC

Viperin-(p)-F	GGGCACAGGAGGACAGCCAT
Viperin-(p)-R	CGGCTCTCCGCCTGAAAAGT
OAS-(p)-F	GAGGTGGAGTTTGATGTCCT
OAS-(p)-R	CAGCTCCGTGAAGCAGGTGG
TRIM25-(p)-F	CAACTTCTGCGGGTTGTGCC
TRIM25-(p)-R	TCGGCCTGCAGGAACTGCTC
IFN-α-(p) F	TCCTGCCTGGACCACAGAAG
IFN-α-(p) R	TTCCAGGTCCCTGAGCTGCT
IFN-β-(p) F	ACCACCACAATTCCAGAAGG
IFN-β-(p) R	TCCATCTGCCCATCAAGTTC
Mx1-(m) F	ACATCCAGAGGCAGGAGACAATC
Mx1-(m) R	TCCACCAGATCAGGCTTCGTCAA
GBP1-(m) F	CCTCGCTCTTAAACTTCAGGAACA
GBP1-(m) R	CCTTTCGTCGTCTCATTTTCGT
IFIT3-(m) F	AAACCTCACACCCAACGAAAA
IFIT3-(m) R	CCACCCTTAGCCACTTCCT
USP18-(m) F	CAGGAGTCCCTGATTTGCGT
USP18-(m) R	GGGCTGGACGAAACATCTCA
SOCS3-(m) F	GAGAAGATTCCGCTGGTACTG
SOCS3-(m) R	GCAGCTGGGTCACTTTCTCATA
CH25H -(m) F	ATCACCACATACGTGGGCTTT
CH25H -(m) R	GTCAGGGTGGATCTTGTAGCG
IFITM1-(m) F	TCAACATCCACAGCGAGACC
IFITM1-(m) R	TGTCACAGAGCCGAATACCAG
ISG15-(m) F	GGGACCTGACGGTGAAGATG
ISG15-(m) R	CGCCGATCTTCTGGGTGAT
Viperin-(m)-F	CAAAGAGAGGATTGCTTTTG
Viperin-(m)-R	ACAGCGAGAATGTCCAAATA
OAS-(m)-F	CCACCGCTCTCATGGACGCT
OAS-(m)-R	CTTTTTCTCCCCTCACATTG
TRIM25-(m)-F	TGTTCCCAGCACAACCGGCT
TRIM25-(m)-R	CATCCTGCTGCCTGTTCCTC
FTO-(p) F	AGATTGAGACCATCCAGG
FTO-(p) R	TTCCCCATGCCAAAGTAG
IL-1β-(p) F	CCCAAAACCATCAAAGATGA
IL-1β-(p) R	GACTTCGCTCCCTCTCTTAC
IL-6-(p) F	CTGCCTTCCCTACCCCGGGA
IL-6-(p) R	TTCTCATACTTCTCACACAT
IL-8-(p) F	GGCAGTTTTCCTGCTTTCT
IL-8-(p) R	CAGTGGGGTCCACTCTCAAT
IL-10-(p) F	CTATTGCCTGATCTTCCTGG
IL-10-(p) R	GGTAACCCTTAAAGTCCTCC
IL-13-(p) F	AGGAGCTCATTGAAGAGCTG
IL-13-(p) R	TGGCGCTGCAGTCGGAGATG

TNF-α-(p) F	GTCGCAGGAGCCACCACGCT
TNF-α-(p) R	GTGGGCGACGGGCTTATCTG
FTO-(m) F	TAATGAGGTCGAGTTTGA
FTO-(m) R	TCATTCCTTTGTTCCACG
IL-1β-(m) F	TGGCCCTAAACAGATGAAGT
IL-1β-(m) R	GGGAACCAGCATCTTCCTTA
IL-6-(m) F	CTGCCTTCCCCGCCCCAGTA
IL-6-(m) R	ATGTTACTCCTGTTACATGT
IL-8-(m) F	CTGGCGGTGGCTCTCTTG
IL-8-(m) R	CCTTGGCAAAACTGCACCTT
IL-10-(m) F	TCCGAGATGCCTTCAGCAGA
IL-10-(m) R	TGGGGCATCACCTCCTCCAG
IL-13-(m) F	AGGCCCCGCTCTGCAATGGC
IL-13-(m) R	CCAGCTGAGACCTTGTGCGG
TNF-α-(m) F	AGTCAGATCATCTTCTCGAA
TNF-α-(m) R	TTCTGATGGCACCACCAGCT
PRRSV N F	CCTCTAGCGACTGAAGATGACGTCAGGCATCACT
PRRSV N R	ACTCCACAGTGTAACTTATCCTCCCTGAATCT

Table S4. Primer sequences used for the amplification of the full-length PRRSV (BB0907 strain) genome and for the construction of nsp11 mutant infectious cDNA clones.

**Primer**	**Sequence (5'-3')** [a, b]
A-BB0907-Fwd	GCGTTAATTAAACCGTCATGACGTATAGGTGTTG
A-BB0907-Rev	TGTCTCGAGAATCATCTTTGGGAGAAACC
B-BB0907-Fwd	TTCTTAATTAAATGATTCTCGAGACACCGCC
B-BB0907-Rev	GTGCTTAAGTTCATTACCACCTGTAACGGAT
C1-BB0907-Fwd	GCGTTAATTAAAATGAACTTAAGCACCTATGCC
C1-BB0907-Rev	TTGACACAGAGGTAATCGGGTCGCCAGAC
C2-BB0907-Fwd	GTCTGGCGACCCGATTACCTCTGTGTCAA
C2-BB0907-Rev	CGGGGGAAAATGAAACCTCATGCTGGT
D1-BB0907-Fwd	TCGTTAATTAAGTTTCGGGCGCGCCAGAAAGGG
D-BB0907-Rev (SwaI)	TTCGGCTTGGGATTTAAATATGCATTTTTTTTTTTTTTTTTTTTT
D-BB0907-Rev (SpeI)	CTCACTAGTAACGGCCGCCAGTGTGCTGGAATTCGGCTTGGGATTT
XhoI-BB0907-Fwd	GGTACCATGGCCAAACTCGAGGCTTTTGCCGATACC
XhoI-BB0907-Rev	GGTATCGGCAAAAGCCTCGAGTTTGGCCATGGTACC

a The genomic positions for the primers are based on GenBank accession number HQ315835.
b The restriction enzyme sites used for cloning are underlined.
